# Transcriptional evidence for the "Reverse Warburg Effect" in human breast cancer tumor stroma and metastasis: Similarities with oxidative stress, inflammation, Alzheimer's disease, and "Neuron-Glia Metabolic Coupling"

**DOI:** 10.18632/aging.100134

**Published:** 2010-03-31

**Authors:** Stephanos Pavlides, Aristotelis Tsirigos, Iset Vera, Neal Flomenberg, Philippe G. Frank, Mathew C. Casimiro, Chenguang Wang, Richard G. Pestell, Ubaldo E. Martinez-Outschoorn, Anthony Howell, Federica Sotgia, Michael P. Lisanti

**Affiliations:** ^1^ Departments of Stem Cell Biology & Regenerative Medicine, and Cancer Biology, Kimmel Cancer Center, Thomas Jefferson University, Philadelphia, PA; ^2^ The Jefferson Stem Cell Biology and Regenerative Medicine Center, Kimmel Cancer Center, Thomas Jefferson University, Philadelphia, PA; ^3^Computational Genomics Group, IBM Thomas J. Watson Research Center, Yorktown Heights, NY; ^4^Department of Microbiology and Immunology, Albert Einstein College of Medicine, New York, NY; ^5^ Department of Medical Oncology, Kimmel Cancer Center, Thomas Jefferson University, Philadelphia, PA; ^6^ Manchester Breast Centre & Breakthrough Breast Cancer Research Unit, Paterson Institute for Cancer Research; School of Cancer, Enabling Sciences and Technology, Manchester Academic Health Science Centre, University of Manchester, UK

**Keywords:** caveolin-1, tumor stroma, oxidative stress, hypoxia, inflammation, mitochondrial dysfunction, Alzheimer's disease, neuron-glia metabolic coupling

## Abstract

Caveolin-1 (-/-) null stromal cells are a novel genetic model for cancer-associated fibroblasts and myofibroblasts. Here, we used an unbiased informatics analysis of transcriptional gene profiling to show that Cav-1 (-/-) bone-marrow derived stromal cells bear a striking resemblance to the activated tumor stroma of human breast cancers. More specifically, the transcriptional profiles of Cav-1 (-/-) stromal cells were most closely related to the primary tumor stroma of breast cancer patients that had undergone lymph-node (LN) metastasis. This is consistent with previous morphological data demonstrating that a loss of stromal Cav-1 protein (by immuno-histochemical staining in the fibroblast compartment) is significantly associated with increased LN-metastasis. We also provide evidence that the tumor stroma of human breast cancers shows a transcriptional shift towards oxidative stress, DNA damage/repair, inflammation, hypoxia, and aerobic glycolysis, consistent with the "Reverse Warburg Effect".  Finally, the tumor stroma of "metastasis-prone" breast cancer patients was most closely related to the transcriptional profiles derived from the brains of patients with Alzheimer's disease. This suggests that certain fundamental biological processes are common to both an activated tumor stroma and neuro-degenerative stress.  These processes may include oxidative stress, NO over-production (peroxynitrite formation), inflammation, hypoxia, and mitochondrial dysfunction, which are thought to occur in Alzheimer's disease pathology.  Thus, a loss of Cav-1 expression in cancer-associated myofibroblasts may be a protein biomarker for oxidative stress, aerobic glycolysis, and inflammation, driving the "Reverse Warburg Effect" in the tumor micro-environment and cancer cell metastasis.

## Introduction

Recently, we identified a loss of stromal caveolin-1 (Cav-1) as a novel biomarker for the cancer-associated fibroblast phenotype in human breast cancers [1].  More specifically, when fibroblasts were isolated from human breast cancers, 8 out of 11 patients showed >2-fold reduction in Cav-1 protein expression, relative normal matched fibroblasts prepared from the same patients [1].  Furthermore, detailed phenotypic analysis of mammary fibroblasts derived from Cav-1 (-/-) null mice revealed that they share numerous properties with cancer-associated fibroblasts, such as constitutively active TGFbeta signaling, and that they have the ability to promote normal mammary epithelial cells to undergo an EMT (epithelial-mesenchymal transition) [2].

To determine if loss of stromal Cav-1 has prognostic value, we performed a series of independent biomarker studies [3,4].  Using a cohort of 160 breast cancer patients, with nearly 20 years of follow-up data, we showed that a loss of stromal Cav-1 (in the fibroblast compartment) is a powerful single independent predictor early tumor recurrence, lymph node metastasis, tamoxifen-resistance, and poor clinical outcome [4].  As the prognostic value of a loss of stromal Cav-1 was independent of epithelial marker status, it appears that a loss of Cav-1 has predictive value in all the different epithelial subtypes of human breast cancer, including ER+, PR+, HER2+, and triple-negative patients [4].  The high predictive value of a loss of stromal Cav-1 was also independently validated by another independent laboratory, using a second independent breast cancer patient cohort [5].

A loss of stromal Cav-1 also appears to play a role in tumor initiation and progression [6]. Using a DCIS patient cohort, in which patients were treated with wide-excision, but without any chemo- or radio-therapy, we also evaluated the prognostic value of stromal Cav-1 [6].  In this DCIS patient cohort, a loss of stromal Cav-1 was specifically associated with DCIS recurrence and invasive progression.  100% of the patients with a loss of stromal Cav-1 underwent recurrence, and 80% of these patients progressed to invasive disease, namely frank invasive ductal carcinoma [6]. Similar  results were also independently obtained in human prostate cancers, where a loss of stromal Cav-1 was specifically associated with advanced prostate cancer, tumor progression, and metastatic disease [7].

To begin to understand the mechanism(s) underlying the lethality of a loss of Cav-1 in cancer-associated fibroblasts, we turned to Cav-1 (-/-) deficient mice as a model system.

For this purpose, we isolated bone marrow derived stromal cells from WT and Cav-1 (-/-) deficient mice, as cancer-associated fibroblasts are thought to evolve from mesenchymal stem cells [8]. These cells were then subjected to unbiased proteomic and genome-wide transcriptional analysis.  Interestingly, proteomic analysis revealed the upregulation of i) 8 myofibroblast markers (including vimentin, calponin, and tropomyosin), ii) 8 glycolytic enzymes (including PKM2 and LDHA), and iii) 2 markers of oxidative stress (peroxiredoxin1 and catalase) [8]. The glycolytic phenotype of Cav-1 (-/-) null stromal cells was also supported by transcriptional analysis, as most of the proteins that were found to be upregulated by proteomics, were also transcriptionally upregulated [8]. Based on these findings, we proposed a new model to understand the role of the Warburg effect ("aerobic glycolysis") in tumor metabolism. We hypothesized that glycolytic cancer-associated fibroblasts promote tumor growth by the secretion of energy-rich metabolites (such as pyruvate and lactate) that could then be taken up by adjacent epithelial cancer cells, where they would be incorporated into the tumor cell's TCA cycle, leading to enhanced ATP production [8]. This would provide a feed-forward mechanism by which glycolytic fibroblasts could promote tumor growth, progression, and metastasis. Because the Warburg effect was previously thought to be largely confined to tumor cells, and not to the cancer-associated fibroblast compartment, we have termed this new idea "The Reverse Warburg Effect" [8].

In order to determine which transcriptional programs are activated in Cav-1 (-/-) stromal cells, we performed an extensive bioinformatics analysis of our genome-wide profiling data [9]. This informatics analysis revealed that a loss of Cav-1 (-/-) in stromal cells drives ROS production and oxidative stress [9]. This, in turn, results in the activation of key transcription factor, such as HIF and NF-kB, which can then drive aerobic glycolysis and inflammation in the tumor micro-environment [9]. This could provide a molecular basis for understanding the lethality of a loss of stromal Cav-1 in human breast cancer patients.

Here, we have used a bioinformatics approach to determine whether similar "Warburg-like" transcrip-tional profiles exist in the tumor stroma isolated from human breast cancers.  For this purpose, we analyzed an existing data set in which the tumor stroma was isolated away from adjacent breast cancer cells using laser-capture micro-dissection [10].  We now provide new evidence for the existence of the "Reverse Warburg Effect" in human tumor stroma from breast cancer patients. More specifically, the tumor stroma of human breast cancers shows a transcriptional shift towards oxidative stress, DNA damage/repair, inflammation, hypoxia, and aerobic glycolysis, supporting with the "Reverse Warburg Effect". Consistent with the idea that oxidative stress in the tumor stroma is a driving factor in promoting tumor progression and metastasis, we also show that the tumor stroma of human breast cancers overlaps significantly with the transcriptional profiles associated with Alzheimer's brain disease.

Finally, the "Reverse Warburg Effect" is strikingly similar to the theory of "Neuon-Glia Metabolic Coupling" [11-18], which was proposed more than 10 years ago to explain metabolic changes associated with normal synaptic transmission, which may be exacerbated during neuronal stress and neuronal degeneration, as in Alzheimer's disease. In "Neuron-Glia Metabolic Coupling", astrocytes undergo aerobic glycolysis, secrete energy-rich metabolites (pyruvate and lactate), and neurons then take up these metabolites and use them in the neuronal TCA cycle to generate high amounts of ATP. Thus, we propose that "The Reverse Warburg Effect" we observe could also be more broadly termed "Epithelial-Stromal Metabolic Coupling".

As such, tumors may be initiating a survival mechanism that is normally used by the brain during stress. Interestingly, myofibroblasts and mesenchymal stem cells are known to often express GFAP (glial fibrillary acidic protein) [19-21], an intermediate filament protein that is thought to be relatively specific for astrocytes in the central nervous system. Here, we see that GFAP is upregulated in the "tumor stroma" and in the stroma of "metastasis-prone" breast cancer patients. Thus, possible similarities between astrocytes and myo-fibroblasts/cancer-associated fibroblasts should be further explored.

## Results

### Transcriptional comparison of Cav-1 (-/-) stromal cells with human breast cancer stroma

Previously, we subjected Cav-1 (-/-) bone marrow derived stromal cells, and their wild-type counter-parts to genome-wide transcriptional profiling [8].  Because such a large number of gene transcript levels are changed, we focused on the gene transcripts that are upregulated. We speculated that these Cav-1 (-/-) stromal gene profiles might also overlap with the transcriptional stromal profiles obtained from human breast cancers.

To test this hypothesis directly, we obtained the transcriptional profiles of a large data set of human breast cancer patients [10] whose tumors were subjected to laser-capture micro-dissection, to selectively isolate the tumor stroma. Based on this data set [10], we then generated three human breast cancer stromal genes lists:

1) Tumor Stroma vs. Normal Stroma List - Compares the transcriptional profiles of tumor stroma obtained 53 patients to normal stroma obtained from 38 patients. Genes transcripts that were consistently upregulated in tumor stroma were selected and assigned a p-value, with a cut-off of p <0.05 (contains 6,777 genes) (Supplementary Table 1).

2) Recurrence Stroma List - Compares the transcript-tional profiles of tumor stroma obtained from 11 patients with tumor recurrence to the tumor stroma of 42 patients without tumor recurrence. Genes transcripts that were consistently upregulated in the tumor stroma of patients with recurrence were selected and assigned a p-value, with a cut-off of p <0.05 (contains 3,354 genes) (Supplementary Table 2).

3)Lymph-node (LN) Metastasis Stroma List - Compares the transcriptional profiles of tumor stroma obtained from 25 patients with LN metastasis to the tumor stroma of 25 patients without LN metastasis. Genes transcripts that were consistently upregulated in the tumor stroma of patients with LN metastasis were selected and assigned a p-value, with a cut-off of p <0.05 (contains 1,182 genes) ( Supplementary Table 3).

**Figure 1.**
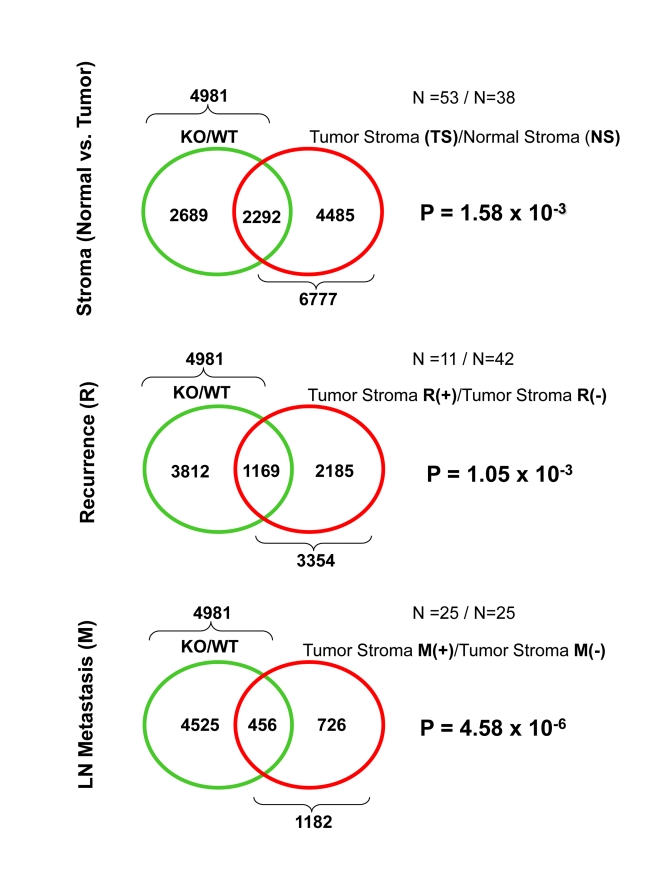
Venn diagrams for the transcriptional overlap between Cav-1 (-/-) stromal cells and tumor stroma from breast cancer patients.

These three gene lists were then individually intersected with the transcriptional profile of Cav-1 (-/-) null stromal cells [8]. The results of these intersections are presented in Figure 1, as Venn diagrams. Most important- ly, significant overlap was seen with all three gene lists. Greater than 2,000 genes were common between the Cav-1 (-/-) stromal gene list and the gene transcripts upregulated in breast cancer tumor stroma (p = 1.6 x 10^-3^).  Also, more than 1,000 gene transcripts were common between the Cav-1 (-/-) stromal gene list and the gene transcripts upregulated in the breast cancer tumor stroma of patients with tumor recurrence (p = 1 x 10^-3^). Finally, nearly 500 genes were commonly upregulated between Cav-1 (-/-) stromal cells and the breast cancer tumor stroma of patients with LN metastasis (p = 4.6 x 10^-6^).  Thus, the transcriptional profiles of Cav-1 (-/-) stromal cells are most  ignificantly related to the tumor stroma of patients with LN-metastasis. Independently, our previous data demonstrated that a loss of stromal Cav-1 protein expression (by immuno-histochemistry) in human breast cancers is specifically associated with a 2.6-fold increase in the number of tumor cell positive lymph nodes (LN-metastasis) [3,4].

The top 100 most significant gene transcripts for all three human breast cancer stromal gene lists, including their transcriptional intersection with Cav-1 (-/-) stromal cells, is included in Supplementary Tables 3, 4, and 5.

As Cav-1 (-/-) stromal cells are a genetic model of activated myofibroblasts [2] which biosynthetically secrete more collagen, and fibrosis is a critical risk factor for poor clinical outcome in human breast cancer patients [3], we also looked at the potential overlap been the expression of collagen gene transcripts (See Table 1). Thirty-five collagen gene transcripts were specifically upregulated in tumor stroma; 16 were upregulated in "recurrence-prone" stroma; and only 1 was upregulated in "metastasis-prone" stroma.  In all three cases, there was striking overlap with the collagen gene transcripts upregulated in Cav-1 (-/-) stromal cells, as indicated in bold (24 out of 35 transcripts; 12 out of 16 transcripts; and 1 out of 1 transcript; See Table 1).

Cav-1 (-/-) stromal cells have also been previously subjected to extensive analysis via an unbiased proteomics approach [8,24].   We next intersected these proteomic results with the three human breast cancer stromal gene lists.  The results of this intersection are shown in Table 2. Note that many of the proteins that are upregulated in Cav-1 (-/-) stromal cells are also transcriptionally upregulated in the stroma of human breast cancer patients. Most notably, there was a strong association between the metabolic enzymes that were upregulated in Cav-1 (-/-) stromal cells and the "recurrence-prone" and "metastasis-prone" stromal gene lists.

### Validating the "Reverse Warburg Hypothesis" in human breast cancer stroma

Recently, based on the unbiased proteomic and transcriptional analysis of Cav-1 (-/-) stromal cells, we have proposed that tumor stromal fibroblasts may undergo aerobic glycolysis [8]. We have termed this new idea the "Reverse Warburg Effect" [8].

Transcriptional analysis of Cav-1 (-/-) stromal cells [9] indicated that the "Reverse Warburg Effect" is associated with transcriptional over-expression of glycolysis-associated genes, HIF-target genes [25], NF-kB target genes [26], genes associated with the response to oxidative stress (GO_0006979), as well as the concomitant compensatory transcriptional upregulation of mitochondrial associated genes (GO_0005739) [9].

**Table 1.** Collagen gene expression in the human breast cancer stromal gene lists. Genes intersecting with the Cav-1 (-/-) bone marrow derived stromal gene list are shown in **bold**.

**Tumor Stroma Associated (24 of 35 collagen genes) **		**P-value **
**Col11a1**	**collagen, type XI, alpha 1 **	**1.51E-73**
Col8a1	collagen, type VIII, alpha 1	1.11E-51
Col10a1	collagen, type X, alpha 1	2.37E-42
Col12a1	collagen, type XII, alpha 1	6.40E-34
Col5a2	collagen, type V, alpha 2	7.78E-33
**Col5a1**	**collagen, type V, alpha 1 **	**2.54E-31**
**Col1a2**	**collagen, type I, alpha 2 **	**1.07E-27**
**Col3a1**	**collagen, type III, alpha 1 **	**3.32E-27**
**Col4a5**	**collagen, type IV, alpha 5 **	**6.04E-23**
Col8a2	collagen, type VIII, alpha 2	1.78E-22
**Col6a3**	**collagen, type VI, alpha 3 **	**3.87E-19**
**Col6a1**	**collagen, type VI, alpha 1 **	**8.97E-19**
**Col9a1**	**collagen, type IX, alpha 1 **	**3.05E-18**
Col17a1	collagen, type XVII, alpha 1	4.11E-18
**Col4a6**	**collagen, type IV, alpha 6 **	**2.50E-17**
**Col1a1**	**collagen, type I, alpha 1 **	**3.20E-17**
Col25a1	collagen, type XXV, alpha 1	7.13E-17
**Col5a3**	**collagen, type V, alpha 3 **	**1.17E-16**
Col20a1	collagen, type XX, alpha 1	2.35E-16
**Col16a1**	**collagen, type XVI, alpha 1 **	**3.77E-16**
Col13a1	collagen, type XIII, alpha 1	4.27E-14
**Col24a1**	**collagen, type XXIV, alpha 1 **	**4.07E-13**
Col15a1	collagen, type XV, alpha 1	2.00E-12
Col4a4	collagen, type IV, alpha 4	5.55E-12
**Col4a2**	**collagen, type IV, alpha 2 **	**1.17E-11**
**Col18a1**	**collagen, type XVIII, alpha 1 **	**5.00E-11**
**Col9a2**	**collagen, type IX, alpha 2 **	**5.30E-11**
**Col14a1**	**collagen, type XIV, alpha 1 **	**4.92E-10**
**Col23a1**	**collagen, type XXIII, alpha 1 **	**7.52E-08**
**Col11a2**	**collagen, type XI, alpha 2**	**3.90E-07**
**Col2a1**	**collagen, type II, alpha 1 **	**6.22E-07**
**Col27a1**	**collagen, type XXVII, alpha 1**	**4.93E-06**
**Col4a3**	**collagen, type IV, alpha 3 **	**1.21E-05**
**Col19a1**	**collagen, type XIX, alpha 1 **	**1.90E-05**
**Col4a1**	**collagen, type IV, alpha 1 **	**4.37E-02**
**Recurrence-Prone Stroma (12 of 16 collagen genes) **		
Col13a1	collagen, type XIII, alpha 1	4.16E-05
Col20a1	collagen, type XX, alpha 1	4.34E-05
**Col3a1**	**collagen, type III, alpha 1 **	**8.00E-05**
**Col11a1**	**collagen, type XI, alpha 1**	**2.84E-04**
**Col1a1**	**collagen, type I, alpha 1 **	**2.46E-03**
**Col11a2**	**collagen, type XI, alpha 2**	**4.63E-03**
Col8a2	collagen, type VIII, alpha 2	8.91E-03
**Col23a1**	**collagen, type XXIII, alpha 1 **	**1.05E-02**
**Col4a2**	**collagen, type IV, alpha 2 **	**1.51E-02**
**Col9a1**	**collagen, type IX, alpha 1 **	**1.58E-02**
**Col4a5**	**collagen, type IV, alpha 5 **	**1.85E-02**
**Col14a1**	**collagen, type XIV, alpha 1 **	**1.94E-02**
**Col2a1**	**collagen, type II, alpha 1 **	**2.06E-02**
Col10a1	collagen, type X, alpha 1	2.08E-02
**Col9a2**	**collagen, type IX, alpha 2 **	**2.97E-02**
**Col19a1**	**collagen, type XIX, alpha 1 **	**3.90E-02**
**Metastasis-Prone Stroma (1 of 1 collagen genes) **		
**Col6a1**	**collagen, type VI, alpha 1 **	**4.00E-02**

**Table 2.** Intersection of Cav-1 (-/-) stromal proteomics with the human breast cancer stromal gene lists. Includes proteins upregulated in Cav-1 (-/-) bone marrow derived stromal cells (ref # 8), Cav-1 (-/-) mouse embryo fibroblasts (ref # 24), and Cav-1 (-/-) mammary fat pad.  P values listed are from the Human Breast Cancer Stromal Gene Lists.  Genes in **bold** are associated with metabolism.

**Gene **	**Description **	**Tumor Stroma **	**Recurrence-Prone **	**Metastasis-Prone **
Capg	capping protein (actin filament), gelsolin-like	4.18e-38	4.07e-03	
Sparc	secreted acidic cysteine rich glycoprotein	1.49e-35		
Arhgdib	Rho, GDP dissociation inhibitor (GDI) beta	3.92e-32		
Gpd2	**glycerol phosphate dehydrogenase 2, mitochondrial **	**1.39e-29**		
**Upp1**	**uridine phosphorylase 1 **	**2.77e-28**		
Col3a1	collagen, type III, alpha 1	3.30e-27	8.00e-05	
Col1a2	collagen, type I, alpha 2	1.07e-27		
Tpm1	tropomyosin 1, alpha	2.20e-26	5.23e-07	
Sh3bgrl3	SH3 domain binding glutamic acid-rich protein-like 3	4.35e-24		
Col1a1	collagen, type I, alpha 1	3.20e-17	2.46e-03	
Eef1d	eukaryotic translation elongation factor 1 delta (guanine nucleotide exchange protein)	2.00e-12		
Nme2	non-metastatic cells 2, protein (NM23B) expressed in	2.39e-09		
Sncg	synuclein, gamma (breast cancer-specific protein 1)	8.86e-08		
**Ldhc**	**lactate dehydrogenase C **	**1.26e-07**	**1.78e-03**	
Myl1	myosin, light chain 1, alkali; skeletal, fast	3.60e-07		
Gsn	gelsolin	6.30e-05		
**Ckm **	**creatine kinase, muscle **	**3.88e-05**		
Tpm2	tropomyosin 2, beta	1.38e-03	2.22e-03	
Cnn2	calponin 2		2.26e-02	
Fth1	ferritin, heavy polypeptide 1		2.72e-02	
**Pdha1 **	**pyruvate dehydrogenase E1 alpha subunit**		**2.85e-02**	
**Pgk1**	**phosphoglycerate kinase 1**		**3.21e-02**	
**Eno3**	**enolase 3, beta muscle**			**1.29e-03 **
**Aldoa**	**aldolase A, fructose-bisphosphate**			**1.69e-03**
Afp	alpha fetoprotein			3.06e-02
**Pkm2**	**pyruvate kinase, muscle**			**3.73e-02**
Alb	albumin			3.95e-02
**Pgd**	**phosphogluconate dehydrogenase**			**4.19e-02**
Serpinb2	serine (or cysteine) peptidase inhibitor, clade B, member 2			4.27e-02
Eef2	eukaryotic translation elongation factor 2			4.41e-02

**Table 3.** Intersection of human breast cancer stromal gene sets with gene sets related to the "Reverse Warburg Effect".

	**Glycolysis**	**HIF Targets**	**Mitochondrial Genes**	**NF-kB Targets**	**Ox Stress**	**Alzheimer's**
**Stromal Gene Set **						
**Tumor Stroma**	**19**	**213**	**233**	**199**	**51**	**676**
**Recurrence-Prone**	**10**	**108**	**120**	**86**	**22**	**338**
**Metastasis-Prone**	**7**	**42**	**68**	**32**	**9**	**145**
						

Table 3 shows that all of these gene sets are well-represented in tumor stroma, "recurrence-prone" stroma, and the "metastasis-prone" stroma of human breast cancer patients (See also SupplmentalTables 7, 8, and 9 for detailed gene lists).

It is important to note that these breast cancer stromal gene lists also include Cxcl12, a known HIF-target gene [25], that is transcriptionally-upregulated ~5-fold in Cav-1 (-/-) stromal cells [8].

### The "Reverse Warburg Effect" and similarities with Alzheimer's disease

We have previously shown that the transcriptional profiles of Cav-1 (-/-) stromal cells significantly ovelap with the transcriptional profiles obtained from the analysis of Alzheimers disease brain [9]. We believe this is functionally due to the activation of similar biological processes in both "The Reverse Warburg Effect" and Alzheimer's disease [9], including oxidative stress, NO over-production (peroxynitrite formation), inflammation, hypoxia, and mitochondrial dysfunction [27].

**Figure 2.**
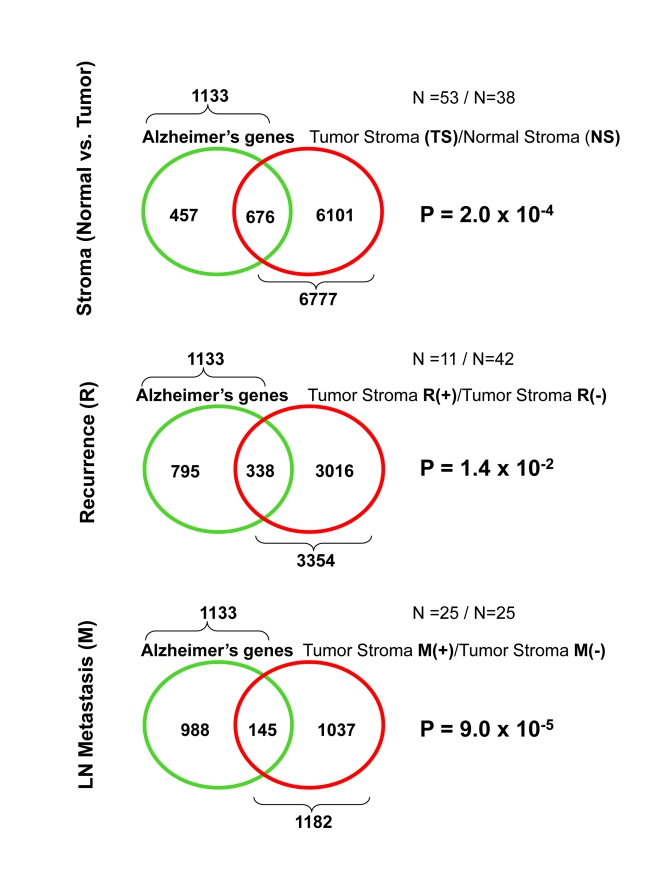
Venn diagrams for the transcriptional overlap between Alzheimer's disease brain and tumor stroma from breast cancer patients.

Thus, here, we independently evaluated the association between Alzheimer's disease and human breast cancer tumor stroma. These transcriptional overlaps are enumerated in Table 3, and are illustrated schematically as Venn diagrams in Figure 2.  Detailed gene lists are provided in Supplemental Tables 7, 8, and 9.

Interestingly, as predicted, the genes that are transcriptionally upregulated in Alzheimer's disease significantly overlap with tumor stroma, "recurrence-prone" stroma, and "metastasis-prone" stroma. This clearly functionally links Alzheimer's disease with the human breast cancer tumor stroma.

As with the gene profiles of Cav-1 (-/-) stromal cells, the Alzheimer's disease profiles were most significantly associated with the "metastasis-prone" stromal gene set (p = 9 x 10^-5^).

### Detailed analysis of the "Metastasis-Prone" stromal gene set

Next, we examined the possible overlap of the "metastasis-prone" stromal gene set with other existing transcriptional profiles, using gene-set enrichment analysis.

Our results are shown in Table 4. Briefly, we see that the "metastasis-prone" stromal gene set is associated with a number of interesting biological processes, including cell cycle progression and survival, DNA damage/repair, scleroderma, "stemness", aging and oxidative stress, Alzheimer's disease, decreased DNA-methylation, tamoxifen-resistance, metastasis, Myc-associated target genes, inflammation (NF-kB/STAT), TGFbeta signaling and myofibroblast differentiation, hypoxia and HIF signaling, mitochondrial function, and liver-specific gene transcription.

**Table 4.** Comparative results for wild type N2 vs. *nth-1;xpa-1.*

**Data Set**	**Description**	**P-value**
**Cell Cycle Progression and Survival **		
MORF_ANP32B	Neighborhood of ANP32B acidic (leucine-rich) nuclear phosphoprotein 32 family, member B in the MORF expression compendium	2.34E-08
MORF_CSNK2B	Neighborhood of CSNK2B casein kinase 2, beta polypeptide in the MORF expression compendium	3.97E-06
MORF_PCNA	Neighborhood of PCNA proliferating cell nuclear antigen in the MORF expression compendium	6.66E-06
MORF_DEK	Neighborhood of DEK oncogene (DNA binding) in the MORF expression compendium	4.97E-05
SHIPP_FL_VS_DLBCL_DN	Genes upregulated in diffuse B-cell lymphomas (DLBCL) and downregulated in follicular lymphoma (FL) (fold change of at least 3)	1.17E-04
MORF_RAN	Neighborhood of RAN, member RAS oncogene family in the MORF expression compendium	2.14E-04
MORF_SKP1A	Neighborhood of SKP1A S-phase kinase-associated protein 1A (p19A) in the MORF expression Compendium	2.28E-04
TGANTCA_V$AP1_C	Genes with promoter regions [-2kb,2kb] around transcription start site containing the motif TGANTCA which matches annotation for JUN: jun oncogene	4.47E-04
GNF2_RAN	Neighborhood of RAN, member RAS oncogene family in the GNF2 expression compendium	8.76E-04
GCM_ANP32B	Neighborhood of ANP32B acidic (leucine-rich) nuclear phosphoprotein 32 family, member B in the GCM expression compendium	1.92E-03
MITOSIS	Genes annotated by the GO term GO:0007067. Progression through mitosis, the division of the eukaryotic cell nucleus to produce two daughter nuclei that, usually, contain the identical chromosome complement to their mother.	1.10E-02
SMITH_HTERT_UP	Genes upregulated by telomerase	1.90E-02
CHANG_SERUM_RESPONSE_UP	CSR (Serum Response) signature for activated genes (Stanford)	2.13E-02
**DNA Damage and Repair **		
CIS_XPC_UP	Increased expression in XPC-defective fibroblasts, compared to normal fibroblasts, following treatment with cisplatin	2.08E-07
MORF_RAD23A	Neighborhood of RAD23A, RAD23 homolog A (S. cerevisiae) in the MORF expression compendium; nucleotide excision repair (NER)	3.01E-07
MORF_G22P1	Neighborhood of G22P1 NULL in the MORF expression compendium a.k.a., XRCC6 Gene, X-ray repair complementing defective repair in Chinese hamster cells 6; a.k.a., thyroid autoantigen 70kD (Ku antigen)	6.29E-07
MORF_XRCC5	Neighborhood of XRCC5 X-ray repair complementing defective repair in Chinese hamster cells 5 (double-strand-break rejoining; Ku autoantigen, 80kDa) in the MORF expression compendium	2.48E-04
MORF_EIF3S6	Neighborhood of EIF3S6 eukaryotic translation initiation factor 3, subunit 6 48kDa in the MORF expression compendium; murine mammary tumor integration site 6 (oncogene homolog)	3.61E-04
GNF2_G22P1	Neighborhood of G22P1 NULL in the GNF2 expression compendium	4.90E-04
MORF_RAD21	Neighborhood of RAD21 RAD21 homolog (S. pombe) in the MORF expression compendium	1.28E-03
UVC_LOW_A2_UP	Up-regulated at 6-12 hours following treatment of WS1 human skin fibroblasts with UVC at a low dose (10 J/m^2) (cluster a2)	3.90E-03
UVB_NHEK3_C7	Regulated by UV-B light in normal human epidermal keratinocytes, cluster 7	6.80E-03
UVC_LOW_ALL_UP	Up-regulated at any timepoint following treatment of WS1 human skin fibroblasts with UVC at a low dose (10 J/m^2) (clusters a1-a4)	7.84E-03
UVB_NHEK3_C4	Regulated by UV-B light in normal human epidermal keratinocytes, cluster 4	9.69E-03
UVB_NHEK1_C4	Upregulated by UV-B light in normal human epidermal keratinocytes, cluster 4	9.75E-03
UVB_NHEK3_ALL	Regulated by UV-B light in normal human epidermal keratinocytes	1.00E-02
**Scleroderma **		
MORF_FBL	Neighborhood of FBL fibrillarin in the MORF expression compendium a.k.a., 34 kDa nucleolar scleroderma antigen, or RNA, U3 small nucleolar interacting protein 1	7.49E-07
**Stem Cells **		
STEMCELL_NEURAL_UP	Enriched in mouse neural stem cells, compared to differentiated brain and bone marrow cells	6.93E-06
STEMCELL_EMBRYONIC_UP	Enriched in mouse embryonic stem cells, compared to differentiated brain and bone marrow cells	1.97E-04
LIN_WNT_UP	Genes up-regulated by APC in SW480 (colon cancer)	7.50E-04
HSC_INTERMEDIATE PROGENITORS_FETAL	Up-regulated in mouse hematopoietic intermediate progenitors from fetal liver (Intermediate Progenitors Shared + Fetal)	3.75E-03
HSA04310_WNT_ SIGNALING_PATHWAY	Genes involved in Wnt signaling pathway	7.14E-03
HSA04330_NOTCH_ SIGNALING_PATHWAY	Genes involved in Notch signaling pathway	1.28E-02
V$TCF4_Q5	Genes with promoter regions [-2kb,2kb] around transcription start site containing the motif SCTTTGAW which matches annotation for TCF4: transcription factor 4	1.49E-02
HSC_HSCANDPROGENITORS _SHARED	Up-regulated in mouse hematopoietic stem cells and progenitors from both adult bone marrow and fetal liver (Cluster iii, HSC and Progenitors Shared)	2.00E-02
HSC_HSCANDPROGENITORS _FETAL	Up-regulated in mouse hematopoietic stem cells and progenitors from fetal liver (HSC and Progenitors Shared)	2.09E-02
HSC_INTERMEDIATE PROGENITORS_SHARED	Up-regulated in mouse hematopoietic intermediate progenitors from both adult bone marrow and fetal liver (Cluster v, Intermediate Progenitors Shared)	2.15E-02
MAMMARY_DEV_UP	Up-regulated in the intact developing mouse mammary gland; higher expression in 5/6 week pubertal glands than in 3 week, mid-pregnant, lactating, involuting or resuckled glands	2.15E-02
**Aging, Alzheimer's Disease, and Oxidative Stress **		
MORF_SOD1	Neighborhood of SOD1 superoxide dismutase 1, soluble (amyotrophic lateral sclerosis 1 (adult)) in the MORF expression compendium	1.98E-05
ALZHEIMERS_DISEASE_UP	Upregulated in correlation with overt Alzheimer's Disease, in the CA1 region of the hippocampus	9.05E-05
MORF_JUND	Neighborhood of JUND jun D proto-oncogene in the MORF expression compendium	2.87E-03
**Regulation of DNA Methylation **		
MORF_HDAC1	Neighborhood of HDAC1 histone deacetylase 1 in the MORF expression compendium	9.91E-06
TSA_PANC50_UP	50 most interesting genes upregulated by TSA treatment in at least one of four pancreatic cancer cell lines, but not in normal (HPDE) cells	4.32E-04
MORF_HAT1	Neighborhood of HAT1 histone acetyltransferase 1 in the MORF expression compendium	9.44E-04
**Breast Cancer Associated Tamoxifen-Resistance **		
MORF_NPM1	Neighborhood of NPM1 nucleophosmin (nucleolar phosphoprotein B23, numatrin) in the MORF expression compendium	1.73E-04
GCM_NPM1	Neighborhood of NPM1 nucleophosmin (nucleolar phosphoprotein B23, numatrin) in the GCM expression compendium	7.21E-03
GNF2_NPM1	Neighborhood of NPM1	1.24E-02
**Metastasis **		
MORF_NME2	Neighborhood of NME2 non-metastatic cells 2, protein (NM23B) expressed in in the MORF expression compendium	2.04E-03
MORF_MTA1	Neighborhood of MTA1 metastasis associated 1 in the MORF expression compendium	1.28E-02
CROMER_HYPOPHARYNGEAL_ MET_VS_NON_UP	Genes increased in metastatic hypopharyngeal cancer tumours	2.37E-02
**Myc-Associated Genes **		
CACGTG_V$MYC_Q2	Genes with promoter regions [-2kb,2kb] around transcription start site containing the motif CACGTG which matches annotation for MYC: v-myc myelocytomatosis viral oncogene homolog (avian)	2.05E-03
LEE_MYC_TGFA_UP	Genes up-regulated in hepatoma tissue of Myc+Tgfa transgenic mice	7.34E-03
LEE_MYC_UP	Genes up-regulated in hepatoma tissue of Myc transgenic mice	1.00E-02
MYC_ONCOGENIC_SIGNATURE	Genes selected in supervised analyses to discriminate cells expressing c-Myc oncogene from control cells expressing GFP.	1.00E-02
V$MYC_Q2	Genes with promoter regions [-2kb,2kb] around transcription start site containing the motif CACGTGS which matches annotation for MYC: v-myc myelocytomatosis viral oncogene homolog (avian)	1.26E-02
V$NMYC_01	Genes with promoter regions [-2kb,2kb] around transcription start site containing the motif NNCCACGTGNNN which matches annotation for MYCN: v-myc myelocytomatosis viral related oncogene, neuroblastoma derived (avian)	1.32E-02
FERNANDEZ_MYC_TARGETS	MYC target genes by ChIP in U-937,HL60 (leukemia),P493 (B-cell),T98G (glioblastoma),WS1 (fibroblast)	2.43E-02
**Inflammation/NF-kB/STAT Signaling **		
IL6_FIBRO_UP	Upregulated following IL-6 treatment in normal skin fibroblasts	2.05E-03
TNFALPHA_30MIN_UP	Upregulated 30min after TNF-alpha treatment of HeLa cells	2.23E-03
HESS_HOXAANMEIS1_UP	Genes upregulated in Hoxa9/Meis1 transduced cells vs control	6.31E-03
ST_INTERLEUKIN_13_PATHWAY	IL-13 is produced by Th2 cells on activation of the T cell antigen receptor, and by mast and basophil cells on activation of the IgE receptor.	9.22E-03
ST_IL_13_PATHWAY	Like IL-4, IL-13 is produced by Th2 cells on activation of the T cell antigen receptor, and by mast and basophil cells on activation of the IgE receptor.	9.45E-03
V$IRF_Q6	Genes with promoter regions [-2kb,2kb] around transcription start site containing the motif BNCRSTTTCANTTYY which matches annotation for IRF1: interferon regulatory factor 1	1.42E-02
TNFALPHA_ALL_UP	Upregulated at any timepoint after TNF-alpha treatment of HeLa cells	1.44E-02
**TGFbeta Signaling/Myofibroblast Differentiation/Fibrosis **		
GCM_ACTG1	Neighborhood of ACTG1 actin, gamma 1 in the GCM expression compendium	2.18E-03
TGFBETA_ALL_UP	Upregulated by TGF-beta treatment of skin fibroblasts, at any timepoint	6.80E-03
MYOD_BRG1_UP	Genes up-regulated following transduction of MyoD in NIH 3T3 cells that fail to acheive full induction with expression of a dominant-negative BRG1 allele	7.07E-03
MORF_ACTG1	Neighborhood of ACTG1 actin, gamma 1 in the MORF expression compendium	9.15E-03
MYOD_NIH3T3_UP	Up-regulated at 24 hours in NIH 3T3 murine fibroblasts following transduction with MyoD and incubation in differentiation medium	1.08E-02
POMEROY_DESMOPLASIC_VS_ CLASSIC_MD_UP	Genes expressed in desmoplastic medulloblastomas. (p < 0.01)	9.68E-03
TGFBETA_LATE_UP	Upregulated by TGF-beta treatment of skin fibroblasts only at 1-4 hrs (clusters 4-6)	2.36E-02
**Hypoxia/HIF Signaling/Mitochondrial Genes/Metabolism **		
HYPOXIA_REVIEW	Genes known to be induced by hypoxia	8.96E-03
HIF1_TARGETS	Hif-1 (hypoxia-inducible factor 1) transcripional targets	1.07E-02
HUMAN_MITODB_6_2002	Mitochondrial genes	1.08E-02
MITOCHONDRIA	Mitochondrial genes	1.28E-02
HYPOXIA_RCC_UP	Upregulated by hypoxia in VHL-rescued renal carcinoma cells (Fig. 3f+g)	1.42E-02
HSA00330_ARGININE_AND _PROLINE_METABOLISM	Genes involved in arginine and proline metabolism	2.20E-02
**Liver Specific Transcription **		
HSIAO_LIVER_SPECIFIC_GENES	Liver selective genes	1.04E-02

We have independently shown that many of these same biological processes are activated in Cav-1 (-/-) stromal cells [9], consistent with the idea that Cav-1 (-/-) stromal cells are a valid model for exploring the tumor-promoting effects of an activated tumor stromal micro-environment.

### Similarities of the Cav-1 (-/-) stromal gene set with transcriptional profiling data from ER-negative breast cancer

A comparison of the Cav-1 (-/-) stromal cell gene set with other existing transcriptional profiles also shows significant overlap with ER-negative human breast cancer (p = 8.96 x 10^-10^; BRCA_ER_NEG [28]). For this overlap analysis, UP genes from the Cav-1 (-/-) stromal data set with a fold-change of > 2.0 (KO/WT) and a P value of < 0.1 were utilized for comparison with existing gene sets in the data base.

**Figure 3.**
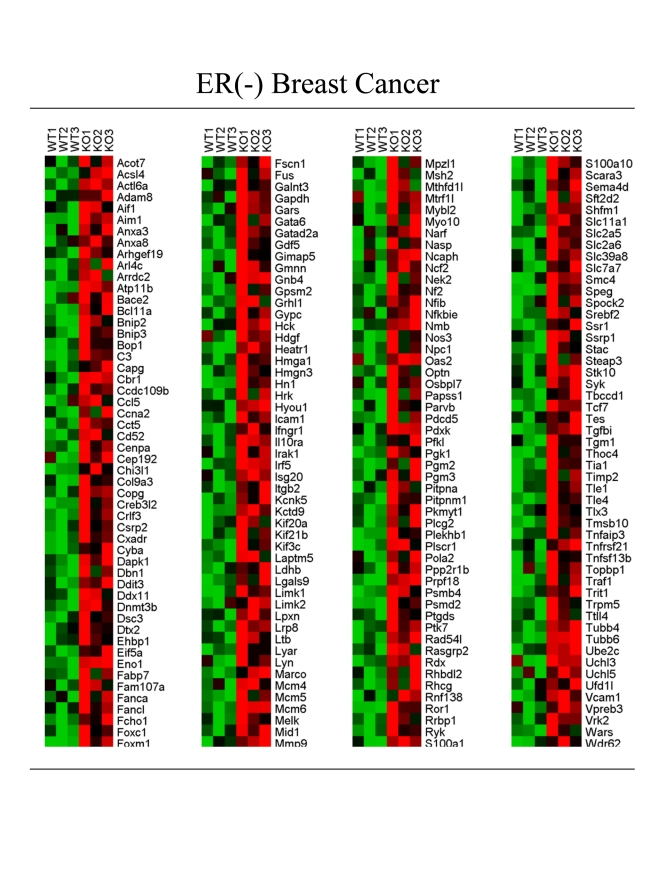
Transcriptional overlap of the Cav-1 (-/-) stromal gene set with ER-negative breast cancer. A HeatMap containing 205 intersecting genes is shown (FC >1.5; p <0.05). See also Supplementary Tables. FC, fold-change.

Interestingly, these tumors were not laser-capture micro-dissected, so this provides an indication that the Cav-1 (-/-) stromal gene set may also be well represented in the transcriptional profiles obtained from whole tumors. A HeatMap containing these intersecting genes is shown in Figure 3 (205 overlapping genes; FC > 1.5; p < 0.05). See also Supplementary Tables.

These include key overlapping genes associated with ***metabolism and glycolysis*** (Acot7, Acsl4, Eno1, Gapdh, Ldhb, Mtrf1l, Pfkl, Pgk1, Pgm2, Pgm3, Slc2a5, Slc2a6), ***hypoxia ***(Hyou1), ***the inflammatory response*** (Aif1, C3, Ccl5, Crlf3, Ifngr1, Il10ra, Irak1, Irf5, Isg20, Nfib, Nfkbie, Nos3, Tnfaip3, Tnfrsf21, Tnfsf13b, Traf1), ***myofibroblast differentiation and the extracellular matrix*** (Actl6a, Capg, Col9a3, Dnmt3b, Mmp9, Myo10, Spock2, Tgfbi, Tgm1, Timp2), as well as ***DNA-damage and repair*** (Ddit3, Rad54l).  These results are consistent with the existence of the "Reverse Warburg Effect" in ER-negative breast cancers.

Interestingly, it has been previously demonstrated that key secreted inflammatory factors, such as Aif1 (allograft inflammatory factor-1) (upregulated nearly 3-fold in Cav-1 (-/-) stromal cells; Supplementary Tables) promote NFkB-activation, the paracrine growth of ER-negative breast cancer cells [29], and are involved in the pathogenesis of pro-fibrotic diseases, such as scleroderma (systemic sclerosis) [30-32].

Similarly, Aif1 expression is highly-upregulated in the tumor stroma of human breast cancers (See Supplementary Table 1; p = 8.35 x 10^-24^).

## Discussion

Here, we provide compelling transcriptional evidence for the "Reverse Warburg Effect" in human breast cancer tumor stroma. Using an unbiased informatics analysis of transcriptional gene profiling, we show that Cav-1 (-/-) stromal cells bear a striking resemblance to the activated tumor stroma of human breast cancers. More specifically, the transcriptional profiles of Cav-1 (-/-) stromal cells were most closely related to the stroma of breast cancer patients that had undergone LN-metastasis. This is consistent with our previous data showing that a loss of stromal Cav-1 protein expression (by immuno-histochemistry) in human breast cancer tumor micro-arrays is specifically associated with increased LN-metastasis [3,4].

Moreover, we provide evidence that the tumor stroma of human breast cancers shows a transcriptional shift towards oxidative stress, DNA damage/repair, inflammation, hypoxia, and aerobic glycolysis. These findings are consistent with the "Reverse Warburg Effect" [8,9]. Notably, the tumor stroma of "metastasis-prone" breast cancer patients was also closely related to the transcriptional profiles derived from the brains of patients with Alzheimer's disease. As such, certain fundamental biological processes are common to both an activated tumor stroma and neuro-degenerative stress.  These key biological processes most likely include oxidative stress, NO over-production (peroxynitrite formation), inflammation, hypoxia, and mitochondrial dysfunction, which are all thought to drive Alzheimer's disease pathogenesis.

Thus, we avidly reviewed the literature regarding theories of neuronal functioning, neuronal stress, and neuro-degeneration, in the central nervous system and we stumbled upon the idea of "Neuron-Glia Metabolic Coupling" [11-18]  In this model, first proposed over 10 years ago, astrocytes shift towards aerobic glycolyis, secrete pyruvate and lactate, which is then taken-up by adjacent neurons and then "feeds" into the neuronal TCA cycle, resulting in increased neuronal oxidative mitochondrial metabolism, and higher ATP production in neurons. In essence, the astrocytes would function as support cells to "feed" the adjacent neuronal cells. Thus, "Neuron-Glia Metabolic Coupling" and the "Reverse Warburg Effect" are analogous biological processes, where the astrocytes are the cancer-associated fibroblasts and the neurons are the epithelial tumor cells.  As such, we propose that the "Reverse Warburg Effect" could also be more generally termed "Epithelial-Stromal Metabolic Coupling" or "Epithelial-Fibroblast Metabolic Coupling".

**Figure 4.**
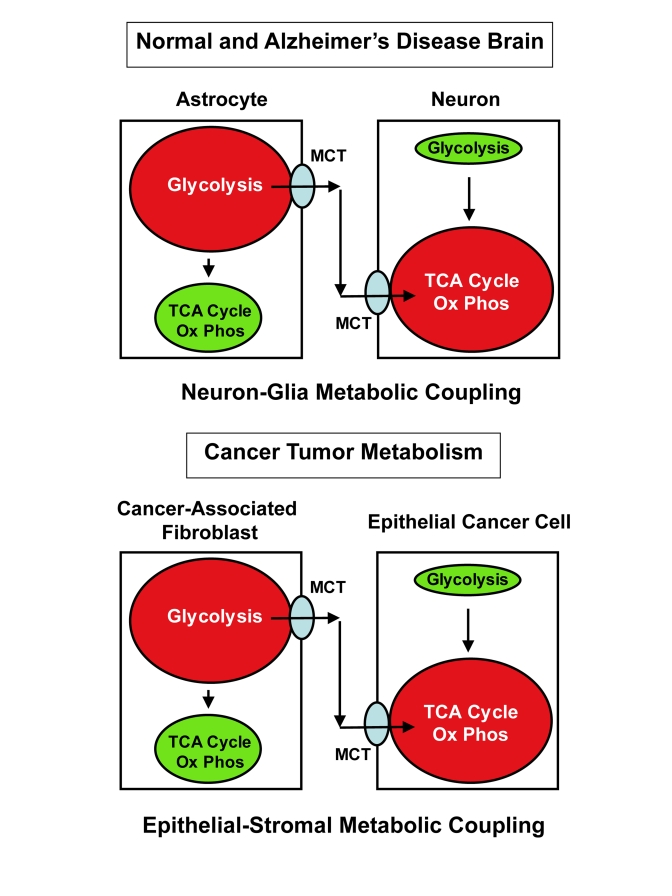
Comparisons between the "Reverse Warburg Effect" and "Neuron-Glia Metabolic Coupling", suggest "Epithelial-Stromal Metabolic Coupling". In "Neuron-Glia Metabolic Coupling", astrocytes take up more glucose, shift towards aerobic glycolyis, secrete pyruvate and lactate, which is then taken up by adjacent neurons and then "feeds" into the neuronal TCA cycle, resulting in increased neuronal oxidative mitochondrial metabolism, and higher ATP production in neurons. In essence, the astrocytes function as support cells to "feed" the adjacent neuronal cells. This schematic diagram shows that "Neuron-Glia Metabolic Coupling" and the "Reverse Warburg Effect" are analogous biological processes, where the astrocytes are the cancer-associated fibroblasts and the neurons are the epithelial tumor cells.  Thus, the "Reverse Warburg Effect" could also be more generally termed "Epithelial-Stromal Metabolic Coupling" or "Epithelial-Fibroblast Metabolic Coupling". This figure was partially re-drawn from Bonucelli et al. 2010, with permission [24]. MCT, mono-carboxylate transporter.

If these two processes are indeed analogous, then epithelial tumor cells have already learned to behave as neurons, using the stroma as a means of support and nourishment. Figure 4 directly compares "Neuron-Glia Metabolic Coupling" with the "Reverse Warburg effect" schematically.

Myofibroblasts and mesenchymal stem cells are known to often express GFAP (glial fibrillary acidic protein) [19-21], an intermediate filament protein that is thought to be relatively specific for astrocytes in the central nervous system.  Table 5 shows that GFAP and other glial-related gene transcripts are indeed upregulated in "tumor stroma" and in the stroma of "metastasis-prone" breast cancer patients. Thus, possible metabolic and functional similarities between CNS astrocytes and myofibroblasts/cancer-associated fibroblasts should be further explored.

Interestingly, in "Neuron-Glia Metabolic Coupling" the glycolytic shift in astrocytes is thought to be mediated by the secretion of glutamate (a neurotransmitter) from neurons. Then, astrocytes take up glutamate via high affinity sodium-dependent glutamate transporters, such as ***Slc1a2 and Slc1a3***. Importantly, one of these two glial-specific glutamate transporters (***Slc1a3***) is also transcriptionally over-expressed in the stroma of human breast cancer patients (Table 5). As such, the similarities between brain astrocytes, myofibroblasts, mesenchymal stem cells, and tumor stromal cells may be more extensive than we previously appreciated.

**Table 5.** Expression of glial-related genes in human breast cancer stromal gene sets. Gfap is highlighted in **bold** because it is also known to be a common marker of astrocytes, myo-fibroblasts, and mesenchymal stem cells.

**Gene**	**Description**	**Tumor Stroma **	**Recurrence -Prone Stroma**	**Metastasis-Prone Stroma**
Gcm1	glial cells missing homolog 1 (Drosophila)	6.50e-21	8.39e-04	
**Gfap**	**glial fibrillary acidic protein **	**1.64e-18**	**1.36e-03**	**2.28e-02**
Gfra2	glial cell line derived neurotrophic factor family receptor alpha 2	2.28e-17	3.58E-02	
Slc1a3	solute carrier family 1 (glial high affinity glutamate transporter), member 3	4.22e-17	5.70e-03	
Gfra3	glial cell line derived neurotrophic factor family receptor alpha 3	2.97e-16		
Gdnf	glial cell line derived neurotrophic factor	6.48e-14		
Gcm2	glial cells missing homolog 2 (Drosophila)	1.38e-05	2.06e-02	
Gfra4	glial cell line derived neurotrophic factor receptor alpha 4			1.02e-02

## Methods of analysis

Venn diagrams. In the Venn diagram of Figure 1, we show the intersections between the set of genes that are upregulated in Cav-1 (-/-) versus wild-type stromal cells [8] and three breast cancer gene sets [10].

(a)        the set of stromal genes that are upregulated in breast cancer tumor patients versus normal breast stroma; (b)        the set of stromal genes that are upregulated in recurrence positive versus recurrence negative breast cancer patients (c)        the set of stromal genes that are upregulated in lymph-node metastasis positive versus lymph-node metastasis negative breast cancer patients.

In the Venn diagram of Figure 2, we show the intersections between the set of genes that are upregulated in Alzheimer's brain disease [22] and the sets of genes (a)-(c) listed above. The p-values determining the significance of upregulation for each gene were computed using a one-sided t-test statistic (Tables 1, 2, and 5). For each pair (X,Y) of sets of genes, we also computed the probability (p-value) that the size of their intersection is less than or equal to the size of the intersection between set X and a randomly-chosen set of size equal to the size of set Y. This probability was calculated by applying the cumulative density function of the hypergeometric distribution on the size of set X, the size of set Y, the observed overlap between X and Y, and the total number of available genes.

Gene set enrichment analysis. For the functional analysis presented in Table 4, we used data from the Molecular Signatures Database (MsigDB [23]) which comprises a collection of gene sets: - collected from various sources such as online pathway databases, publications, and knowledge of domain experts, - comprising genes that share a conserved cis-regulatory motif across the human, mouse, rat, and dog genomes, - identified as co-regulated gene clusters by mining large collections of cancer-oriented microarray data, and - annotated by a common Gene Ontology (GO) term.

For our analysis we used the latest release of MSigDB database v2.5 (April 7, 2008), after converting all the gene names in the database into RefSeq gene IDs. After this preprocessing step, we chose the sub-collection of gene sets that was relevant to our study, and for each gene set X in that sub-collection, we computed the overlap between X and the set of genes Y that are upregulated in lymph-node metastasis positive versus lymph-node metastasis negative breast cancer patients (p-value ≤0.05). Then, we computed the probability (p-value) of the observed overlap between sets X and Y as described in the "Venn diagrams" section.

## Supplementary data

Supplementary Table 1 TumorStroma (Compatibility Mode).

Supplementary Table 2 Recurrence (Compatibility Mode).

Supplementary Table 3 Metastatis (Compatibility Mode).

Supplementary Table 4-10
