# Supplementary material for: Transcriptional evidence for the "Reverse Warburg Effect" in human breast cancer tumor stroma and metastasis: Similarities with oxidative stress, inflammation, Alzheimer's disease, and "Neuron-Glia Metabolic Coupling"
Source: Aging (Albany NY). 2010 Mar 31;2(4):185–99. doi: 10.18632/aging.100134 (PMC2881509; doi:10.18632/aging.100134)
Supplement: Supplementary Table 4-10 [file aging-02-185-s004-10.pdf]

Supplementary Table 4. Top 100 Tumor Stromal Genes Transcriptionally Associated with Tumor Stroma vs. Normal Stroma.

Gene	Description	p-value
**Col11a1**	**collagen, type XI, alpha 1**	**1.51e-73**
Mmp11	matrix metallopeptidase 11	1.34e-66
**Gjb2**	**gap junction protein, beta 2**	**2.55e-57**
**Kif26b**	**kinesin family member 26B**	**9.92e-54**
Col8a1	collagen, type VIII, alpha 1	1.11e-51
Wisp1	WNT1 inducible signaling pathway protein 1	1.87e-48
Cxcr4	chemokine (C-X-C motif) receptor 4	4.86e-43
Col10a1	collagen, type X, alpha 1	2.37e-42
**Entpd7**	**ectonucleoside triphosphate diphosphohydrolase 7**	**4.96e-42**
Comp	cartilage oligomeric matrix protein	1.67e-41
Havcr2	hepatitis A virus cellular receptor 2	1.43e-40
Acbd3	acyl-Coenzyme A binding domain containing 3	3.09e-40
Mical2	microtubule associated monoxygenase, calponin and LIM domain containing 2	3.22e-40
Clec7a	C-type lectin domain family 7, member a	2.97e-39
Bmp8a	bone morphogenetic protein 8a	5.50e-39
Snrpd3	small nuclear ribonucleoprotein D3	1.02e-38
Plau	plasminogen activator, urokinase	3.64e-38
**Capg**	**capping protein (actin filament), gelsolin-like**	**4.18e-38**
**Slc7a6**	**solute carrier family 7 (cationic amino acid transporter, y+ system), member 6**	**4.40e-38**
Tnfsf4	tumor necrosis factor (ligand) superfamily, member 4	1.25e-37
Tuft1	tuftelin 1	1.44e-37
**Adam8**	**a disintegrin and metallopeptidase domain 8**	**3.65e-37**
Alox5ap	arachidonate 5-lipoxygenase activating protein	7.91e-37
P4ha3	procollagen-proline, 2-oxoglutarate 4-dioxygenase (proline 4-hydroxylase), alpha polypeptide III	1.65e-36
Itga11	integrin alpha 11	3.47e-36
**Ctsb**	**cathepsin B**	**4.11e-36**
Zfyve1	zinc finger, FYVE domain containing 1	5.79e-36
Ifi30	interferon gamma inducible protein 30	6.24e-36
Sparc	secreted acidic cysteine rich glycoprotein	1.49e-35
Glis3	GLIS family zinc finger 3	3.13e-35
**Corin**	**corin**	**4.58e-35**
Vdr	vitamin D receptor	6.38e-35
**Prss22**	**protease, serine, 22**	**2.28e-34**
Cd86	CD86 antigen	2.63e-34
Hint3	histidine triad nucleotide binding protein 3	2.87e-34
Msr1	macrophage scavenger receptor 1	3.25e-34
Krt35	keratin 35	5.75e-34
Col12a1	collagen, type XII, alpha 1	6.40e-34

Ly86	lymphocyte antigen 86	7.28e-34
Upk1a	uroplakin 1A	9.78e-34
Pldn	pallidin	1.08e-33
Aebp1	AE binding protein 1	1.25e-33
Tnpo2	transportin 2 (importin 3, karyopherin beta 2b)	1.27e-33
Il4i1	interleukin 4 induced 1	1.56e-33
**Sulf2**	**sulfatase 2**	**2.14e-33**
Noxo1	NADPH oxidase organizer 1	2.21e-33
**Cdh11**	**cadherin 11**	**2.50e-33**
Fgd3	FYVE, RhoGEF and PH domain containing 3	2.52e-33
Rsad2	radical S-adenosyl methionine domain containing 2	4.84e-33
Manea	mannosidase, endo-alpha	6.94e-33
Col5a2	collagen, type V, alpha 2	7.78e-33
**Coro2a**	**coronin, actin binding protein 2A**	**1.03e-32**
Abo	ABO blood group (transferase A, alpha 1-3-N-acetylgalactosaminyltransferase, transferase B, alpha 1-3-galactosyltransferase)	1.10e-32
Tmem125	transmembrane protein 125	1.79e-32
Tlr4	toll-like receptor 4	1.89e-32
Ccl11	chemokine (C-C motif) ligand 11	2.94e-32
**Arhgdib**	**Rho,GDP dissociation inhibitor (GDI) beta**	**3.92e-32**
Hs3st3a1	heparan sulfate (glucosamine) 3-O-sulfotransferase 3A1	4.91e-32
**Cdcp1**	**CUB domain containing protein 1**	**5.56e-32**
**Adam12**	**a disintegrin and metallopeptidase domain 12 (meltrin alpha)**	**9.92e-32**
Tmem51	transmembrane protein 51	1.02e-31
Uqcrq	ubiquinol-cytochrome c reductase, complex III subunit VII	1.21e-31
Asb12	ankyrin repeat and SOCS box-containing 12	1.23e-31
Igsf9	immunoglobulin superfamily, member 9	1.38e-31
Antxr1	anthrax toxin receptor 1	1.52e-31
**Gpx1**	**glutathione peroxidase 1**	**1.77e-31**
**Gpr68**	**G protein-coupled receptor 68**	**1.85e-31**
Cldn14	claudin 14	1.96e-31
Nudt3	nudix (nucleotide diphosphate linked moiety X)-type motif 3	2.03e-31
**Itgax**	**integrin alpha X**	**2.17e-31**
Tmem65	transmembrane protein 65	2.52e-31
Hmox1	heme oxygenase (decycling) 1	2.53e-31
**Col5a1**	**collagen, type V, alpha 1**	**2.54e-31**
Ids	iduronate 2-sulfatase	2.65e-31
Nox4	NADPH oxidase 4	2.75e-31
Nck2	non-catalytic region of tyrosine kinase adaptor protein 2	3.01e-31
Sulf1	sulfatase 1	3.11e-31
**Cxcl9**	**chemokine (C-X-C motif) ligand 9**	**3.30e-31**
**Sipa1l3**	**signal-induced proliferation-associated 1 like 3**	**6.65e-31**

Adar	adenosine deaminase, RNA-specific	6.99e-31
**Cmtm3**	**CKLF-like MARVEL transmembrane domain containing 3**	**7.05e-31**
Tspan32	tetraspanin 32	7.25e-31
Edn2	endothelin 2	7.99e-31
Zfp161	zinc finger protein 161	8.89e-31
Sp4	trans-acting transcription factor 4	1.11e-30
Paip1	polyadenylate binding protein-interacting protein 1	1.12e-30
Aspn	asporin	1.16e-30
**Loxl1**	**lysyl oxidase-like 1**	**1.37e-30**
Trem1	triggering receptor expressed on myeloid cells 1	1.55e-30
**Abl1**	**c-abl oncogene 1, receptor tyrosine kinase**	**1.70e-30**
Cybb	cytochrome b-245, beta polypeptide	2.18e-30
Akap3	A kinase (PRKA) anchor protein 3	3.67e-30
Lass6	LAG1 homolog, ceramide synthase 6	3.86e-30
Yipf1	Yip1 domain family, member 1	3.86e-30
Tmprss11e	transmembrane protease, serine 11e	3.90e-30
**Ddr1**	**discoidin domain receptor family, member 1**	**4.58e-30**
**Nebl**	**nebulette**	**6.14e-30**
**Zc3hav1**	**zinc finger CCCH type, antiviral 1**	**6.21e-30**
Ampd3	adenosine monophosphate deaminase 3	6.38e-30
Larp2	La ribonucleoprotein domain family, member 2	8.45e-30

Genes in **BOLD** are also transcriptionally upregulated in Cav-1 (-/-) bone marrow derived stromal cells.

Supplementary Table 5. Top 100 Tumor Stromal Genes Transcriptionally Associated with Tumor Recurrence.

Gene	Description	p-value
Lad1	ladinin	2.89e-12
Akap8	A kinase (PRKA) anchor protein 8	1.50e-09
Kif23	kinesin family member 23	4.27e-09
Nanos1	nanos homolog 1 (Drosophila)	1.17e-08
Rab3d	RAB3D, member RAS oncogene family	1.33e-08
Atp6v1a	ATPase, H+ transporting, lysosomal V1 subunit A	1.51e-08
**Cpamd8**	**C3 and PZP-like, alpha-2-macroglobulin domain containing 8**	**7.11e-08**
**Pkmyt1**	**protein kinase, membrane associated tyrosine/threonine 1**	**9.13e-08**
Kmo	kynurenine 3-monooxygenase (kynurenine 3-hydroxylase)	1.07e-07
Ptpn11	protein tyrosine phosphatase, non-receptor type 11	1.24e-07
Tmem132a	transmembrane protein 132A	2.94e-07
Steap3	STEAP family member 3	3.10e-07
**Cep290**	**centrosomal protein 290**	**5.08e-07**
Tpm1	tropomyosin 1, alpha	5.23e-07
Ppp1r1b	protein phosphatase 1, regulatory (inhibitor) subunit 1B	5.30e-07
Hrasls	HRAS-like suppressor 6.	16e-07
**Vwa1**	**von Willebrand factor A domain containing 1**	**9.87e-07**
Arl8a	ADP-ribosylation factor-like 8A 1.	18e-06
**Traf1**	**TNF receptor-associated factor 1**	**1.27e-06**
Tlr9	toll-like receptor 9	1.47e-06
Klf5	Kruppel-like factor 5	1.72e-06
**Loxl3**	**lysyl oxidase-like 3**	**1.89e-06**
**Dst**	**dystonin**	**1.92e-06**
Rnf170	ring finger protein 170	2.06e-06
Bspry	B-box and SPRY domain containing	2.30e-06
**Azi1**	**5-azacytidine induced gene 1**	**2.61e-06**
Fabp6	fatty acid binding protein 6, ileal (gastrotropin)	2.69e-06
Foxred2	FAD-dependent oxidoreductase domain containing 2	2.82e-06
Hsf1	heat shock factor 1	3.12e-06
**Erbb2**	**v-erb-b2 erythroblastic leukemia viral oncogene homolog 2, neuro/glioblastoma derived oncogene homolog (avian)**	**3.14e-06**
**Sall4**	**sal-like 4 (Drosophila)**	**3.26e-06**
Pycrl	pyrroline-5-carboxylate reductase-like	3.43e-06
Spp1	secreted phosphoprotein 1	3.48e-06
Dlx4	distal-less homeobox 4 4.	10e-06
Il4i1	interleukin 4 induced 1	4.22e-06
Apbb1	amyloid beta (A4) precursor protein-binding, family B, member 1	4.67e-06
**Tpp2**	**tripeptidyl peptidase II**	**4.81e-06**
**Cldn3**	**claudin 3**	**5.27e-06**

**Lynx1**	**Ly6/neurotoxin 1**	**5.36e-06**
Ovol1	OVO homolog-like 1 (Drosophila)	5.55e-06
Opa3	optic atrophy 3 (human)	5.55e-06
Tbc1d24	TBC1 domain family, member 24	6.12e-06
Sost	sclerostin	6.72e-06
**Limk2**	**LIM motif-containing protein kinase 2**	**6.81e-06**
Dusp2	dual specificity phosphatase 2	6.88e-06
**Ak5**	**adenylate kinase 5**	**7.04e-06**
Chrm1	cholinergic receptor, muscarinic 1, CNS	8.10e-06
**Arv1**	**ARV1 homolog (yeast)**	**9.10e-06**
Dhx32	DEAH (Asp-Glu-Ala-His) box polypeptide 32	9.74e-06
**Slc23a2**	**solute carrier family 23 (nucleobase transporters), member 2**	**1.01e-05**
Tspan32	tetraspanin 32	1.11e-05
Hoxb13	homeo box B13	1.13e-05
Chd7	chromodomain helicase DNA binding protein 7	1.21e-05
Pld4	phospholipase D family, member 4	1.21e-05
**Hk2**	**hexokinase 2**	**1.23e-05**
Eif4ebp1	eukaryotic translation initiation factor 4E binding protein 1	1.41e-05
**Ncoa5**	**nuclear receptor coactivator 5**	**1.45e-05**
**Dpysl2**	**dihydropyrimidinase-like 2**	**1.50e-05**
Kdelr2	KDEL (Lys-Asp-Glu-Leu) endoplasmic reticulum protein retention receptor 2	1.56e-05
**Susd2**	**sushi domain containing 2**	**1.56e-05**
Dusp3	dual specificity phosphatase 3 (vaccinia virus phosphatase VH1-related)	1.61e-05
Zc3h12a	zinc finger CCCH type containing 12A	1.66e-05
Tpm4	tropomyosin 4	1.68e-05
**Prss22**	**protease, serine, 22**	**1.70e-05**
Sh3bp2	SH3-domain binding protein 2	1.73e-05
Cldn9	claudin 9	1.93e-05
Cxcl16	chemokine (C-X-C motif) ligand 16	2.01e-05
Pfkfb3	6-phosphofructo-2-kinase/fructose-2,6-biphosphatase 3	2.02e-05
Rims2	regulating synaptic membrane exocytosis 2	2.02e-05
**Eppk1 ---**		**2.03e-05**
Tst	thiosulfate sulfurtransferase, mitochondrial	2.06e-05
**Fam65a**	**family with sequence similarity 65, member A**	**2.13e-05**
**Pla2g3**	**phospholipase A2, group III**	**2.13e-05**
Siglec5	sialic acid binding Ig-like lectin 5	2.19e-05
Bpil2	bactericidal/permeability-increasing protein-like 2	2.24e-05
Fgf2	fibroblast growth factor 2	2.26e-05
**Sox21**	**SRY-box containing gene 21**	**2.43e-05**
Eda	ectodysplasin-A	2.50e-05
Snf8	SNF8, ESCRT-II complex subunit, homolog (S. cerevisiae)	2.55e-05

Yipf2	Yip1 domain family, member 2	2.55e-05
Wdr51a	WD repeat domain 51A	2.67e-05
Ak3l1	adenylate kinase 3-like 1	2.85e-05
**Vps18**	**vacuolar protein sorting 18 (yeast)**	**2.86e-05**
Mark2	MAP/microtubule affinity-regulating kinase 2	2.88e-05
**Cdh12**	**cadherin 12**	**2.89e-05**
Cabp4	calcium binding protein 4	2.91e-05
Pcgf1	polycomb group ring finger 1	2.94e-05
**Cacnb1**	**calcium channel, voltage-dependent, beta 1 subunit**	**3.07e-05**
Acy3	aspartoacylase (aminoacylase) 3	3.15e-05
Crlf3	cytokine receptor-like factor 3	3.17e-05
Slc39a4	solute carrier family 39 (zinc transporter), member 4	3.20e-05
**Ctnna2**	**catenin (cadherin associated protein), alpha 2**	**3.33e-05**
Ncald	neurocalcin delta	3.41e-05
Kcnv2	potassium channel, subfamily V, member 2	3.41e-05
Letm1	leucine zipper-EF-hand containing transmembrane protein 1	3.56e-05
Csdc2	cold shock domain containing C2, RNA binding	3.63e-05
Arfip2	ADP-ribosylation factor interacting protein 2	3.68e-05
**Gp1ba**	**glycoprotein 1b, alpha polypeptide**	**3.69e-05**
**Gcn1l1**	**GCN1 general control of amino-acid synthesis 1-like 1 (yeast)**	**3.73e-05**
**Ppp1r13l**	**protein phosphatase 1, regulatory (inhibitor) subunit 13 like**	**3.88e-05**

Genes in **BOLD** are also transcriptionally upregulated in Cav-1 (-/-) bone marrow derived stromal cells.

Supplementary Table 6. Top 100 Tumor Stromal Genes Transcriptionally Associated with LN Metastais.

Gene	Description	p-value
**Gcn1l1**	**GCN1 general control of amino-acid synthesis 1-like 1 (yeast)**	**7.07e-07**
Alkbh2	alkB, alkylation repair homolog 2 (E. coli)	8.42e-07
Dhrs2	dehydrogenase/reductase member 2	4.06e-06
**Pycr2**	**pyrroline-5-carboxylate reductase family, member 2**	**3.31e-05**
**Pex14**	**peroxisomal biogenesis factor 14**	**5.67e-05**
Ptdss2	phosphatidylserine synthase 2	6.99e-05
**Tsc2**	**tuberous sclerosis 2**	**9.19e-05**
Efna1	ephrin A1	9.29e-05
**Zfpm1**	**zinc finger protein, multitype 1**	**1.52e-04**
**Pold3**	**polymerase (DNA-directed), delta 3, accessory subunit**	**1.64e-04**
Med28	mediator of RNA polymerase II transcription, subunit 28 homolog (yeast)	1.93e-04
**Mki67ip**	**Mki67 (FHA domain) interacting nucleolar phosphoprotein**	**2.15e-04**
**Sox1**	**SRY-box containing gene 1**	**2.26e-04**
**Myb**	**myeloblastosis oncogene**	**2.86e-04**
Rpl21	ribosomal protein L21	3.16e-04
**Fbxo41**	**F-box protein 41**	**3.20e-04**
Gpr153	G protein-coupled receptor 153	3.58e-04
**Upf1**	**UPF1 regulator of nonsense transcripts homolog (yeast)**	**3.60e-04**
Diras1	DIRAS family, GTP-binding RAS-like 1	3.83e-04
Gdf15	growth differentiation factor 15	3.94e-04
Snrpe ---		4.20e-04
Wdr5b	WD repeat domain 5B	4.30e-04
Vtn	vitronectin	4.52e-04
**Scrib**	**scribbled homolog (Drosophila)**	**4.64e-04**
**Acss1**	**acyl-CoA synthetase short-chain family member 1**	**5.24e-04**
Sox8	SRY-box containing gene 8	5.28e-04
**Ddx54**	**DEAD (Asp-Glu-Ala-Asp) box polypeptide 54**	**5.83e-04**
**Hgs**	**HGF-regulated tyrosine kinase substrate**	**5.98e-04**
Ndufb7	NADH dehydrogenase (ubiquinone) 1 beta subcomplex, 7	6.03e-04
Bax	BCL2-associated X protein	6.20e-04
**Kiss1r**	**KISS1 receptor**	**6.33e-04**
**Syne2**	**synaptic nuclear envelope 2**	**6.51e-04**
**Rfng**	**RFNG O-fucosylpeptide 3-beta-N-acetylglucosaminyltransferase**	**6.61e-04**
**Rnaseh2a**	**ribonuclease H2, large subunit**	**6.83e-04**
Snupn	snurportin 1	6.92e-04
**Ppp1r16a**	**protein phosphatase 1, regulatory (inhibitor) subunit 16A**	**7.67e-04**
**Spata4**	**spermatogenesis associated 4**	**8.30e-04**
Pdcd6ip	programmed cell death 6 interacting protein	8.35e-04

Ndufa3	NADH dehydrogenase (ubiquinone) 1 alpha subcomplex, 3	8.55e-04
Trub2	TruB pseudouridine (psi) synthase homolog 2 (E. coli)	8.56e-04
Hoxb3	homeo box B3	8.67e-04
Tmem121	transmembrane protein 121	9.18e-04
Hspb1 ---		9.27e-04
**Pld3**	**phospholipase D family, member 3**	**9.75e-04**
Trmt1	TRM1 tRNA methyltransferase 1 homolog (S. cerevisiae)	9.83e-04
Sall2	sal-like 2 (Drosophila)	9.97e-04
Scarb1	scavenger receptor class B, member 1	1.03e-03
Cebpa	CCAAT/enhancer binding protein (C/EBP), alpha	1.06e-03
Hdgf	hepatoma-derived growth factor	1.09e-03
Bcat2	branched chain aminotransferase 2, mitochondrial	1.10e-03
Caskin2	CASK-interacting protein 2	1.12e-03
Igfbp2	insulin-like growth factor binding protein 2	1.12e-03
Cpsf6	cleavage and polyadenylation specific factor 6	1.15e-03
**Tbcd**	**tubulin-specific chaperone d**	**1.15e-03**
**Lamb3**	**laminin, beta 3**	**1.18e-03**
**Itfg3**	**integrin alpha FG-GAP repeat containing 3**	**1.18e-03**
Ing5	inhibitor of growth family, member 5	1.19e-03
**Acad10**	**acyl-Coenzyme A dehydrogenase family, member 10**	**1.29e-03**
Eno3	enolase 3, beta muscle	1.29e-03
Jrk	jerky	1.34e-03
Apod	apolipoprotein D	1.37e-03
Igsf9	immunoglobulin superfamily, member 9	1.41e-03
Phf10	PHD finger protein 10	1.42e-03
Aldh16a1	aldehyde dehydrogenase 16 family, member A1	1.49e-03
Eif4b	eukaryotic translation initiation factor 4B	1.52e-03
Las1l	LAS1-like (S. cerevisiae)	1.67e-03
**Zmat2**	**zinc finger, matrin type 2**	**1.69e-03**
Aldoa	aldolase A, fructose-bisphosphate	1.69e-03
**Grin2d**	**glutamate receptor, ionotropic, NMDA2D (epsilon 4)**	**1.78e-03**
Emp2	epithelial membrane protein 2	1.80e-03
Fbxo34	F-box protein 34	1.86e-03
Ccdc104	coiled-coil domain containing 104	1.86e-03
Cyp4b1	cytochrome P450, family 4, subfamily b, polypeptide 1	1.89e-03
Deaf1	deformed epidermal autoregulatory factor 1 (Drosophila)	1.96e-03
Sema6b	sema domain, transmembrane domain (TM), and cytoplasmic domain, (semaphorin) 6B	1.98e-03
Nkd2	naked cuticle 2 homolog (Drosophila)	2.02e-03
**Klf16**	**Kruppel-like factor 16**	**2.02e-03**
Gabrd	gamma-aminobutyric acid (GABA) A receptor, subunit delta	2.02e-03
Mrpl9	mitochondrial ribosomal protein L9	2.03e-03

Csnk1e	casein kinase 1, epsilon	2.04e-03
Cited4	Cbp/p300-interacting transactivator, with Glu/Asp-rich carboxy-terminal domain, 4	2.04e-03
Cdc42se2	CDC42 small effector 2	2.04e-03
Hoxa3	homeo box A3	2.13e-03
**Tnnc2**	**troponin C2, fast**	**2.15e-03**
Hoxb13	homeo box B13	2.18e-03
**Cst6**	**cystatin E/M**	**2.18e-03**
Sfi1	Sfi1 homolog, spindle assembly associated (yeast)	2.27e-03
Cope	coatomer protein complex, subunit epsilon	2.30e-03
**Phox2a**	**paired-like homeobox 2a**	**2.33e-03**
Tff3	trefoil factor 3, intestinal	2.38e-03
**Pcnt**	**pericentrin (kendrin)**	**2.39e-03**
Mpst	mercaptopyruvate sulfurtransferase	2.39e-03
Nr2f1	nuclear receptor subfamily 2, group F, member 1	2.41e-03
Pnliprp1	pancreatic lipase related protein 1	2.48e-03
Prodh	proline dehydrogenase	2.60e-03
Tcf7	transcription factor 7, T-cell specific	2.63e-03
Trpv1	transient receptor potential cation channel, subfamily V, member 1	2.65e-03
Lrrc14	leucine rich repeat containing 14	2.70e-03
Gamt	guanidinoacetate methyltransferase	2.73e-03
Antxr2	anthrax toxin receptor 2	2.85e-03

Genes in **BOLD** are also transcriptionally upregulated in Cav-1 (-/-) bone marrow derived stromal cells.

Supplementary Table 7.
Intersection of the Tumor vs. Normal Stromal Gene Set with Other Gene Sets.

Glycolysis (19 genes)

Acss1	acyl-CoA synthetase short-chain family member 1
Adh5	alcohol dehydrogenase 5 (class III), chi polypeptide
Aldh1a3	aldehyde dehydrogenase family 1, subfamily A3
Aldh1b1	aldehyde dehydrogenase 1 family, member B1
Aldh2	aldehyde dehydrogenase 2, mitochondrial
Aldh3b1	aldehyde dehydrogenase 3 family, member B1
Aldh9a1	aldehyde dehydrogenase 9, subfamily A1
Aldob	aldolase B, fructose-bisphosphate
Bpgm	2,3-bisphosphoglycerate mutase
Dlat	dihydrolipoamide S-acetyltransferase (E2 component of pyruvate dehydrogenase complex)
Fbp1	fructose bisphosphatase 1
Fbp2	fructose bisphosphatase 2
G6pc	glucose-6-phosphatase, catalytic
Hk2	hexokinase 2
Ldhal6b	lactate dehydrogenase A-like 6B
Ldhc	lactate dehydrogenase C
Pdha2	pyruvate dehydrogenase E1 alpha 2
Pgam2	phosphoglycerate mutase 2
Pgm2	phosphoglucomutase 2

HIF Target Genes (213 genes)

Abcf2	ATP-binding cassette, sub-family F (GCN20), member 2
Acbd3	acyl-Coenzyme A binding domain containing 3
Acbd5	acyl-Coenzyme A binding domain containing 5
Actr1a	ARP1 actin-related protein 1 homolog A, centractin alpha (yeast)
Adat1	adenosine deaminase, tRNA-specific 1
Adra1b	adrenergic receptor, alpha 1b
Aff1	AF4/FMR2 family, member 1
Aggf1	angiogenic factor with G patch and FHA domains 1
Akap12	A kinase (PRKA) anchor protein (gravin) 12
Aldh4a1	aldehyde dehydrogenase 4 family, member A1
Angptl6	angiopoietin-like 6
Ankrd37	ankyrin repeat domain 37
Arf4	ADP-ribosylation factor 4
Arhgap25	Rho GTPase activating protein 25
Arhgef1	Rho guanine nucleotide exchange factor (GEF) 1
Arid1a	AT rich interactive domain 1A (SWI-like)
Arl5b	ADP-ribosylation factor-like 5B
Asph	aspartate-beta-hydroxylase
Atf3	activating transcription factor 3
Atf7	activating transcription factor 7
Atf7ip	activating transcription factor 7 interacting protein
Bbx	bobby sox homolog (Drosophila)
Bcl11a	B-cell CLL/lymphoma 11A (zinc finger protein)
Bcl2l11	BCL2-like 11 (apoptosis facilitator)
Bcl9l	B-cell CLL/lymphoma 9-like
Bnip3l	BCL2/adenovirus E1B interacting protein 3-like
Calu	calumenin
Cast	calpastatin
Ccnb1	cyclin B1
Cdc42ep4	CDC42 effector protein (Rho GTPase binding) 4

Cdkn1a	cyclin-dependent kinase inhibitor 1A (P21)
Chac1	ChaC, cation transport regulator-like 1 (E. coli)
Col5a1	collagen, type V, alpha 1
Copz1	coatomer protein complex, subunit zeta 1
Coq10b	coenzyme Q10 homolog B (S. cerevisiae)
Crh	corticotropin releasing hormone
Crispld1	cysteine-rich secretory protein LCCL domain containing 1
Crkl	v-crk sarcoma virus CT10 oncogene homolog (avian)-like
Cxcl12	chemokine (C-X-C motif) ligand 12
Cxcr4	chemokine (C-X-C motif) receptor 4
Cyp2s1	cytochrome P450, family 2, subfamily s, polypeptide 1
Cyr61	cysteine rich protein 61
Dbf4	DBF4 homolog (S. cerevisiae)
Dnajc10	DnaJ (Hsp40) homolog, subfamily C, member 10
Dnajc11	DnaJ (Hsp40) homolog, subfamily C, member 11
Dpysl2	dihydropyrimidinase-like 2
Edem1	ER degradation enhancer, mannosidase alpha-like 1
Edn1	endothelin 1
Eed	embryonic ectoderm development
Eif1	eukaryotic translation initiation factor 1
Eif4enif1	eukaryotic translation initiation factor 4E nuclear import factor 1
Elavl1	ELAV (embryonic lethal, abnormal vision, Drosophila)-like 1 (Hu antigen R)
Ell2	elongation factor RNA polymerase II 2
Elmo1	engulfment and cell motility 1, ced-12 homolog (C. elegans)
Ero1l	ERO1-like (S. cerevisiae)
Ets1	E26 avian leukemia oncogene 1, 5' domain
Evi1	ecotropic viral integration site 1
Ewsr1	Ewing sarcoma breakpoint region 1
Fam76a	family with sequence similarity 76, member A
Fam98a	family with sequence similarity 98, member A
Fbxo42	F-box protein 42
Fbxw7	F-box and WD-40 domain protein 7
Fgd6	FYVE, RhoGEF and PH domain containing 6
Fgf11	fibroblast growth factor 11
Fgfr1op2	FGFR1 oncogene partner 2
Fgr	Gardner-Rasheed feline sarcoma viral (Fgr) oncogene homolog
Fkbp7	FK506 binding protein 7
Fn1	fibronectin 1
Fndc3b	fibronectin type III domain containing 3B
Fosl2	fos-like antigen 2
Fsd1l	fibronectin type III and SPRY domain containing 1-like
Furin	furin (paired basic amino acid cleaving enzyme)
Gad1	glutamic acid decarboxylase 1
Gadd45b	growth arrest and DNA-damage-inducible 45 beta
Ganab	alpha glucosidase 2 alpha neutral subunit
Gatad2b	GATA zinc finger domain containing 2B
Gmppb	GDP-mannose pyrophosphorylase B
Golga1	golgi autoantigen, golgin subfamily a, 1
Gopc	golgi associated PDZ and coiled-coil motif containing
Gosr2	golgi SNAP receptor complex member 2
Grk6	G protein-coupled receptor kinase 6
Hcfc1r1	host cell factor C1 regulator 1 (XPO1-dependent)
Hes1	hairy and enhancer of split 1 (Drosophila)
Hic2	hypermethylated in cancer 2
Hk2	hexokinase 2
Hmox1	heme oxygenase (decycling) 1

Hsp90b1	heat shock protein 90, beta (Grp94), member 1
Hyou1	hypoxia up-regulated 1
Ifi30	interferon gamma inducible protein 30
Igf2	insulin-like growth factor 2
Inha	inhibin alpha
Irf1	interferon regulatory factor 1
Itgb2	integrin beta 2
Ivns1abp	influenza virus NS1A binding protein
Klhdc1	kelch domain containing 1
Klhl24	kelch-like 24 (Drosophila)
Kpna4	karyopherin (importin) alpha 4
Krt18	keratin 18
Krt19	keratin 19
Lcorl	ligand dependent nuclear receptor corepressor-like
Lrrc20	leucine rich repeat containing 20
Lsp1	lymphocyte specific 1
Luc7l2	LUC7-like 2 (S. cerevisiae)
Lysmd3	LysM, putative peptidoglycan-binding, domain containing 3
Map3k14	mitogen-activated protein kinase kinase kinase 14
Mcl1	myeloid cell leukemia sequence 1
Mecp2	methyl CpG binding protein 2
Met	met proto-oncogene
Mfsd2	major facilitator superfamily domain containing 2
Mga	MAX gene associated
Mgat2	mannoside acetylglucosaminyltransferase 2
Mknk1	MAP kinase-interacting serine/threonine kinase 1
Mlxip	MLX interacting protein
Mrps12	mitochondrial ribosomal protein S12
Mxd1	MAX dimerization protein 1
Mylk	myosin, light polypeptide kinase
Ndrg1	N-myc downstream regulated gene 1
Nek6	NIMA (never in mitosis gene a)-related expressed kinase 6
Nln	neurolysin (metallopeptidase M3 family)
Nos3	nitric oxide synthase 3, endothelial cell
Nr4a1	nuclear receptor subfamily 4, group A, member 1
Nrn1	neuritin 1
Ntrk2	neurotrophic tyrosine kinase, receptor, type 2
Nup98	nucleoporin 98
Oxsr1	oxidative-stress responsive 1
P4ha2	procollagen-proline, 2-oxoglutarate 4-dioxygenase (proline 4-hydroxylase), alpha II polypeptide
P4hb	prolyl 4-hydroxylase, beta polypeptide
Pa2g4	proliferation-associated 2G4
Paip1	polyadenylate binding protein-interacting protein 1
Pak2	p21 (CDKN1A)-activated kinase 2
Papss2	3'-phosphoadenosine 5'-phosphosulfate synthase 2
Pdgfa	platelet derived growth factor, alpha
Pdk4	pyruvate dehydrogenase kinase, isoenzyme 4
Pdlim5	PDZ and LIM domain 5
Per2	period homolog 2 (Drosophila)
Pfkfb3	6-phosphofructo-2-kinase/fructose-2,6-biphosphatase 3
Pgm3	phosphoglucomutase 3
Phf12	PHD finger protein 12
Phospho1	phosphatase, orphan 1
Piga	phosphatidylinositol glycan anchor biosynthesis, class A
Pik3r3	phosphatidylinositol 3 kinase, regulatory subunit, polypeptide 3 (p55)

Pim1	proviral integration site 1
Pitpna	phosphatidylinositol transfer protein, alpha
Plekhm1	pleckstrin homology domain containing, family M (with RUN domain) member 1
Plod2	procollagen lysine, 2-oxoglutarate 5-dioxygenase 2
Pnma2	paraneoplastic antigen MA2
Ppapdc2	phosphatidic acid phosphatase type 2 domain containing 2
Ppm1b	protein phosphatase 1B, magnesium dependent, beta isoform
Ppme1	protein phosphatase methylesterase 1
Ppp1r10	protein phosphatase 1, regulatory subunit 10
Ppp1r13b	protein phosphatase 1, regulatory (inhibitor) subunit 13B
Ppp1r13l	protein phosphatase 1, regulatory (inhibitor) subunit 13 like
Ppp1r3c	protein phosphatase 1, regulatory (inhibitor) subunit 3C
Psd3	pleckstrin and Sec7 domain containing 3
Psip1	PC4 and SFRS1 interacting protein 1
Psme3	proteaseome (prosome, macropain) 28 subunit, 3
Ptplad1	protein tyrosine phosphatase-like A domain containing 1
Ptpn12	protein tyrosine phosphatase, non-receptor type 12
Pxn	paxillin
Rab10	RAB10, member RAS oncogene family
Rab8b	RAB8B, member RAS oncogene family
Ralgps1	Ral GEF with PH domain and SH3 binding motif 1
Rara	retinoic acid receptor, alpha
Rassf2	Ras association (RalGDS/AF-6) domain family member 2
Rassf4	Ras association (RalGDS/AF-6) domain family member 4
Rgs1	regulator of G-protein signaling 1
Rsu1	Ras suppressor protein 1
Rybp	RING1 and YY1 binding protein
Sdccag8	serologically defined colon cancer antigen 8
Sdk1	sidekick homolog 1 (chicken)
Sec24a	Sec24 related gene family, member A (S. cerevisiae)
Serp1	stress-associated endoplasmic reticulum protein 1
Sertad1	SERTA domain containing 1
Sfxn3	sideroflexin 3
Slc16a1	solute carrier family 16 (monocarboxylic acid transporters), member 1
Slc25a28	solute carrier family 25, member 28
Slc35e1	solute carrier family 35, member E1
Slc6a6	solute carrier family 6 (neurotransmitter transporter, taurine), member 6
Slc7a6	solute carrier family 7 (cationic amino acid transporter, y+ system), member 6
Snca	synuclein, alpha
Snd1	staphylococcal nuclease and tudor domain containing 1
Sox4	SRY-box containing gene 4
Spast	spastin
Srrm2	serine/arginine repetitive matrix 2
Stard3nl	STARD3 N-terminal like
Stard4	StAR-related lipid transfer (START) domain containing 4
Stat3	signal transducer and activator of transcription 3
Stk17b	serine/threonine kinase 17b (apoptosis-inducing)
Stx16	syntaxin 16
Syt17	synaptotagmin XVII
Tert	telomerase reverse transcriptase
Tff3	trefoil factor 3, intestinal
Tgfb3	transforming growth factor, beta 3
Tgfbr1	transforming growth factor, beta receptor I
Tgm2	transglutaminase 2, C polypeptide
Tiparp	TCDD-inducible poly(ADP-ribose) polymerase
Tmcc3	transmembrane and coiled coil domains 3

Tmem39a	transmembrane protein 39a
Tmpo	thymopoietin
Tpcn1	two pore channel 1
Trim2	tripartite motif-containing 2
Trim9	tripartite motif-containing 9
Txnip	thioredoxin interacting protein
Ube2q1	ubiquitin-conjugating enzyme E2Q (putative) 1
Ubqln1	ubiquilin 1
Ubtf	upstream binding transcription factor, RNA polymerase I
Vcp	---
Wdr33	WD repeat domain 33
Wsb1	WD repeat and SOCS box-containing 1
Wwox	WW domain-containing oxidoreductase
Wwp2	WW domain containing E3 ubiquitin protein ligase 2
Zdhhc9	zinc finger, DHHC domain containing 9
Zfp161	zinc finger protein 161

Mitochondrial Associated Genes (233 genes)

Abat	4-aminobutyrate aminotransferase
Abcf2	ATP-binding cassette, sub-family F (GCN20), member 2
Acad9	acyl-Coenzyme A dehydrogenase family, member 9
Acadsb	acyl-Coenzyme A dehydrogenase, short/branched chain
Aco2	aconitase 2, mitochondrial
Acot2	acyl-CoA thioesterase 2
Acox1	acyl-Coenzyme A oxidase 1, palmitoyl
Acsl1	acyl-CoA synthetase long-chain family member 1
Acsl6	acyl-CoA synthetase long-chain family member 6
Acsm1	acyl-CoA synthetase medium-chain family member 1
Acsm2	acyl-CoA synthetase medium-chain family member 2
Adc	arginine decarboxylase
Akap1	A kinase (PRKA) anchor protein 1
Aldh2	aldehyde dehydrogenase 2, mitochondrial
Aldh4a1	aldehyde dehydrogenase 4 family, member A1
Aldh5a1	aldhehyde dehydrogenase family 5, subfamily A1
Als2	amyotrophic lateral sclerosis 2 (juvenile) homolog (human)
Angptl7	angiopoietin-like 7
Apex2	apurinic/apyrimidinic endonuclease 2
Apoa4	apolipoprotein A-IV
Aptx	aprataxin
Arg2	arginase type II
Arnt	aryl hydrocarbon receptor nuclear translocator
Asah2	N-acylsphingosine amidohydrolase 2
Asz1	ankyrin repeat, SAM and basic leucine zipper domain containing 1
Atox1	ATX1 (antioxidant protein 1) homolog 1 (yeast)
Atp5d	ATP synthase, H+ transporting, mitochondrial F1 complex, delta subunit
Atp5e	ATP synthase, H+ transporting, mitochondrial F1 complex, epsilon subunit
Atp5g3	ATP synthase, H+ transporting, mitochondrial F0 complex, subunit c (subunit 9), isoform 3
Atp5j	ATP synthase, H+ transporting, mitochondrial F0 complex, subunit F
Bad	BCL2-associated agonist of cell death
Bax	BCL2-associated X protein
Bbc3	BCL2 binding component 3
Bckdha	branched chain ketoacid dehydrogenase E1, alpha polypeptide
Bckdhb	branched chain ketoacid dehydrogenase E1, beta polypeptide
Bcl2	B-cell leukemia/lymphoma 2
Bcl2l1	BCL2-like 1

Bcl2l10	Bcl2-like 10
Bnip3l	BCL2/adenovirus E1B interacting protein 3-like
Bphl	biphenyl hydrolase-like (serine hydrolase, breast epithelial mucin-associated antigen)
Bzrap1	benzodiazapine receptor associated protein 1
Casp3	caspase 3
Casp7	caspase 7
Casp8	caspase 8
Casq1	calsequestrin 1
Cbx6	chromobox homolog 6
Ccdc39	coiled-coil domain containing 39
Ccl5	chemokine (C-C motif) ligand 5
Cdkn2a	cyclin-dependent kinase inhibitor 2A
Cds2	CDP-diacylglycerol synthase (phosphatidate cytidylyltransferase) 2
Cebpa	CCAAT/enhancer binding protein (C/EBP), alpha
Chdh	choline dehydrogenase
Cidea	cell death-inducing DNA fragmentation factor, alpha subunit-like effector A
Ckmt2	creatine kinase, mitochondrial 2
Clic4	chloride intracellular channel 4 (mitochondrial)
Cln3	ceroid lipofuscinosis, neuronal 3, juvenile (Batten, Spielmeyer-Vogt disease)
Cln8	ceroid-lipofuscinosis, neuronal 8
Clybl	citrate lyase beta like
Coq2	coenzyme Q2 homolog, prenyltransferase (yeast)
Coq9	coenzyme Q9 homolog (yeast)
Cox11	COX11 homolog, cytochrome c oxidase assembly protein (yeast)
Cox18	---
Cox4nb	COX4 neighbor
Cox6c	cytochrome c oxidase, subunit VIc
Cpt1b	carnitine palmitoyltransferase 1b, muscle
Cpt1c	carnitine palmitoyltransferase 1c
Creb1	cAMP responsive element binding protein 1
Cryaa	crystallin, alpha A
Cryab	crystallin, alpha B
Cygb	cytoglobin
Dbt	dihydrolipoamide branched chain transacylase E2
Ddx28	DEAD (Asp-Glu-Ala-Asp) box polypeptide 28
Dhx32	DEAH (Asp-Glu-Ala-His) box polypeptide 32
Diablo	diablo homolog (Drosophila)
Dmgdh	dimethylglycine dehydrogenase precursor
Dnaja3	DnaJ (Hsp40) homolog, subfamily A, member 3
Dnajc19	DnaJ (Hsp40) homolog, subfamily C, member 19
Dusp1	dual specificity phosphatase 1
Dut	deoxyuridine triphosphatase
Ech1	enoyl coenzyme A hydratase 1, peroxisomal
Efhd1	EF hand domain containing 1
Elk3	ELK3, member of ETS oncogene family
Eomes	eomesodermin homolog (Xenopus laevis)
Epas1	endothelial PAS domain protein 1
Ercc2	excision repair cross-complementing rodent repair deficiency, complementation group 2
Esr2	estrogen receptor 2 (beta)
Ethe1	ethylmalonic encephalopathy 1
Foxred1	FAD-dependent oxidoreductase domain containing 1
Fpgs	folylpolyglutamyl synthetase
Frmd6	FERM domain containing 6
Ftmt	ferritin mitochondrial

Fxn	frataxin
Fyn	Fyn proto-oncogene
Gab1	growth factor receptor bound protein 2-associated protein 1
Gatm	glycine amidinotransferase (L-arginine:glycine amidinotransferase)
Gclc	glutamate-cysteine ligase, catalytic subunit
Ghitm	growth hormone inducible transmembrane protein
Ghr	growth hormone receptor
Glrx5	glutaredoxin 5 homolog (S. cerevisiae)
Glyat	glycine-N-acyltransferase
Gpx1	glutathione peroxidase 1
Gpx2	glutathione peroxidase 2
Gpx5	glutathione peroxidase 5
Gpx6	glutathione peroxidase 6
Gpx7	glutathione peroxidase 7
Grn	granulin
Hcls1	hematopoietic cell specific Lyn substrate 1
Hebp1	heme binding protein 1
Herc2	hect (homologous to the E6-AP (UBE3A) carboxyl terminus) domain and RCC1 (CHC1)-like domain (RLD) 2
Hif1a	hypoxia inducible factor 1, alpha subunit
Hkdc1	hexokinase domain containing 1
Hmgcs2	3-hydroxy-3-methylglutaryl-Coenzyme A synthase 2
Hmox1	heme oxygenase (decycling) 1
Hmox2	heme oxygenase (decycling) 2
Hsd3b1	hydroxy-delta-5-steroid dehydrogenase, 3 beta- and steroid delta-isomerase 1
Hsd3b2	hydroxy-delta-5-steroid dehydrogenase, 3 beta- and steroid delta-isomerase 2
Hsdl2	hydroxysteroid dehydrogenase like 2
Hsh2d	hematopoietic SH2 domain containing
Idh3b	isocitrate dehydrogenase 3 (NAD+) beta
Immt	inner membrane protein, mitochondrial
Jun	Jun oncogene
Kif1b	kinesin family member 1B
Kmo	kynurenine 3-monooxygenase (kynurenine 3-hydroxylase)
Ldhd	lactate dehydrogenase D
Letm1	leucine zipper-EF-hand containing transmembrane protein 1
Letm2	leucine zipper-EF-hand containing transmembrane protein 2
Lipg	lipase, endothelial
Lyn	Yamaguchi sarcoma viral (v-yes-1) oncogene homolog
Maob	monoamine oxidase B
Mcl1	myeloid cell leukemia sequence 1
Me3	malic enzyme 3, NADP(+)-dependent, mitochondrial
Mecp2	methyl CpG binding protein 2
Mfn1	mitofusin 1
Mfn2	mitofusin 2
Mlxip	MLX interacting protein
Mmab	methylmalonic aciduria (cobalamin deficiency) type B homolog (human)
Mobp	myelin-associated oligodendrocytic basic protein
Mpo	myeloperoxidase
Mrps10	mitochondrial ribosomal protein S10
Mrps12	mitochondrial ribosomal protein S12
Mrps24	mitochondrial ribosomal protein S24
Msra	methionine sulfoxide reductase A
Msrb2	methionine sulfoxide reductase B2
Msrb3	methionine sulfoxide reductase B3
Mtch1	mitochondrial carrier homolog 1 (C. elegans)
Mtch2	mitochondrial carrier homolog 2 (C. elegans)

Mtf1	metal response element binding transcription factor 1
Muc20	mucin 20
Mut	methylmalonyl-Coenzyme A mutase
Mutyh	mutY homolog (E. coli)
Myc	myelocytomatosis oncogene
Myh7	myosin, heavy polypeptide 7, cardiac muscle, beta
Nags	N-acetylglutamate synthase
Napg	N-ethylmaleimide sensitive fusion protein attachment protein gamma
Ndufa1	NADH dehydrogenase (ubiquinone) 1 alpha subcomplex, 1
Ndufa2	NADH dehydrogenase (ubiquinone) 1 alpha subcomplex, 2
Neil1	nei endonuclease VIII-like 1 (E. coli)
Nek9	NIMA (never in mitosis gene a)-related expressed kinase 9
Nfkb1	nuclear factor of kappa light polypeptide gene enhancer in B-cells 1, p105
Nos1	nitric oxide synthase 1, neuronal
Nt5m	5',3'-nucleotidase, mitochondrial
Ogdh	oxoglutarate dehydrogenase (lipoamide)
Ogg1	8-oxoguanine DNA-glycosylase 1
Olr1	oxidized low density lipoprotein (lectin-like) receptor 1
Opa3	optic atrophy 3 (human)
Otc	ornithine transcarbamylase
Oxr1	oxidation resistance 1
Oxsr1	oxidative-stress responsive 1
P2rx7	purinergic receptor P2X, ligand-gated ion channel, 7
Pak7	p21 (CDKN1A)-activated kinase 7
Park7	Parkinson disease (autosomal recessive, early onset) 7
Parl	presenilin associated, rhomboid-like
Pdk2	pyruvate dehydrogenase kinase, isoenzyme 2
Pdk4	pyruvate dehydrogenase kinase, isoenzyme 4
Pemt	phosphatidylethanolamine N-methyltransferase
Perp	PERP, TP53 apoptosis effector
Pgs1	phosphatidylglycerophosphate synthase 1
Pink1	PTEN induced putative kinase 1
Pnkd	paroxysmal nonkinesiogenic dyskinesia
Pnkp	polynucleotide kinase 3'- phosphatase
Polg	polymerase (DNA directed), gamma
Polrmt	polymerase (RNA) mitochondrial (DNA directed)
Ppif	peptidylprolyl isomerase F (cyclophilin F)
Ppm1k	protein phosphatase 1K (PP2C domain containing)
Ppox	protoporphyrinogen oxidase
Prdx3	peroxiredoxin 3
Prep	prolyl endopeptidase
Psen1	presenilin 1
Pten	phosphatase and tensin homolog
Ptrf	polymerase I and transcript release factor
Rab32	RAB32, member RAS oncogene family
Rai14	retinoic acid induced 14
Reep1	receptor accessory protein 1
Rhot1	ras homolog gene family, member T1
Rnh1	ribonuclease/angiogenin inhibitor 1
Sardh	sarcosine dehydrogenase
Sarm1	sterile alpha and HEAT/Armadillo motif containing 1
Scara3	scavenger receptor class A, member 3
Sdc1	syndecan 1
Sfxn3	sideroflexin 3
Sfxn4	sideroflexin 4
Sfxn5	sideroflexin 5

Sgk2	serum/glucocorticoid regulated kinase 2
Shc1	src homology 2 domain-containing transforming protein C1
Sirt5	sirtuin 5 (silent mating type information regulation 2 homolog) 5 (S. cerevisiae)
Slc25a12	solute carrier family 25 (mitochondrial carrier, Aralar), member 12
Slc25a14	solute carrier family 25 (mitochondrial carrier, brain), member 14
Slc25a22	solute carrier family 25 (mitochondrial carrier, glutamate), member 22
Slc9a6	solute carrier family 9 (sodium/hydrogen exchanger), member 6
Smcp	sperm mitochondria-associated cysteine-rich protein
Smcr7l	Smith-Magenis syndrome chromosome region, candidate 7-like (human)
Sod2	superoxide dismutase 2, mitochondrial
Spg7	spastic paraplegia 7 homolog (human)
Stard3	START domain containing 3
Stat1	signal transducer and activator of transcription 1
Syk	spleen tyrosine kinase
Synj2	synaptojanin 2
Tdh	L-threonine dehydrogenase
Tes	testis derived transcript
Tgm2	transglutaminase 2, C polypeptide
Timm13	translocase of inner mitochondrial membrane 13 homolog (yeast)
Timm17b	translocase of inner mitochondrial membrane 17b
Tlr4	toll-like receptor 4
Tmem143	transmembrane protein 143
Trak1	trafficking protein, kinesin binding 1
Trpa1	transient receptor potential cation channel, subfamily A, member 1
Tsfm	Ts translation elongation factor, mitochondrial
Txnip	thioredoxin interacting protein
Txnrd2	thioredoxin reductase 2
Ucp1	uncoupling protein 1 (mitochondrial, proton carrier)
Ucp3	uncoupling protein 3 (mitochondrial, proton carrier)
Wnt2	wingless-related MMTV integration site 2
Wwox	WW domain-containing oxidoreductase

NFkB Target Genes (199 genes)

Acta1	actin, alpha 1, skeletal muscle
Acy3	aspartoacylase (aminoacylase) 3
Adcy9	adenylate cyclase 9
Ahdc1	AT hook, DNA binding motif, containing 1
Ap3m2	adaptor-related protein complex 3, mu 2 subunit
Apln	apelin
Arhgef19	Rho guanine nucleotide exchange factor (GEF) 19
Aspn	asporin
Atoh8	atonal homolog 8 (Drosophila)
Bach1	BTB and CNC homology 1
Bmi1	Bmi1 polycomb ring finger oncogene
Bphl	biphenyl hydrolase-like (serine hydrolase, breast epithelial mucin-associated antigen)
C1s	complement component 1, s subcomponent
C8g	complement component 8, gamma polypeptide
Calcoco1	calcium binding and coiled coil domain 1
Casp4	caspase 4, apoptosis-related cysteine peptidase
Cbx8	chromobox homolog 8 (Drosophila Pc class)
Ccdc80	coiled-coil domain containing 80
Ccdc86	coiled-coil domain containing 86
Ccl2	chemokine (C-C motif) ligand 2
Ccl7	chemokine (C-C motif) ligand 7
Cdkn1a	cyclin-dependent kinase inhibitor 1A (P21)
Chka	choline kinase alpha

Chrnb1	cholinergic receptor, nicotinic, beta polypeptide 1 (muscle)
Cmtm3	CKLF-like MARVEL transmembrane domain containing 3
Cnn1	calponin 1
Cnp	2',3'-cyclic nucleotide 3' phosphodiesterase
Col27a1	collagen, type XXVII, alpha 1
Col3a1	collagen, type III, alpha 1
Col5a3	collagen, type V, alpha 3
Col6a1	collagen, type VI, alpha 1
Crem	cAMP responsive element modulator
Crip1	cysteine-rich protein 1 (intestinal)
Ctf1	cardiotrophin 1
Ctxn1	cortexin 1
Cx3cl1	chemokine (C-X3-C motif) ligand 1
Cxcl10	chemokine (C-X-C motif) ligand 10
Cxcl5	chemokine (C-X-C motif) ligand 5
Dcun1d1	DCN1, defective in cullin neddylation 1, domain containing 1 (S. cerevisiae)
Ddi2	DNA-damage inducible protein 2
Ddx58	DEAD (Asp-Glu-Ala-Asp) box polypeptide 58
Dennd2c	DENN/MADD domain containing 2C
Depdc6	DEP domain containing 6
Dffb	DNA fragmentation factor, beta subunit
Dusp1	dual specificity phosphatase 1
Dusp6	dual specificity phosphatase 6
Dusp7	dual specificity phosphatase 7
Edn1	endothelin 1
Eed	embryonic ectoderm development
Eif5	eukaryotic translation initiation factor 5
Emilin2	elastin microfibril interfacer 2
Extl3	exostoses (multiple)-like 3
Fbxl13	F-box and leucine-rich repeat protein 13
Fcgrt	Fc receptor, IgG, alpha chain transporter
Fkbp5	FK506 binding protein 5
Foxc2	forkhead box C2
Frs3	fibroblast growth factor receptor substrate 3
Fzd1	frizzled homolog 1 (Drosophila)
Gclc	glutamate-cysteine ligase, catalytic subunit
Gpr173	G-protein coupled receptor 173
Gpr18	G protein-coupled receptor 18
Grem1	gremlin 1
Grwd1	glutamate-rich WD repeat containing 1
Gtse1	G two S phase expressed protein 1
Hhat	hedgehog acyltransferase
Hhip	Hedgehog-interacting protein
Hiatl1	hippocampus abundant transcript-like 1
Hmga1	high mobility group AT-hook 1
Hmox1	heme oxygenase (decycling) 1
Hnrpdl	heterogeneous nuclear ribonucleoprotein D-like
Hsd3b7 h	ydroxy-delta-5-steroid dehydrogenase, 3 beta- and steroid delta-isomerase 7
Ifitm1	interferon induced transmembrane protein 1
Ifitm3	interferon induced transmembrane protein 3
Igsf9	immunoglobulin superfamily, member 9
Il1rap	interleukin 1 receptor accessory protein
Ing4	inhibitor of growth family, member 4
Irf1	interferon regulatory factor 1
Irx5	Iroquois related homeobox 5 (Drosophila)
Jun	Jun oncogene

Kcnk3	potassium channel, subfamily K, member 3
Kirrel3	kin of IRRE like 3 (Drosophila)
Klc4	kinesin light chain 4
Klf5	Kruppel-like factor 5
Klhl24	kelch-like 24 (Drosophila)
Klk10	kallikrein related-peptidase 10
Krt19	keratin 19
Lama2	laminin, alpha 2
Lancl3	LanC lantibiotic synthetase component C-like 3 (bacterial)
Larp2	La ribonucleoprotein domain family, member 2
Lbr	lamin B receptor
Lgals3bp	lectin, galactoside-binding, soluble, 3 binding protein
Limd2	LIM domain containing 2
Lrrc8c	leucine rich repeat containing 8 family, member C
Luzp1	leucine zipper protein 1
Lysmd1	LysM, putative peptidoglycan-binding, domain containing 1
Mafk	v-maf musculoaponeurotic fibrosarcoma oncogene family, protein K (avian)
Mapk14	mitogen-activated protein kinase 14
Mbnl1	muscleblind-like 1 (Drosophila)
Mcl1	myeloid cell leukemia sequence 1
Mcm3	minichromosome maintenance deficient 3 (S. cerevisiae)
Mdm2	transformed mouse 3T3 cell double minute 2
Mgea5	meningioma expressed antigen 5 (hyaluronidase)
Mpdz	multiple PDZ domain protein
Msx2	homeobox, msh-like 2
Mterfd2	MTERF domain containing 2
Narg1	NMDA receptor-regulated gene 1
Ndrg4	N-myc downstream regulated gene 4
Nfatc4	nuclear factor of activated T-cells, cytoplasmic, calcineurin-dependent 4
Nfkb2	nuclear factor of kappa light polypeptide gene enhancer in B-cells 2, p49/p100
Nfkbia	nuclear factor of kappa light polypeptide gene enhancer in B-cells inhibitor, alpha
Noc3l	nucleolar complex associated 3 homolog (S. cerevisiae)
Nol9	nucleolar protein 9
Nov	nephroblastoma overexpressed gene
Noxo1	NADPH oxidase organizer 1
Nr4a1	nuclear receptor subfamily 4, group A, member 1
Nrp2	neuropilin 2
Nup50	nucleoporin 50
Nupr1	nuclear protein 1
Ogn	osteoglycin
Osr1	odd-skipped related 1 (Drosophila)
Otub2	OTU domain, ubiquitin aldehyde binding 2
P2ry5	purinergic receptor P2Y, G-protein coupled, 5
Pak4	p21 (CDKN1A)-activated kinase 4
Pcdhb17	protocadherin beta 17
Pcgf5	polycomb group ring finger 5
Pde4b	phosphodiesterase 4B, cAMP specific
Pdgfa	platelet derived growth factor, alpha
Pdgfd	platelet-derived growth factor, D polypeptide
Pdgfrl	platelet-derived growth factor receptor-like
Pdk2	pyruvate dehydrogenase kinase, isoenzyme 2
Phf21a	PHD finger protein 21A
Pld3	phospholipase D family, member 3
Plk2	polo-like kinase 2 (Drosophila)
Plk4	polo-like kinase 4 (Drosophila)
Pold4	polymerase (DNA-directed), delta 4

Polk	polymerase (DNA directed), kappa
Pom121	nuclear pore membrane protein 121
Postn	periostin, osteoblast specific factor
Ppm1d	protein phosphatase 1D magnesium-dependent, delta isoform
Ppp3cb	protein phosphatase 3, catalytic subunit, beta isoform
Prkci	protein kinase C, iota
Prss23	protease, serine, 23
Ptgfr	prostaglandin F receptor
Ptk2b	PTK2 protein tyrosine kinase 2 beta
Pxmp4	peroxisomal membrane protein 4
Rai14	retinoic acid induced 14
Ralgds	ral guanine nucleotide dissociation stimulator
Ramp2 r	eceptor (calcitonin) activity modifying protein 2
Rassf6	Ras association (RalGDS/AF-6) domain family member 6
Rbm19	RNA binding motif protein 19
Rcn3	reticulocalbin 3, EF-hand calcium binding domain
Rgs3	regulator of G-protein signaling 3
Rnpc3	RNA-binding region (RNP1, RRM) containing 3
Ropn1l	ropporin 1-like
Rrad	Ras-related associated with diabetes
Rras	Harvey rat sarcoma oncogene, subgroup R
Sdf2l1	stromal cell-derived factor 2-like 1
Selm	selenoprotein M
Sema3d	sema domain, immunoglobulin domain (Ig), short basic domain, secreted, (semaphorin) 3D
Sf1	splicing factor 1
Shmt1	serine hydroxymethyltransferase 1 (soluble)
Slc16a1	solute carrier family 16 (monocarboxylic acid transporters), member 1
Slc19a2	solute carrier family 19 (thiamine transporter), member 2
Slc26a3	solute carrier family 26, member 3
Slc35d1	solute carrier family 35 (UDP-glucuronic acid/UDP-N-acetylgalactosamine dual transporter), member D1
Smad4	MAD homolog 4 (Drosophila)
Socs4	suppressor of cytokine signaling 4
Sp3	trans-acting transcription factor 3
Spata5	spermatogenesis associated 5
Spon2	spondin 2, extracellular matrix protein
Ssbp3	single-stranded DNA binding protein 3
Stat3	signal transducer and activator of transcription 3
Stoml1	stomatin-like 1
Strbp	spermatid perinuclear RNA binding protein
Sulf2	sulfatase 2
Suv420h2	suppressor of variegation 4-20 homolog 2 (Drosophila)
Syncrip	synaptotagmin binding, cytoplasmic RNA interacting protein
Tapbpl	TAP binding protein-like
Tcea3	transcription elongation factor A (SII), 3
Tlr2	toll-like receptor 2
Tm7sf2	transmembrane 7 superfamily member 2
Tmem160	transmembrane protein 160
Tmpo	thymopoietin
Traf1	TNF receptor-associated factor 1
Tram1	translocating chain-associating membrane protein 1
Tspyl1	testis-specific protein, Y-encoded-like 1
Tspyl4	TSPY-like 4
Ttc30b	tetratricopeptide repeat domain 30B
Txlnb	taxilin beta

Tyk2	tyrosine kinase 2
Ubd	ubiquitin D
Ubl3	ubiquitin-like 3
Wbscr27	Williams Beuren syndrome chromosome region 27 (human)
Wnt10b	wingless related MMTV integration site 10b
Wwc1	WW, C2 and coiled-coil domain containing 1
Yipf2	Yip1 domain family, member 2
Zbtb41	zinc finger and BTB domain containing 41 homolog
Zc3h10	zinc finger CCCH type containing 10
Zcchc8	zinc finger, CCHC domain containing 8

Response to Oxidative Stress (51 genes)

Als2	amyotrophic lateral sclerosis 2 (juvenile) homolog (human)
Angptl7	angiopoietin-like 7
Apoa4	apolipoprotein A-IV
Aptx	aprataxin
Arnt	aryl hydrocarbon receptor nuclear translocator
Atox1	ATX1 (antioxidant protein 1) homolog 1 (yeast)
Bcl2	B-cell leukemia/lymphoma 2
Cbx6	chromobox homolog 6
Ccl5	chemokine (C-C motif) ligand 5
Cln8	ceroid-lipofuscinosis, neuronal 8
Cryab	crystallin, alpha B
Cygb	cytoglobin
Dusp1	dual specificity phosphatase 1
Eomes	eomesodermin homolog (Xenopus laevis)
Epas1	endothelial PAS domain protein 1
Ercc2	excision repair cross-complementing rodent repair deficiency, complementation group 2
Gab1	growth factor receptor bound protein 2-associated protein 1
Gatm	glycine amidinotransferase (L-arginine:glycine amidinotransferase)
Gclc	glutamate-cysteine ligase, catalytic subunit
Gpx1	glutathione peroxidase 1
Gpx2	glutathione peroxidase 2
Gpx5	glutathione peroxidase 5
Gpx6	glutathione peroxidase 6
Gpx7	glutathione peroxidase 7
Hif1a	hypoxia inducible factor 1, alpha subunit
Hmox1	heme oxygenase (decycling) 1
Hmox2	heme oxygenase (decycling) 2
Jun	Jun oncogene
Mpo	myeloperoxidase
Msra	methionine sulfoxide reductase A
Mtf1	metal response element binding transcription factor 1
Mutyh	mutY homolog (E. coli)
Myh7	myosin, heavy polypeptide 7, cardiac muscle, beta
Neil1	nei endonuclease VIII-like 1 (E. coli)
Nfkb1	nuclear factor of kappa light polypeptide gene enhancer in B-cells 1, p105
Olr1	oxidized low density lipoprotein (lectin-like) receptor 1
Oxsr1	oxidative-stress responsive 1
Park7	Parkinson disease (autosomal recessive, early onset) 7
Pnkp	polynucleotide kinase 3'- phosphatase
Prdx3	peroxiredoxin 3
Scara3	scavenger receptor class A, member 3
Sdc1	syndecan 1
Sgk2	serum/glucocorticoid regulated kinase 2
Shc1	src homology 2 domain-containing transforming protein C1

Sod2	superoxide dismutase 2, mitochondrial
Stat1	signal transducer and activator of transcription 1
Tlr4	toll-like receptor 4
Trpa1	transient receptor potential cation channel, subfamily A, member 1
Txnip	thioredoxin interacting protein
Txnrd2	thioredoxin reductase 2
Ucp3	uncoupling protein 3 (mitochondrial, proton carrier)

Alzheimers Disease Brain (676 genes)

Abca1	ATP-binding cassette, sub-family A (ABC1), member 1
Abcc10	ATP-binding cassette, sub-family C (CFTR/MRP), member 10
Abcd4	ATP-binding cassette, sub-family D (ALD), member 4
Abl1	c-abl oncogene 1, receptor tyrosine kinase
Ablim1	actin-binding LIM protein 1
Acrv1	acrosomal vesicle protein 1
Acvr1b	activin A receptor, type 1B
Adam21	a disintegrin and metallopeptidase domain 21
Adamdec1	ADAM-like, decysin 1
Adarb2	adenosine deaminase, RNA-specific, B2
Adcy2	adenylate cyclase 2
Adcy3	adenylate cyclase 3
Adcy7	adenylate cyclase 7
Adipoq	adiponectin, C1Q and collagen domain containing
Aebp1	AE binding protein 1
Aff1	AF4/FMR2 family, member 1
Ahctf1	AT hook containing transcription factor 1
Ak1	adenylate kinase 1
Akap1	A kinase (PRKA) anchor protein 1
Akap9	A kinase (PRKA) anchor protein (yotiao) 9
Akt3	thymoma viral proto-oncogene 3
Aldh1a2	aldehyde dehydrogenase family 1, subfamily A2
Amotl2	angiomotin-like 2
Ampd3	adenosine monophosphate deaminase 3
Angpt1	angiopoietin 1
Angptl2	angiopoietin-like 2
Aoc2	amine oxidase, copper containing 2 (retina-specific)
Ap3s2	adaptor-related protein complex 3, sigma 2 subunit
Apba3	amyloid beta (A4) precursor protein-binding, family A, member 3
Apobec1	apolipoprotein B mRNA editing enzyme, catalytic polypeptide 1
Aptx	aprataxin
Aqp1	aquaporin 1
Aqp6	aquaporin 6
Arfgap1	ADP-ribosylation factor GTPase activating protein 1
Arg2	arginase type II
Arhgap1	Rho GTPase activating protein 1
Arhgap17	Rho GTPase activating protein 17
Arhgap26	Rho GTPase activating protein 26
Arhgap6	Rho GTPase activating protein 6
Arhgef10	Rho guanine nucleotide exchange factor (GEF) 10
Arhgef15	Rho guanine nucleotide exchange factor (GEF) 15
Arid1a	AT rich interactive domain 1A (SWI-like)
Arid4b	AT rich interactive domain 4B (RBP1-like)
Arih2	ariadne homolog 2 (Drosophila)
Ascl1	achaete-scute complex homolog 1 (Drosophila)
Atg3	autophagy-related 3 (yeast)

Atg4b	autophagy-related 4B (yeast)
Atp10d	ATPase, class V, type 10D
Atp6v0e	ATPase, H+ transporting, lysosomal V0 subunit E
Atp8b1	ATPase, class I, type 8B, member 1
Avpr1a	arginine vasopressin receptor 1A
Bad	BCL2-associated agonist of cell death
Bag1	BCL2-associated athanogene 1
Baz2b	bromodomain adjacent to zinc finger domain, 2B
Bcan	brevican
Bcl2	B-cell leukemia/lymphoma 2
Bgn	biglycan
Bin1	bridging integrator 1
Bin3	bridging integrator 3
Bmp10	bone morphogenetic protein 10
Brd2	bromodomain containing 2
Brd4	bromodomain containing 4
Btg2	B-cell translocation gene 2, anti-proliferative
Btn3a3	butyrophilin, subfamily 3, member A3
Btrc	beta-transducin repeat containing protein
C1r	---
C1s	complement component 1, s subcomponent
Cacna1a	calcium channel, voltage-dependent, P/Q type, alpha 1A subunit
Cacna1g	calcium channel, voltage-dependent, T type, alpha 1G subunit
Cald1	caldesmon 1
Cant1	calcium activated nucleotidase 1
Casc3	cancer susceptibility candidate 3
Caskin2	CASK-interacting protein 2
Casp3	caspase 3
Casp4	caspase 4, apoptosis-related cysteine peptidase
Cast	calpastatin
Cbfb	core binding factor beta
Ccdc69	coiled-coil domain containing 69
Ccdc9	coiled-coil domain containing 9
Cchcr1	coiled-coil alpha-helical rod protein 1
Ccrn4l	CCR4 carbon catabolite repression 4-like (S. cerevisiae)
Cd22	CD22 antigen
Cd248	CD248 antigen, endosialin
Cd84	CD84 antigen
Cd86	CD86 antigen
Cdc14a	CDC14 cell division cycle 14 homolog A (S. cerevisiae)
Cdc42ep4	CDC42 effector protein (Rho GTPase binding) 4
Cdh4	cadherin 4
Cdipt	CDP-diacylglycerol--inositol 3-phosphatidyltransferase (phosphatidylinositol synthase)
Cdkn2c	cyclin-dependent kinase inhibitor 2C (p18, inhibits CDK4)
Cebpa	CCAAT/enhancer binding protein (C/EBP), alpha
Cep350	centrosomal protein 350
Cflar	CASP8 and FADD-like apoptosis regulator
Ch25h	cholesterol 25-hydroxylase
Chrna6	cholinergic receptor, nicotinic, alpha polypeptide 6
Ciz1	CDKN1A interacting zinc finger protein 1
Cldn18	claudin 18
Cldn5	claudin 5
Clec2d	C-type lectin domain family 2, member d
Clec7a	C-type lectin domain family 7, member a
Cmtm6	CKLF-like MARVEL transmembrane domain containing 6
Cnot2	CCR4-NOT transcription complex, subunit 2

Cnp	2',3'-cyclic nucleotide 3' phosphodiesterase
Cog2	component of oligomeric golgi complex 2
Col18a1	collagen, type XVIII, alpha 1
Col1a2	collagen, type I, alpha 2
Col4a3	collagen, type IV, alpha 3
Col4a5	collagen, type IV, alpha 5
Col6a3	collagen, type VI, alpha 3
Col8a2	collagen, type VIII, alpha 2
Cradd	CASP2 and RIPK1 domain containing adaptor with death domain
Crhbp	corticotropin releasing hormone binding protein
Crkl	v-crk sarcoma virus CT10 oncogene homolog (avian)-like
Csf1	colony stimulating factor 1 (macrophage)
Csk	c-src tyrosine kinase
Csnk1a1	casein kinase 1, alpha 1
Ctbp1	C-terminal binding protein 1
Ctbp2	C-terminal binding protein 2
Ctdsp1	CTD (carboxy-terminal domain, RNA polymerase II, polypeptide A) small phosphatase 1
Ctdsp2	CTD (carboxy-terminal domain, RNA polymerase II, polypeptide A) small phosphatase 2
Ctdspl	CTD (carboxy-terminal domain, RNA polymerase II, polypeptide A) small phosphatase-like
Ctnna1	catenin (cadherin associated protein), alpha 1
Ctns	cystinosis, nephropathic
Ctsh	cathepsin H
Cul4a	cullin 4A
Cul5	cullin 5
Cxcl2	chemokine (C-X-C motif) ligand 2
Cyhr1	cysteine and histidine rich 1
Cyp11a1	cytochrome P450, family 11, subfamily a, polypeptide 1
Cyp39a1	cytochrome P450, family 39, subfamily a, polypeptide 1
Daam1	dishevelled associated activator of morphogenesis 1
Daam2	dishevelled associated activator of morphogenesis 2
Dag1	dystroglycan 1
Dao	D-amino acid oxidase
Dapk2	death-associated protein kinase 2
Dazap2	DAZ associated protein 2
Dbf4	DBF4 homolog (S. cerevisiae)
Dcn	decorin
Ddr2	discoidin domain receptor family, member 2
Ddx27	DEAD (Asp-Glu-Ala-Asp) box polypeptide 27
Ddx6	DEAD (Asp-Glu-Ala-Asp) box polypeptide 6
Dgkg	diacylglycerol kinase, gamma
Dhrs9	dehydrogenase/reductase (SDR family) member 9
Dicer1	Dicer1, Dcr-1 homolog (Drosophila)
Dlg5	discs, large homolog 5 (Drosophila)
Dmc1	DMC1 dosage suppressor of mck1 homolog, meiosis-specific homologous recombination (yeast)
Dmd	dystrophin, muscular dystrophy
Dnajb2	DnaJ (Hsp40) homolog, subfamily B, member 2
Dnajc4	DnaJ (Hsp40) homolog, subfamily C, member 4
Dpf3	D4, zinc and double PHD fingers, family 3
Dpysl3	dihydropyrimidinase-like 3
Drd2	dopamine receptor 2
Dsc3	desmocollin 3
Dtna	dystrobrevin alpha
Dusp9	dual specificity phosphatase 9
Dut	deoxyuridine triphosphatase

Ecm2	extracellular matrix protein 2, female organ and adipocyte specific
Edar	ectodysplasin-A receptor
Ednra	endothelin receptor type A
Eef1d	eukaryotic translation elongation factor 1 delta (guanine nucleotide exchange protein)
Efemp1	epidermal growth factor-containing fibulin-like extracellular matrix protein 1
Efemp2	epidermal growth factor-containing fibulin-like extracellular matrix protein 2
Egf	epidermal growth factor
Ehd1	EH-domain containing 1
Eif2ak2	eukaryotic translation initiation factor 2-alpha kinase 2
Eif2c1	eukaryotic translation initiation factor 2C, 1
Elk1	ELK1, member of ETS oncogene family
Eml2	echinoderm microtubule associated protein like 2
Emp1	epithelial membrane protein 1
Enah	enabled homolog (Drosophila)
Ewsr1	Ewing sarcoma breakpoint region 1
Exph5	exophilin 5
Fam107a	family with sequence similarity 107, member A
Fbln1	fibulin 1
Fbn1	fibrillin 1
Fbn2	fibrillin 2
Fbxl5	F-box and leucine-rich repeat protein 5
Fbxl7	F-box and leucine-rich repeat protein 7
Fbxo9	f-box protein 9
Fbxw4	F-box and WD-40 domain protein 4
Fetub	fetuin beta
Fez2	fasciculation and elongation protein zeta 2 (zygin II)
Fgf18	fibroblast growth factor 18
Fgfr1	fibroblast growth factor receptor 1
Fgfr3	fibroblast growth factor receptor 3
Fmo2	flavin containing monooxygenase 2
Frat1	frequently rearranged in advanced T-cell lymphomas
Fut2	fucosyltransferase 2
Fyco1	FYVE and coiled-coil domain containing 1
Fyn	Fyn proto-oncogene
Fzr1	fizzy/cell division cycle 20 related 1 (Drosophila)
G6pc2	glucose-6-phosphatase, catalytic, 2
Gab2	growth factor receptor bound protein 2-associated protein 2
Gabrq	gamma-aminobutyric acid (GABA) A receptor, subunit theta
Gadd45b	growth arrest and DNA-damage-inducible 45 beta
Gadd45g	growth arrest and DNA-damage-inducible 45 gamma
Galnt1	UDP-N-acetyl-alpha-D-galactosamine:polypeptide N-acetylgalactosaminyltransferase 1
Galnt6	UDP-N-acetyl-alpha-D-galactosamine:polypeptide N-acetylgalactosaminyltransferase 6
Gas2l1	growth arrest-specific 2 like 1
Gbp2	guanylate binding protein 2
Gcc1	golgi coiled coil 1
Gclc	glutamate-cysteine ligase, catalytic subunit
Gdf9	growth differentiation factor 9
Gfap	glial fibrillary acidic protein
Gfpt2	glutamine fructose-6-phosphate transaminase 2
Gga1	golgi associated, gamma adaptin ear containing, ARF binding protein 1
Gga2	golgi associated, gamma adaptin ear containing, ARF binding protein 2
Gja1	gap junction protein, alpha 1
Glra3	glycine receptor, alpha 3 subunit
Glt25d2	glycosyltransferase 25 domain containing 2
Gmpr	guanosine monophosphate reductase
Gna12	guanine nucleotide binding protein, alpha 12

Golga1	golgi autoantigen, golgin subfamily a, 1
Golga3	golgi autoantigen, golgin subfamily a, 3
Gosr2	golgi SNAP receptor complex member 2
Gpc4	glypican 4
Gpr175	G protein-coupled receptor 175
Gpr4	G protein-coupled receptor 4
Gpr44	G protein-coupled receptor 44
Gpr56	G protein-coupled receptor 56
Gpr64	G protein-coupled receptor 64
Gpr65	G-protein coupled receptor 65
Gprc5b	G protein-coupled receptor, family C, group 5, member B
Gpsm3	G-protein signalling modulator 3 (AGS3-like, C. elegans)
Grb10	growth factor receptor bound protein 10
Grk5	G protein-coupled receptor kinase 5
Grk6	G protein-coupled receptor kinase 6
Grm6	glutamate receptor, metabotropic 6
Gstm5	glutathione S-transferase, mu 5
Gstt2	glutathione S-transferase, theta 2
Gtf3c1	general transcription factor III C 1
Gtf3c2	general transcription factor IIIC, polypeptide 2, beta
Gtpbp8	GTP-binding protein 8 (putative)
Gtse1	G two S phase expressed protein 1
H2afj	H2A histone family, member J
H3f3a	H3 histone, family 3A
Hapln2	hyaluronan and proteoglycan link protein 2
Hbegf	heparin-binding EGF-like growth factor
Hdac4	histone deacetylase 4
Heph	hephaestin
Hes1	hairy and enhancer of split 1 (Drosophila)
Hfe	hemochromatosis
Hgf	hepatocyte growth factor
Hip1r	huntingtin interacting protein 1 related
Hipk2	homeodomain interacting protein kinase 2
Hmox1	heme oxygenase (decycling) 1
Homer3	homer homolog 3 (Drosophila)
Hoxb5	homeo box B5
Hs1bp3	HCLS1 binding protein 3
Hs2st1	heparan sulfate 2-O-sulfotransferase 1
Hsbp1	heat shock factor binding protein 1
Hspb2	heat shock protein 2
Ier2	immediate early response 2
Ifitm2	---
Igfbp5	insulin-like growth factor binding protein 5
Igsf6	immunoglobulin superfamily, member 6
Il10ra	interleukin 10 receptor, alpha
Il10rb	interleukin 10 receptor, beta
Il13ra1	interleukin 13 receptor, alpha 1
Il15	interleukin 15
Il17ra	interleukin 17 receptor A
Il1a	interleukin 1 alpha
Il1r1	interleukin 1 receptor, type I
Il22ra1	interleukin 22 receptor, alpha 1
Il2rg	interleukin 2 receptor, gamma chain
Il6	interleukin 6
Ing3	inhibitor of growth family, member 3
Ing4	inhibitor of growth family, member 4

Insig1	insulin induced gene 1
Irf7	interferon regulatory factor 7
Islr	immunoglobulin superfamily containing leucine-rich repeat
Itga7	integrin alpha 7
Itgb4	integrin beta 4
Itpk1	inositol 1,3,4-triphosphate 5/6 kinase
Itpkc	inositol 1,4,5-trisphosphate 3-kinase C
Jak3	Janus kinase 3
Jrk	jerky
Jun	Jun oncogene
Jund	Jun proto-oncogene related gene d
Kcnj14	potassium inwardly-rectifying channel, subfamily J, member 14
Kcnj15	potassium inwardly-rectifying channel, subfamily J, member 15
Kcnk10	potassium channel, subfamily K, member 10
Kcnk5	potassium channel, subfamily K, member 5
Khsrp	KH-type splicing regulatory protein
Kif3b	kinesin family member 3B
Kif5b	kinesin family member 5B
Klf1	Kruppel-like factor 1 (erythroid)
Klf2	Kruppel-like factor 2 (lung)
Klf5	Kruppel-like factor 5
Klf7	Kruppel-like factor 7 (ubiquitous)
Klhl20	kelch-like 20 (Drosophila)
Kpna1	karyopherin (importin) alpha 1
Kpnb1	karyopherin (importin) beta 1
Lama2	laminin, alpha 2
Lama4	laminin, alpha 4
Lamc1	laminin, gamma 1
Lamp1	lysosomal-associated membrane protein 1
Larp4	La ribonucleoprotein domain family, member 4
Lef1	lymphoid enhancer binding factor 1
Lgals3bp	lectin, galactoside-binding, soluble, 3 binding protein
Lgals9	lectin, galactose binding, soluble 9
Lhx3	LIM homeobox protein 3
Lifr	leukemia inhibitory factor receptor
Limk2	LIM motif-containing protein kinase 2
Lipg	lipase, endothelial
Lmbr1l	limb region 1 like
Lpp	LIM domain containing preferred translocation partner in lipoma
Lrp10	low-density lipoprotein receptor-related protein 10
Lrp4	low density lipoprotein receptor-related protein 4
Lrp6	low density lipoprotein receptor-related protein 6
Lrrc1	leucine rich repeat containing 1
Lsm14a	LSM14 homolog A (SCD6, S. cerevisiae)
Lss	lanosterol synthase
Ltbp2	latent transforming growth factor beta binding protein 2
Ltbp3	latent transforming growth factor beta binding protein 3
Lyst	lysosomal trafficking regulator
Lztr1	leucine-zipper-like transcriptional regulator, 1
Macf1	microtubule-actin crosslinking factor 1
Mafb	v-maf musculoaponeurotic fibrosarcoma oncogene family, protein B (avian)
Mafg	v-maf musculoaponeurotic fibrosarcoma oncogene family, protein G (avian)
Man2b1	mannosidase 2, alpha B1
Map2k3	mitogen-activated protein kinase kinase 3
Map2k7	mitogen-activated protein kinase kinase 7
Map3k11	mitogen-activated protein kinase kinase kinase 11

Map3k14	mitogen-activated protein kinase kinase kinase 14
Map3k3	mitogen-activated protein kinase kinase kinase 3
Masp1	mannan-binding lectin serine peptidase 1
Mat2a	methionine adenosyltransferase II, alpha
Matn2	matrilin 2
Max	Max protein
Mbp	myelin basic protein
Mdm1	transformed mouse 3T3 cell double minute 1
Met	met proto-oncogene
Mgat1	mannoside acetylglucosaminyltransferase 1
Mitf	microphthalmia-associated transcription factor
Mknk2	MAP kinase-interacting serine/threonine kinase 2
Mkrn1	makorin, ring finger protein, 1
Mmp12	matrix metallopeptidase 12
Mmp16	matrix metallopeptidase 16
Mr1	major histocompatibility complex, class I-related
Mras	muscle and microspikes RAS
Mrc2	mannose receptor, C type 2
Mrp63	mitochondrial ribosomal protein 63
Msh4	mutS homolog 4 (E. coli)
Msx1	homeobox, msh-like 1
Mta1	metastasis associated 1
Mtf1	metal response element binding transcription factor 1
Mtmr11	myotubularin related protein 11
Muc1	mucin 1, transmembrane
Mutyh	mutY homolog (E. coli)
Mvk	mevalonate kinase
Mx2	myxovirus (influenza virus) resistance 2
Mxd4	Max dimerization protein 4
Mycn	v-myc myelocytomatosis viral related oncogene, neuroblastoma derived (avian)
Myl4	myosin, light polypeptide 4
Mylk	myosin, light polypeptide kinase
Myo10	myosin X
Myo1e	myosin IE
Myo5c	myosin VC
Myo6	myosin VI
Myo9b	myosin IXb
Myom1	myomesin 1
Myst4	MYST histone acetyltransferase monocytic leukemia 4
N4bp1	NEDD4 binding protein 1
Ncam1	neural cell adhesion molecule 1
Ncoa3	nuclear receptor coactivator 3
Ncor2	nuclear receptor co-repressor 2
Ncstn	nicastrin
Ndst1	N-deacetylase/N-sulfotransferase (heparan glucosaminyl) 1
Neil1	nei endonuclease VIII-like 1 (E. coli)
Nek1	NIMA (never in mitosis gene a)-related expressed kinase 1
Nek7	NIMA (never in mitosis gene a)-related expressed kinase 7
Nek9	NIMA (never in mitosis gene a)-related expressed kinase 9
Neu3	neuraminidase 3
Nfe2l1	nuclear factor, erythroid derived 2,-like 1
Nfib	nuclear factor I/B
Nfkb1	nuclear factor of kappa light polypeptide gene enhancer in B-cells 1, p105
Nfkbia	nuclear factor of kappa light polypeptide gene enhancer in B-cells inhibitor, alpha
Nfx1	nuclear transcription factor, X-box binding 1
Nid1	nidogen 1

Nktr	natural killer tumor recognition sequence
Nnmt	nicotinamide N-methyltransferase
Notch4	Notch gene homolog 4 (Drosophila)
Npas3	neuronal PAS domain protein 3
Npc1	Niemann Pick type C1
Nr1d1	nuclear receptor subfamily 1, group D, member 1
Nr4a3	nuclear receptor subfamily 4, group A, member 3
Nrp2	neuropilin 2
Nrxn2	neurexin II
Ntrk3	neurotrophic tyrosine kinase, receptor, type 3
Nufip1	nuclear fragile X mental retardation protein interacting protein 1
Nup98	nucleoporin 98
Nyx	nyctalopin
Ogdh	oxoglutarate dehydrogenase (lipoamide)
Ogg1	8-oxoguanine DNA-glycosylase 1
Ophn1	oligophrenin 1
Pak2	p21 (CDKN1A)-activated kinase 2
Pak4	p21 (CDKN1A)-activated kinase 4
Pbx2	pre B-cell leukemia transcription factor 2
Pbxip1	pre-B-cell leukemia transcription factor interacting protein 1
Pcdh17	protocadherin 17
Pcm1	pericentriolar material 1
Pcsk5	proprotein convertase subtilisin/kexin type 5
Pdcd4	programmed cell death 4
Pde4c	phosphodiesterase 4C, cAMP specific
Pde4d	phosphodiesterase 4D, cAMP specific
Pdgfb	platelet derived growth factor, B polypeptide
Pdgfrb	platelet derived growth factor receptor, beta polypeptide
Pdlim3	PDZ and LIM domain 3
Pdlim4	PDZ and LIM domain 4
Pdpn	podoplanin
Pecam1	platelet/endothelial cell adhesion molecule 1
Pelp1	proline, glutamic acid and leucine rich protein 1
Per1	period homolog 1 (Drosophila)
Per2	period homolog 2 (Drosophila)
Pfkfb3	6-phosphofructo-2-kinase/fructose-2,6-biphosphatase 3
Pgls	6-phosphogluconolactonase
Pgm3	phosphoglucomutase 3
Phc2	polyhomeotic-like 2 (Drosophila)
Phf20	PHD finger protein 20
Phf21a	PHD finger protein 21A
Pias4	protein inhibitor of activated STAT 4
Piga	phosphatidylinositol glycan anchor biosynthesis, class A
Pik3c3	phosphoinositide-3-kinase, class 3
Pim1	proviral integration site 1
Pitpnc1	phosphatidylinositol transfer protein, cytoplasmic 1
Pkd1	polycystic kidney disease 1 homolog
Pla2g5	phospholipase A2, group V
Pla2g6	phospholipase A2, group VI
Plcg2	phospholipase C, gamma 2
Pld2	phospholipase D2
Plec1	plectin 1
Plekha5	pleckstrin homology domain containing, family A member 5
Plod2	procollagen lysine, 2-oxoglutarate 5-dioxygenase 2
Plod3	procollagen-lysine, 2-oxoglutarate 5-dioxygenase 3
Plscr3	phospholipid scramblase 3

Pml	promyelocytic leukemia
Pogz	pogo transposable element with ZNF domain
Polh	polymerase (DNA directed), eta (RAD 30 related)
Polr1b	polymerase (RNA) I polypeptide B
Polrmt	polymerase (RNA) mitochondrial (DNA directed)
Ppard	peroxisome proliferator activator receptor delta
Ppfibp2	protein tyrosine phosphatase, receptor-type, F interacting protein, binding protein 2
Ppm1b	protein phosphatase 1B, magnesium dependent, beta isoform
Ppm1d	protein phosphatase 1D magnesium-dependent, delta isoform
Ppp1r13l	protein phosphatase 1, regulatory (inhibitor) subunit 13 like
Ppp1r1a	protein phosphatase 1, regulatory (inhibitor) subunit 1A
Ppp1r9a	protein phosphatase 1, regulatory (inhibitor) subunit 9A
Prcp	prolylcarboxypeptidase (angiotensinase C)
Prdx3	peroxiredoxin 3
Prl	prolactin
Prlr	prolactin receptor
Prox1	prospero-related homeobox 1
Prrx1	paired related homeobox 1
Pskh1	protein serine kinase H1
Psmf1	proteasome (prosome, macropain) inhibitor subunit 1
Ptcra	pre T-cell antigen receptor alpha
Ptgis	prostaglandin I2 (prostacyclin) synthase
Ptp4a2	protein tyrosine phosphatase 4a2
Ptp4a3	protein tyrosine phosphatase 4a3
Ptprf	protein tyrosine phosphatase, receptor type, F
Ptprk	protein tyrosine phosphatase, receptor type, K
Ptrf	polymerase I and transcript release factor
Pttg1ip	pituitary tumor-transforming 1 interacting protein
Pura	purine rich element binding protein A
Pxn	paxillin
Rab1b	RAB1B, member RAS oncogene family
Rab22a	RAB22A, member RAS oncogene family
Rab31	RAB31, member RAS oncogene family
Rabep1	rabaptin, RAB GTPase binding effector protein 1
Rala	v-ral simian leukemia viral oncogene homolog A (ras related)
Ralbp1	ralA binding protein 1
Ralgds	ral guanine nucleotide dissociation stimulator
Ralgps1	Ral GEF with PH domain and SH3 binding motif 1
Raly	hnRNP-associated with lethal yellow
Ramp1	receptor (calcitonin) activity modifying protein 1
Rarres2	retinoic acid receptor responder (tazarotene induced) 2
Rasgrp2	RAS, guanyl releasing protein 2
Rbak	RB-associated KRAB repressor
Rbbp6	retinoblastoma binding protein 6
Rbm4b	RNA binding motif protein 4B
Rbms2	RNA binding motif, single stranded interacting protein 2
Rbms3	RNA binding motif, single stranded interacting protein
Rfx4	regulatory factor X, 4 (influences HLA class II expression)
Rgs12	regulator of G-protein signaling 12
Rgs16	regulator of G-protein signaling 16
Rheb	RAS-homolog enriched in brain
Rims2 r	egulating synaptic membrane exocytosis 2
Rin3	Ras and Rab interactor 3
Rln1	relaxin 1
Rnf130	ring finger protein 130
Rnf24	ring finger protein 24

Rnf8	ring finger protein 8
Rnmtl1	RNA methyltransferase like 1
Rnpepl1	arginyl aminopeptidase (aminopeptidase B)-like 1
Rod1	ROD1 regulator of differentiation 1 (S. pombe)
Ros1	Ros1 proto-oncogene
Rpgrip1	retinitis pigmentosa GTPase regulator interacting protein 1
Rps28	---
Rps6ka1	ribosomal protein S6 kinase polypeptide 1
Rps6ka5	ribosomal protein S6 kinase, polypeptide 5
Rreb1	ras responsive element binding protein 1
Rsf1	remodeling and spacing factor 1
Rufy1	RUN and FYVE domain containing 1
Runx2	runt related transcription factor 2
Rxrb	retinoid X receptor beta
Rxrg	retinoid X receptor gamma
Rybp	RING1 and YY1 binding protein
Sall2	sal-like 2 (Drosophila)
Sap30bp	SAP30 binding protein
Sav1	salvador homolog 1 (Drosophila)
Scamp3	secretory carrier membrane protein 3
Scarb1	scavenger receptor class B, member 1
Scly	selenocysteine lyase
Sec14l1	SEC14-like 1 (S. cerevisiae)
Sec63	SEC63-like (S. cerevisiae)
Sell	selectin, lymphocyte
Sema3c	sema domain, immunoglobulin domain (Ig), short basic domain, secreted, (semaphorin) 3C
Sema3f	sema domain, immunoglobulin domain (Ig), short basic domain, secreted, (semaphorin) 3F
Sema4f	sema domain, immunoglobulin domain (Ig), TM domain, and short cytoplasmic domain
Senp3	SUMO/sentrin specific peptidase 3
Serping1	serine (or cysteine) peptidase inhibitor, clade G, member 1
Serpini2	serine (or cysteine) peptidase inhibitor, clade I, member 2
Sertad2	SERTA domain containing 2
Sertad3	SERTA domain containing 3
Set	SET translocation
Sf3b3	splicing factor 3b, subunit 3
Sfrp4	secreted frizzled-related protein 4
Sfrs16	splicing factor, arginine/serine-rich 16
Sftpc	surfactant associated protein C
Sfxn3	sideroflexin 3
Sgk2	serum/glucocorticoid regulated kinase 2
Sgpl1	sphingosine phosphate lyase 1
Sgta	small glutamine-rich tetratricopeptide repeat (TPR)-containing, alpha
Sh3bp2	SH3-domain binding protein 2
Sh3gl3	SH3-domain GRB2-like 3
Sirt2	sirtuin 2 (silent mating type information regulation 2, homolog) 2 (S. cerevisiae)
Sirt6	sirtuin 6 (silent mating type information regulation 2, homolog) 6 (S. cerevisiae)
Slamf1	signaling lymphocytic activation molecule family member 1
Slc11a1	solute carrier family 11 (proton-coupled divalent metal ion transporters), member 1
Slc12a3	solute carrier family 12, member 3
Slc12a7	solute carrier family 12, member 7
Slc13a1	solute carrier family 13 (sodium/sulfate symporters), member 1
Slc14a1	solute carrier family 14 (urea transporter), member 1
Slc1a7	solute carrier family 1 (glutamate transporter), member 7
Slc26a2	solute carrier family 26 (sulfate transporter), member 2

Slc29a2	solute carrier family 29 (nucleoside transporters), member 2
Slc35a2	solute carrier family 35 (UDP-galactose transporter), member A2
Slc35a3	solute carrier family 35 (UDP-N-acetylglucosamine (UDP-GlcNAc) transporter), member 3
Slc35c1	solute carrier family 35, member C1
Slc4a4	solute carrier family 4 (anion exchanger), member 4
Slc7a5	solute carrier family 7 (cationic amino acid transporter, y+ system), member 5
Slco3a1	solute carrier organic anion transporter family, member 3a1
Smad2	MAD homolog 2 (Drosophila)
Smad5	MAD homolog 5 (Drosophila)
Smarcc2	SWI/SNF related, matrix associated, actin dependent regulator of chromatin, subfamily c, member 2
Smarcd1	SWI/SNF related, matrix associated, actin dependent regulator of chromatin, subfamily d, member 1
Smc1a	structural maintenance of chromosomes 1A
Smox	spermine oxidase
Snai2	snail homolog 2 (Drosophila)
Snrk	SNF related kinase
Snta1	syntrophin, acidic 1
Snx11	sorting nexin 11
Snx6	sorting nexin 6
Socs3	suppressor of cytokine signaling 3
Sod2	superoxide dismutase 2, mitochondrial
Sorbs1	sorbin and SH3 domain containing 1
Sord	sorbitol dehydrogenase
Sox12	SRY-box containing gene 12
Sox13	SRY-box containing gene 13
Sp3	trans-acting transcription factor 3
Spag6	sperm associated antigen 6
Spag9	sperm associated antigen 9
Sparc	secreted acidic cysteine rich glycoprotein
Spen	SPEN homolog, transcriptional regulator (Drosophila)
Sphk1	sphingosine kinase 1
Spop	speckle-type POZ protein
Spsb1	splA/ryanodine receptor domain and SOCS box containing 1
Spsb3	splA/ryanodine receptor domain and SOCS box containing 3
Sptlc2	serine palmitoyltransferase, long chain base subunit 2
Srebf2	sterol regulatory element binding factor 2
Srpk2	serine/arginine-rich protein specific kinase 2
Srrm2	serine/arginine repetitive matrix 2
Ssfa2	sperm specific antigen 2
Ssh1	slingshot homolog 1 (Drosophila)
Ssh3	slingshot homolog 3 (Drosophila)
Sspn	sarcospan
Ssr2	signal sequence receptor, beta
St18	suppression of tumorigenicity 18
St3gal4	ST3 beta-galactoside alpha-2,3-sialyltransferase 4
St5	suppression of tumorigenicity 5
St8sia1	ST8 alpha-N-acetyl-neuraminide alpha-2,8-sialyltransferase 1
Stard3	START domain containing 3
Stat6	signal transducer and activator of transcription 6
Stk10	serine/threonine kinase 10
Stk38l	serine/threonine kinase 38 like
Stom	stomatin
Suv39h2	suppressor of variegation 3-9 homolog 2 (Drosophila)
Syf2	SYF2 homolog, RNA splicing factor (S. cerevisiae)

Tacc1	transforming, acidic coiled-coil containing protein 1
Tada3l	transcriptional adaptor 3 (NGG1 homolog, yeast)-like
Tapbp	TAP binding protein
Tat	tyrosine aminotransferase
Tbc1d2	TBC1 domain family, member 2
Tbc1d5	TBC1 domain family, member 5
Tbl1x	transducin (beta)-like 1 X-linked
Tbx6	T-box 6
Tbxa2r	thromboxane A2 receptor
Tcf3	transcription factor 3
Tcf7l2	transcription factor 7-like 2, T-cell specific, HMG-box
Tcof1	Treacher Collins Franceschetti syndrome 1, homolog
Tdg	thymine DNA glycosylase
Tes	testis derived transcript
Tiparp	TCDD-inducible poly(ADP-ribose) polymerase
Tjp2	tight junction protein 2
Tle4	transducin-like enhancer of split 4, homolog of Drosophila E(spl)
Tll1	tolloid-like
Tmbim1	transmembrane BAX inhibitor motif containing 1
Tnfaip3	tumor necrosis factor, alpha-induced protein 3
Tnfaip8	tumor necrosis factor, alpha-induced protein 8
Tnfrsf1a	tumor necrosis factor receptor superfamily, member 1a
Tnfrsf9	tumor necrosis factor receptor superfamily, member 9
Tnfsf10	tumor necrosis factor (ligand) superfamily, member 10
Tnip1	TNFAIP3 interacting protein 1
Tnn	tenascin N
Tnpo1	transportin 1
Tnxb	tenascin XB
Tob2	transducer of ERBB2, 2
Tpd52l1	tumor protein D52-like 1
Tpmt	thiopurine methyltransferase
Tpte	transmembrane phosphatase with tensin homology
Tram1	translocating chain-associating membrane protein 1
Trim10	tripartite motif-containing 10
Trim14	tripartite motif-containing 14
Trim68	tripartite motif-containing 68
Triobp	TRIO and F-actin binding protein
Trps1	trichorhinophalangeal syndrome I (human)
Trpv1	transient receptor potential cation channel, subfamily V, member 1
Tspan2	tetraspanin 2
Tspan32	tetraspanin 32
Tspan5	tetraspanin 5
Tuba8	tubulin, alpha 8
Tulp3	tubby-like protein 3
U2af2	U2 small nuclear ribonucleoprotein auxiliary factor (U2AF) 2
Ubap2	ubiquitin-associated protein 2
Ube2l3	ubiquitin-conjugating enzyme E2L 3
Ucn	urocortin
Ugcg	UDP-glucose ceramide glucosyltransferase
Ulk2	Unc-51 like kinase 2 (C. elegans)
Usf2	upstream transcription factor 2
Ush1c	Usher syndrome 1C homolog (human)
Usp10	ubiquitin specific peptidase 10
Usp19	ubiquitin specific peptidase 19
Usp3	ubiquitin specific peptidase 3
Usp33	ubiquitin specific peptidase 33

Usp4	ubiquitin specific peptidase 4 (proto-oncogene)
Uvrag	UV radiation resistance associated gene
Vat1	vesicle amine transport protein 1 homolog (T californica)
Vegfb	vascular endothelial growth factor B
Vrk2	vaccinia related kinase 2
Wfdc2	WAP four-disulfide core domain 2
Whsc2	Wolf-Hirschhorn syndrome candidate 2 (human)
Wipi2	WD repeat domain, phosphoinositide interacting 2
Wnt7b	wingless-related MMTV integration site 7B
Wwox	WW domain-containing oxidoreductase
Wwp2	WW domain containing E3 ubiquitin protein ligase 2
Wwtr1	WW domain containing transcription regulator 1
Xbp1	X-box binding protein 1
Yipf2	Yip1 domain family, member 2
Zbtb20	zinc finger and BTB domain containing 20
Zc3hav1	zinc finger CCCH type, antiviral 1
Zfand3	zinc finger, AN1-type domain 3
Zfhx4	zinc finger homeodomain 4
Zfp36l1	zinc finger protein 36, C3H type-like 1
Zfp36l2	zinc finger protein 36, C3H type-like 2
Zmym2	zinc finger, MYM-type 2

Supplementary Table 8.
Intersection of the Recurrence Stromal Gene Set with Other Gene Sets.

Glycolysis (10 genes)

Aldh1b1	aldehyde dehydrogenase 1 family, member B1
Aldh3b1	aldehyde dehydrogenase 3 family, member B1
Aldob	aldolase B, fructose-bisphosphate
Dlat	dihydrolipoamide S-acetyltransferase (E2 component of pyruvate dehydrogenase complex)
G6pc	glucose-6-phosphatase, catalytic
Hk2	hexokinase 2
Ldhc	lactate dehydrogenase C
Pdha1	pyruvate dehydrogenase E1 alpha 1
Pdha2	pyruvate dehydrogenase E1 alpha 2
Pgk1	phosphoglycerate kinase 1

HIF Target Genes (108 genes)

Abcf2	ATP-binding cassette, sub-family F (GCN20), member 2
Actr1a	ARP1 actin-related protein 1 homolog A, centractin alpha (yeast)
Adat1	adenosine deaminase, tRNA-specific 1
Adm	adrenomedullin
Aff1	AF4/FMR2 family, member 1
Ak3l1	adenylate kinase 3-like 1
Aldh4a1	aldehyde dehydrogenase 4 family, member A1
Angptl6	angiopoietin-like 6
Arid1a	AT rich interactive domain 1A (SWI-like)
Ascc1	activating signal cointegrator 1 complex subunit 1
Asph	aspartate-beta-hydroxylase
Atf3	activating transcription factor 3
Atf7ip	activating transcription factor 7 interacting protein
Bat2	HLA-B associated transcript 2
Bcl11a	B-cell CLL/lymphoma 11A (zinc finger protein)
Bcl2l11	BCL2-like 11 (apoptosis facilitator)
Bcl9l	B-cell CLL/lymphoma 9-like
Bnip3	BCL2/adenovirus E1B interacting protein 3
Cabc1	chaperone, ABC1 activity of bc1 complex like (S. pombe)
Casc5	cancer susceptibility candidate 5
Ccnb1	cyclin B1
Cdc42ep4	CDC42 effector protein (Rho GTPase binding) 4
Clpb	ClpB caseinolytic peptidase B homolog (E. coli)
Cp	ceruloplasmin
Crh	corticotropin releasing hormone
Crispld1	cysteine-rich secretory protein LCCL domain containing 1
Dpysl2	dihydropyrimidinase-like 2
Edem1	ER degradation enhancer, mannosidase alpha-like 1
Efna1	ephrin A1
Elmo1	engulfment and cell motility 1, ced-12 homolog (C. elegans)
Epo	erythropoietin
Ets1	E26 avian leukemia oncogene 1, 5' domain
Evi1	ecotropic viral integration site 1
Fgd6	FYVE, RhoGEF and PH domain containing 6
Fgr	Gardner-Rasheed feline sarcoma viral (Fgr) oncogene homolog
Fn1	fibronectin 1
Fndc3b	fibronectin type III domain containing 3B
Fosl2	fos-like antigen 2
Gad1	glutamic acid decarboxylase 1

Ganab	alpha glucosidase 2 alpha neutral subunit
Gatad2b	GATA zinc finger domain containing 2B
Gmppb	GDP-mannose pyrophosphorylase B
Golga1	golgi autoantigen, golgin subfamily a, 1
Gopc	golgi associated PDZ and coiled-coil motif containing
Grk6	G protein-coupled receptor kinase 6
Hcfc1r1	host cell factor C1 regulator 1 (XPO1-dependent)
Hic2	hypermethylated in cancer 2
Hk2	hexokinase 2
Hsp90b1	heat shock protein 90, beta (Grp94), member 1
Hyou1	hypoxia up-regulated 1
Igf2	insulin-like growth factor 2
Igfbp2	insulin-like growth factor binding protein 2
Inha	inhibin alpha
Irf1	interferon regulatory factor 1
Jarid2	jumonji, AT rich interactive domain 2
Krt14	keratin 14
Krt18	keratin 18
Krt19	keratin 19
Lrrc20	leucine rich repeat containing 20
Mecp2	methyl CpG binding protein 2
Met	met proto-oncogene
Mfsd2	major facilitator superfamily domain containing 2
Mlxip	MLX interacting protein
Mrps12	mitochondrial ribosomal protein S12
Mylk	myosin, light polypeptide kinase
Nln	neurolysin (metallopeptidase M3 family)
Nos3	nitric oxide synthase 3, endothelial cell
Nr4a1	nuclear receptor subfamily 4, group A, member 1
Nrn1	neuritin 1
Ntrk2	neurotrophic tyrosine kinase, receptor, type 2
Nucb1	nucleobindin 1
Oxsr1	oxidative-stress responsive 1
P4ha2	procollagen-proline, 2-oxoglutarate 4-dioxygenase (proline 4-hydroxylase), alpha II polypeptide
P4hb	prolyl 4-hydroxylase, beta polypeptide
Papss2	3'-phosphoadenosine 5'-phosphosulfate synthase 2
Pdk1	pyruvate dehydrogenase kinase, isoenzyme 1
Per2	period homolog 2 (Drosophila)
Pfkfb3	6-phosphofructo-2-kinase/fructose-2,6-biphosphatase 3
Pgk1	---
Phf12	PHD finger protein 12
Pnma2	paraneoplastic antigen MA2
Ppp1r10	protein phosphatase 1, regulatory subunit 10
Ppp1r13l	protein phosphatase 1, regulatory (inhibitor) subunit 13 like
Pxn	paxillin
Rara	retinoic acid receptor, alpha
Rassf4	Ras association (RalGDS/AF-6) domain family member 4
Sdccag8	serologically defined colon cancer antigen 8
Sdk1	sidekick homolog 1 (chicken)
Sfxn3	sideroflexin 3
Slc16a1	solute carrier family 16 (monocarboxylic acid transporters), member 1
Slc25a28	solute carrier family 25, member 28
Slc2a1	solute carrier family 2 (facilitated glucose transporter), member 1
Slc35e1	solute carrier family 35, member E1
Slc6a6	solute carrier family 6 (neurotransmitter transporter, taurine), member 6

Slc7a6	solute carrier family 7 (cationic amino acid transporter, y+ system), member 6
Spast	spastin
Srrm2	serine/arginine repetitive matrix 2
St3gal5	ST3 beta-galactoside alpha-2,3-sialyltransferase 5
Stra13	stimulated by retinoic acid 13
Tert	telomerase reverse transcriptase
Tmcc3	transmembrane and coiled coil domains 3
Tmpo	thymopoietin
Tpcn1	two pore channel 1
Ubqln1	ubiquilin 1
Ugcgl1	UDP-glucose ceramide glucosyltransferase-like 1
Vldlr	very low density lipoprotein receptor
Wwox	WW domain-containing oxidoreductase
Wwp2	WW domain containing E3 ubiquitin protein ligase 2

Mitochondrial Associated Genes (120 genes)

Abat	4-aminobutyrate aminotransferase
Abcf2	ATP-binding cassette, sub-family F (GCN20), member 2
Acad9	acyl-Coenzyme A dehydrogenase family, member 9
Acadl	acyl-Coenzyme A dehydrogenase, long-chain
Acp6	acid phosphatase 6, lysophosphatidic
Acsl6	acyl-CoA synthetase long-chain family member 6
Acsm2	acyl-CoA synthetase medium-chain family member 2
Aldh4a1	aldehyde dehydrogenase 4 family, member A1
Aldh5a1	aldhehyde dehydrogenase family 5, subfamily A1
Apoa4	apolipoprotein A-IV
Asah2	N-acylsphingosine amidohydrolase 2
Bbc3	BCL2 binding component 3
Bckdha	branched chain ketoacid dehydrogenase E1, alpha polypeptide
Bcl2l1	BCL2-like 1
Bcl2l10	Bcl2-like 10
Bnip3	BCL2/adenovirus E1B interacting protein 3
Bzrap1	benzodiazapine receptor associated protein 1
Casp8	caspase 8
Casq1	calsequestrin 1
Cbx6	chromobox homolog 6
Cds2	CDP-diacylglycerol synthase (phosphatidate cytidylyltransferase) 2
Chdh	choline dehydrogenase
Clic4	chloride intracellular channel 4 (mitochondrial)
Cln3	ceroid lipofuscinosis, neuronal 3, juvenile (Batten, Spielmeyer-Vogt disease)
Cln8	ceroid-lipofuscinosis, neuronal 8
Clybl	citrate lyase beta like
Coq9	coenzyme Q9 homolog (yeast)
Cox15	COX15 homolog, cytochrome c oxidase assembly protein (yeast)
Cpt1b	carnitine palmitoyltransferase 1b, muscle
Cryab	crystallin, alpha B
Dhx32	DEAH (Asp-Glu-Ala-His) box polypeptide 32
Dnaja3	DnaJ (Hsp40) homolog, subfamily A, member 3
E2f1	E2F transcription factor 1
Epas1	endothelial PAS domain protein 1
Ercc2	excision repair cross-complementing rodent repair deficiency, complementation group 2
Esr2	estrogen receptor 2 (beta)
Foxred1	FAD-dependent oxidoreductase domain containing 1
Fpgs	folylpolyglutamyl synthetase
Frmd6	FERM domain containing 6

Ftmt	ferritin mitochondrial
Gclc	glutamate-cysteine ligase, catalytic subunit
Ghitm	growth hormone inducible transmembrane protein
Ghr	growth hormone receptor
Glyat	glycine-N-acyltransferase
Gpx2	glutathione peroxidase 2
Gpx5	glutathione peroxidase 5
Hkdc1	hexokinase domain containing 1
Hmgcs2	3-hydroxy-3-methylglutaryl-Coenzyme A synthase 2
Hmox2	heme oxygenase (decycling) 2
Hsd3b2	hydroxy-delta-5-steroid dehydrogenase, 3 beta- and steroid delta-isomerase 2
Hsdl2	hydroxysteroid dehydrogenase like 2
Hsh2d	hematopoietic SH2 domain containing
Idh3a	isocitrate dehydrogenase 3 (NAD+) alpha
Idh3b	isocitrate dehydrogenase 3 (NAD+) beta
Immt	inner membrane protein, mitochondrial
Kmo	kynurenine 3-monooxygenase (kynurenine 3-hydroxylase)
Ldhd	lactate dehydrogenase D
Letm1	leucine zipper-EF-hand containing transmembrane protein 1
Letm2	leucine zipper-EF-hand containing transmembrane protein 2
Maob	monoamine oxidase B
Mecp2	methyl CpG binding protein 2
Mfn1	mitofusin 1
Mlxip	MLX interacting protein
Mmab	methylmalonic aciduria (cobalamin deficiency) type B homolog (human)
Mobp	myelin-associated oligodendrocytic basic protein
Mpo	myeloperoxidase
Mrpl52	mitochondrial ribosomal protein L52
Mrpl55	mitochondrial ribosomal protein L55
Mrps12	mitochondrial ribosomal protein S12
Msra	methionine sulfoxide reductase A
Mtf1	metal response element binding transcription factor 1
Muc20	mucin 20
Mutyh	mutY homolog (E. coli)
Myc	myelocytomatosis oncogene
Nags	N-acetylglutamate synthase
Nol3	nucleolar protein 3 (apoptosis repressor with CARD domain)
Nos1	nitric oxide synthase 1, neuronal
Nt5m	5',3'-nucleotidase, mitochondrial
Nudt8	nudix (nucleoside diphosphate linked moiety X)-type motif 8
Ogdh	oxoglutarate dehydrogenase (lipoamide)
Olr1	oxidized low density lipoprotein (lectin-like) receptor 1
Oma1	OMA1 homolog, zinc metallopeptidase (S. cerevisiae)
Opa3	optic atrophy 3 (human)
Otc	ornithine transcarbamylase
Oxr1	oxidation resistance 1
Oxsr1	oxidative-stress responsive 1
Parl	presenilin associated, rhomboid-like
Pdha1	pyruvate dehydrogenase E1 alpha 1
Pdk1	pyruvate dehydrogenase kinase, isoenzyme 1
Pdk2	pyruvate dehydrogenase kinase, isoenzyme 2
Pemt	phosphatidylethanolamine N-methyltransferase
Pgs1	phosphatidylglycerophosphate synthase 1
Pnkp	polynucleotide kinase 3'- phosphatase
Polrmt	polymerase (RNA) mitochondrial (DNA directed)
Ppif	peptidylprolyl isomerase F (cyclophilin F)

Ppox	protoporphyrinogen oxidase
Prdx3	peroxiredoxin 3
Prep	prolyl endopeptidase
Psen1	presenilin 1
Ptcd2	pentatricopeptide repeat domain 2
Ptrf	polymerase I and transcript release factor
Scara3	scavenger receptor class A, member 3
Sdc1	syndecan 1
Sdhc	succinate dehydrogenase complex, subunit C, integral membrane protein
Sfxn3	sideroflexin 3
Sfxn5	sideroflexin 5
Shmt2	serine hydroxymethyltransferase 2 (mitochondrial)
Smcr7l	Smith-Magenis syndrome chromosome region, candidate 7-like (human)
Spg7	spastic paraplegia 7 homolog (human)
Stard3	START domain containing 3
Synj2	synaptojanin 2
Tes	testis derived transcript
Timm17b	translocase of inner mitochondrial membrane 17b
Tmem143	transmembrane protein 143
Trak1	trafficking protein, kinesin binding 1
Trpa1	transient receptor potential cation channel, subfamily A, member 1
Ucp1	uncoupling protein 1 (mitochondrial, proton carrier)
Ucp3	uncoupling protein 3 (mitochondrial, proton carrier)
Wnt2	wingless-related MMTV integration site 2
Wwox	WW domain-containing oxidoreductase

NFkB Target Genes (86 genes)

Acy3	aspartoacylase (aminoacylase) 3
Ahdc1	AT hook, DNA binding motif, containing 1
Anks3	ankyrin repeat and sterile alpha motif domain containing 3
Apln	apelin
Bmpr1a	bone morphogenetic protein receptor, type 1A
C8g	complement component 8, gamma polypeptide
Cbx8	chromobox homolog 8 (Drosophila Pc class)
Ccdc86	coiled-coil domain containing 86
Cenpi	centromere protein I
Chrnb1	cholinergic receptor, nicotinic, beta polypeptide 1 (muscle)
Cnn1	calponin 1
Cnp	2',3'-cyclic nucleotide 3' phosphodiesterase
Col3a1	collagen, type III, alpha 1
Ctf1	cardiotrophin 1
Cx3cl1	chemokine (C-X3-C motif) ligand 1
Dennd2c	DENN/MADD domain containing 2C
Dffb	DNA fragmentation factor, beta subunit
Ei24	etoposide induced 2.4 mRNA
Foxc2	forkhead box C2
Fzd1	frizzled homolog 1 (Drosophila)
Gclc	glutamate-cysteine ligase, catalytic subunit
Gdpd2	glycerophosphodiester phosphodiesterase domain containing 2
Gpr173	G-protein coupled receptor 173
Grwd1	glutamate-rich WD repeat containing 1
Gtse1	G two S phase expressed protein 1
Hmga1	high mobility group AT-hook 1
Il1rap	interleukin 1 receptor accessory protein
Irf1	interferon regulatory factor 1
Irx5	Iroquois related homeobox 5 (Drosophila)

Kbtbd2	kelch repeat and BTB (POZ) domain containing 2
Kcnk3	potassium channel, subfamily K, member 3
Kif23	kinesin family member 23
Klc4	kinesin light chain 4
Klf5	Kruppel-like factor 5
Krt19	keratin 19
Lhx2	LIM homeobox protein 2
Limd2	LIM domain containing 2
Lrrc8c	leucine rich repeat containing 8 family, member C
Luzp1	leucine zipper protein 1
Mafk	v-maf musculoaponeurotic fibrosarcoma oncogene family, protein K (avian)
Mdm2	transformed mouse 3T3 cell double minute 2
Msx2	homeobox, msh-like 2
Mybl2	myeloblastosis oncogene-like 2
Narg1	NMDA receptor-regulated gene 1
Ndrg4	N-myc downstream regulated gene 4
Nfatc4	nuclear factor of activated T-cells, cytoplasmic, calcineurin-dependent 4
Nr4a1	nuclear receptor subfamily 4, group A, member 1
Nrp2	neuropilin 2
Nucb1	nucleobindin 1
Nupr1	nuclear protein 1
Pak4	p21 (CDKN1A)-activated kinase 4
Pcdhb17	protocadherin beta 17
Pde4b	phosphodiesterase 4B, cAMP specific
Pdk2	pyruvate dehydrogenase kinase, isoenzyme 2
Phf21a	PHD finger protein 21A
Pld3	phospholipase D family, member 3
Polk	polymerase (DNA directed), kappa
Pom121	nuclear pore membrane protein 121
Ppp1r14a	protein phosphatase 1, regulatory (inhibitor) subunit 14A
Ptk2b	PTK2 protein tyrosine kinase 2 beta
Pxmp4	peroxisomal membrane protein 4
Rnpc3	RNA-binding region (RNP1, RRM) containing 3
Ropn1l	ropporin 1-like
Rras	Harvey rat sarcoma oncogene, subgroup R
Sac3d1	SAC3 domain containing 1
Selm	selenoprotein M
Sema3d	sema domain, immunoglobulin domain (Ig), short basic domain, secreted, (semaphorin) 3D
Sf1	splicing factor 1
Slc16a1	solute carrier family 16 (monocarboxylic acid transporters), member 1
Slc30a1	solute carrier family 30 (zinc transporter), member 1
Socs4	suppressor of cytokine signaling 4
Sp3	trans-acting transcription factor 3
Ssbp3	single-stranded DNA binding protein 3
Suv420h2	suppressor of variegation 4-20 homolog 2 (Drosophila)
Tlr2	toll-like receptor 2
Tmpo	thymopoietin
Tpp2	tripeptidyl peptidase II
Traf1	TNF receptor-associated factor 1
Tspyl1	testis-specific protein, Y-encoded-like 1
Tspyl4	TSPY-like 4
Tubg2	tubulin, gamma 2
Wbscr27	Williams Beuren syndrome chromosome region 27 (human)
Yipf2	Yip1 domain family, member 2
Zbtb41	zinc finger and BTB domain containing 41 homolog

Zc3h10	zinc finger CCCH type containing 10
Zswim3	zinc finger, SWIM domain containing 3

Response to Oxidative Stress (22 genes)

Apoa4	apolipoprotein A-IV
Cbx6	chromobox homolog 6
Cln8	ceroid-lipofuscinosis, neuronal 8
Cryab	crystallin, alpha B
Epas1	endothelial PAS domain protein 1
Ercc2	excision repair cross-complementing rodent repair deficiency, complementation group 2
Gclc	glutamate-cysteine ligase, catalytic subunit
Gpx2	glutathione peroxidase 2
Gpx5	glutathione peroxidase 5
Hmox2	heme oxygenase (decycling) 2
Mpo	myeloperoxidase
Msra	methionine sulfoxide reductase A
Mtf1	metal response element binding transcription factor 1
Mutyh	mutY homolog (E. coli)
Olr1	oxidized low density lipoprotein (lectin-like) receptor 1
Oxsr1	oxidative-stress responsive 1
Pnkp	polynucleotide kinase 3'- phosphatase
Prdx3	peroxiredoxin 3
Scara3	scavenger receptor class A, member 3
Sdc1	syndecan 1
Trpa1	transient receptor potential cation channel, subfamily A, member 1
Ucp3	uncoupling protein 3 (mitochondrial, proton carrier)

Alzheimers Disease Brain (338 genes)

Abcc10	ATP-binding cassette, sub-family C (CFTR/MRP), member 10
Abl1	c-abl oncogene 1, receptor tyrosine kinase
Acrv1	acrosomal vesicle protein 1
Acvr1b	activin A receptor, type 1B
Adarb2	adenosine deaminase, RNA-specific, B2
Adipoq	adiponectin, C1Q and collagen domain containing
Aff1	AF4/FMR2 family, member 1
Akap9	A kinase (PRKA) anchor protein (yotiao) 9
Aldh1a2	aldehyde dehydrogenase family 1, subfamily A2
Alms1	Alstrom syndrome 1 homolog (human)
Angpt1	angiopoietin 1
Angptl2	angiopoietin-like 2
Aoc2	amine oxidase, copper containing 2 (retina-specific)
Ap3s2	adaptor-related protein complex 3, sigma 2 subunit
Aqp1	aquaporin 1
Arhgap1	Rho GTPase activating protein 1
Arhgap6	Rho GTPase activating protein 6
Arhgef15	Rho guanine nucleotide exchange factor (GEF) 15
Arid1a	AT rich interactive domain 1A (SWI-like)
Ascl1	achaete-scute complex homolog 1 (Drosophila)
Atg3	autophagy-related 3 (yeast)
Atp6v0e	ATPase, H+ transporting, lysosomal V0 subunit E
Avpr1a	arginine vasopressin receptor 1A
Baz2b	bromodomain adjacent to zinc finger domain, 2B
Bcan	brevican
Bgn	biglycan
Bin1	bridging integrator 1

Bin3	bridging integrator 3
Bmp10	bone morphogenetic protein 10
Bmpr1a	bone morphogenetic protein receptor, type 1A
Brd2	bromodomain containing 2
Brd3	bromodomain containing 3
Brd4	bromodomain containing 4
Btrc	beta-transducin repeat containing protein
Cabc1	chaperone, ABC1 activity of bc1 complex like (S. pombe)
Cacna1a	calcium channel, voltage-dependent, P/Q type, alpha 1A subunit
Cacna1g	calcium channel, voltage-dependent, T type, alpha 1G subunit
Cant1	calcium activated nucleotidase 1
Casc3	cancer susceptibility candidate 3
Caskin2	CASK-interacting protein 2
Ccdc69	coiled-coil domain containing 69
Ccdc9	coiled-coil domain containing 9
Cchcr1	coiled-coil alpha-helical rod protein 1
Ccnd3	cyclin D3
Cd248	CD248 antigen, endosialin
Cd86	CD86 antigen
Cdc42ep4	CDC42 effector protein (Rho GTPase binding) 4
Cdh4	cadherin 4
Cdipt	CDP-diacylglycerol--inositol 3-phosphatidyltransferase (phosphatidylinositol synthase)
Ciz1	CDKN1A interacting zinc finger protein 1
Clec2d	C-type lectin domain family 2, member d
Cnot2	CCR4-NOT transcription complex, subunit 2
Cnp	2',3'-cyclic nucleotide 3' phosphodiesterase
Cog2	component of oligomeric golgi complex 2
Col4a5	collagen, type IV, alpha 5
Col8a2	collagen, type VIII, alpha 2
Cradd	CASP2 and RIPK1 domain containing adaptor with death domain
Csf1	colony stimulating factor 1 (macrophage)
Csk	c-src tyrosine kinase
Csnk1e	casein kinase 1, epsilon
Ctbp1	C-terminal binding protein 1
Ctbp2	C-terminal binding protein 2
Cxcl2	chemokine (C-X-C motif) ligand 2
Cyhr1	cysteine and histidine rich 1
Cyp11a1	cytochrome P450, family 11, subfamily a, polypeptide 1
Daam2	dishevelled associated activator of morphogenesis 2
Dag1	dystroglycan 1
Dcbld2	discoidin, CUB and LCCL domain containing 2
Dcp1a	DCP1 decapping enzyme homolog A (S. cerevisiae)
Ddx27	DEAD (Asp-Glu-Ala-Asp) box polypeptide 27
Dlx4	distal-less homeobox 4
Dmd	dystrophin, muscular dystrophy
Dnajb2	DnaJ (Hsp40) homolog, subfamily B, member 2
Dnajc4	DnaJ (Hsp40) homolog, subfamily C, member 4
Dpf3	D4, zinc and double PHD fingers, family 3
Drd2	dopamine receptor 2
Dtna	dystrobrevin alpha
Ecm2	extracellular matrix protein 2, female organ and adipocyte specific
Edar	ectodysplasin-A receptor
Ednra	endothelin receptor type A
Efna1	ephrin A1
Efs	embryonal Fyn-associated substrate

Egf	epidermal growth factor
Ehd1	EH-domain containing 1
Eif2c1	eukaryotic translation initiation factor 2C, 1
Elk1	ELK1, member of ETS oncogene family
Enah	enabled homolog (Drosophila)
Exph5	exophilin 5
Fads3	fatty acid desaturase 3
Fam107a	family with sequence similarity 107, member A
Fbxl7	F-box and leucine-rich repeat protein 7
Fbxo9	f-box protein 9
Fdps	farnesyl diphosphate synthetase
Fetub	fetuin beta
Fgfr1	fibroblast growth factor receptor 1
Fgfr3	fibroblast growth factor receptor 3
Fut2	fucosyltransferase 2
Fxr1	fragile X mental retardation gene 1, autosomal homolog
Fzr1	fizzy/cell division cycle 20 related 1 (Drosophila)
G6pc2	glucose-6-phosphatase, catalytic, 2
Gab2	growth factor receptor bound protein 2-associated protein 2
Gabrq	gamma-aminobutyric acid (GABA) A receptor, subunit theta
Gas2l1	growth arrest-specific 2 like 1
Gcc1	golgi coiled coil 1
Gclc	glutamate-cysteine ligase, catalytic subunit
Gjb1	gap junction protein, beta 1
Golga1	golgi autoantigen, golgin subfamily a, 1
Golga3	golgi autoantigen, golgin subfamily a, 3
Gpr4	G protein-coupled receptor 4
Gpr56	G protein-coupled receptor 56
Gpsm3	G-protein signalling modulator 3 (AGS3-like, C. elegans)
Grb10	growth factor receptor bound protein 10
Grk6	G protein-coupled receptor kinase 6
Gstm4	glutathione S-transferase, mu 4
Gstm5	glutathione S-transferase, mu 5
Gtf3c2	general transcription factor IIIC, polypeptide 2, beta
Gtse1	G two S phase expressed protein 1
H2afj	H2A histone family, member J
H2afx	H2A histone family, member X
Hapln2	hyaluronan and proteoglycan link protein 2
Hbegf	heparin-binding EGF-like growth factor
Heph	hephaestin
Hfe	hemochromatosis
Hip1r	huntingtin interacting protein 1 related
Hipk2	homeodomain interacting protein kinase 2
Homer3	homer homolog 3 (Drosophila)
Hs1bp3	HCLS1 binding protein 3
Hs2st1	heparan sulfate 2-O-sulfotransferase 1
Hspb8	heat shock protein 8
Id4	inhibitor of DNA binding 4
Igfbp5	insulin-like growth factor binding protein 5
Il13ra1	interleukin 13 receptor, alpha 1
Il1r1	interleukin 1 receptor, type I
Insig1	insulin induced gene 1
Irf7	interferon regulatory factor 7
Islr	immunoglobulin superfamily containing leucine-rich repeat
Itga7	integrin alpha 7
Itgb4	integrin beta 4

Itpk1	inositol 1,3,4-triphosphate 5/6 kinase
Itpkc	inositol 1,4,5-trisphosphate 3-kinase C
Jak3	Janus kinase 3
Jrk	jerky
Kbtbd2	kelch repeat and BTB (POZ) domain containing 2
Kcnj15	potassium inwardly-rectifying channel, subfamily J, member 15
Khsrp	KH-type splicing regulatory protein
Kif3b	kinesin family member 3B
Klf1	Kruppel-like factor 1 (erythroid)
Klf5	Kruppel-like factor 5
Lamp1	lysosomal-associated membrane protein 1
Lgals9	lectin, galactose binding, soluble 9
Lhx3	LIM homeobox protein 3
Limk2	LIM motif-containing protein kinase 2
Lmbr1l	limb region 1 like
Lrp10	low-density lipoprotein receptor-related protein 10
Lrp6	low density lipoprotein receptor-related protein 6
Lss	lanosterol synthase
Ltbp3	latent transforming growth factor beta binding protein 3
Lztr1	leucine-zipper-like transcriptional regulator, 1
Macf1	microtubule-actin crosslinking factor 1
Man2b1	mannosidase 2, alpha B1
Map2k7	mitogen-activated protein kinase kinase 7
Map3k11	mitogen-activated protein kinase kinase kinase 11
Map3k3	mitogen-activated protein kinase kinase kinase 3
Mapkapk2	MAP kinase-activated protein kinase 2
Masp1	mannan-binding lectin serine peptidase 1
Mast2	microtubule associated serine/threonine kinase 2
Max	Max protein
Mdm1	transformed mouse 3T3 cell double minute 1
Met	met proto-oncogene
Mgat1	mannoside acetylglucosaminyltransferase 1
Mknk2	MAP kinase-interacting serine/threonine kinase 2
Mmp12	matrix metallopeptidase 12
Mmp16	matrix metallopeptidase 16
Msx1	homeobox, msh-like 1
Mta1	metastasis associated 1
Mtf1	metal response element binding transcription factor 1
Mtmr11	myotubularin related protein 11
Mutyh	mutY homolog (E. coli)
Mvk	mevalonate kinase
Mx2	myxovirus (influenza virus) resistance 2
Mxd4	Max dimerization protein 4
Mycn	v-myc myelocytomatosis viral related oncogene, neuroblastoma derived (avian)
Myl4	myosin, light polypeptide 4
Mylk	myosin, light polypeptide kinase
Myo9b	myosin IXb
Myom1	myomesin 1
Myst4	MYST histone acetyltransferase monocytic leukemia 4
N4bp1	NEDD4 binding protein 1
Ncam1	neural cell adhesion molecule 1
Ncoa3	nuclear receptor coactivator 3
Ncstn	nicastrin
Nek7	NIMA (never in mitosis gene a)-related expressed kinase 7
Neu3	neuraminidase 3
Nfe2l1	nuclear factor, erythroid derived 2,-like 1

Nfx1	nuclear transcription factor, X-box binding 1
Nid1	nidogen 1
Notch4	Notch gene homolog 4 (Drosophila)
Npc1	Niemann Pick type C1
Nr1d1	nuclear receptor subfamily 1, group D, member 1
Nrp2	neuropilin 2
Nup43	nucleoporin 43
Ogdh	oxoglutarate dehydrogenase (lipoamide)
Pacsin2	protein kinase C and casein kinase substrate in neurons 2
Pak4	p21 (CDKN1A)-activated kinase 4
Pax6	paired box gene 6
Pbx2	pre B-cell leukemia transcription factor 2
Pcdh17	protocadherin 17
Pde4c	phosphodiesterase 4C, cAMP specific
Pde4d	phosphodiesterase 4D, cAMP specific
Pdgfb	platelet derived growth factor, B polypeptide
Pdlim4	PDZ and LIM domain 4
Pelp1	proline, glutamic acid and leucine rich protein 1
Per1	period homolog 1 (Drosophila)
Per2	period homolog 2 (Drosophila)
Pfkfb3	6-phosphofructo-2-kinase/fructose-2,6-biphosphatase 3
Phc2	polyhomeotic-like 2 (Drosophila)
Phf20	PHD finger protein 20
Phf21a	PHD finger protein 21A
Phlda2	pleckstrin homology-like domain, family A, member 2
Pla2g5	phospholipase A2, group V
Pla2g6	phospholipase A2, group VI
Plec1	plectin 1
Plod3	procollagen-lysine, 2-oxoglutarate 5-dioxygenase 3
Pml	promyelocytic leukemia
Polrmt	polymerase (RNA) mitochondrial (DNA directed)
Ppard	peroxisome proliferator activator receptor delta
Ppp1r13l	protein phosphatase 1, regulatory (inhibitor) subunit 13 like
Ppp1r1a	protein phosphatase 1, regulatory (inhibitor) subunit 1A
Prdx3	peroxiredoxin 3
Prlr	prolactin receptor
Prmt2	protein arginine N-methyltransferase 2
Pskh1	protein serine kinase H1
Ptcra	pre T-cell antigen receptor alpha
Ptdss2	phosphatidylserine synthase 2
Ptgis	prostaglandin I2 (prostacyclin) synthase
Ptp4a3	protein tyrosine phosphatase 4a3
Ptrf	polymerase I and transcript release factor
Pttg1ip	pituitary tumor-transforming 1 interacting protein
Pxn	paxillin
Rab22a	RAB22A, member RAS oncogene family
Ramp1	receptor (calcitonin) activity modifying protein 1
Rbbp5	retinoblastoma binding protein 5
Rbbp6	retinoblastoma binding protein 6
Rbms2	RNA binding motif, single stranded interacting protein 2
Rfng	RFNG O-fucosylpeptide 3-beta-N-acetylglucosaminyltransferase
Rfx4	regulatory factor X, 4 (influences HLA class II expression)
Rims2	regulating synaptic membrane exocytosis 2
Rin3	Ras and Rab interactor 3
Rnf24	ring finger protein 24
Rnf8	ring finger protein 8

Rnpepl1	arginyl aminopeptidase (aminopeptidase B)-like 1
Rps6ka1	ribosomal protein S6 kinase polypeptide 1
Runx2	runt related transcription factor 2
Rxrb	retinoid X receptor beta
Scrib	scribbled homolog (Drosophila)
Sema3c	sema domain, immunoglobulin domain (Ig), short basic domain, secreted, (semaphorin) 3C
Sema3f s	ema domain, immunoglobulin domain (Ig), short basic domain, secreted, (semaphorin) 3F
Sema4f	sema domain, immunoglobulin domain (Ig), TM domain, and short cytoplasmic domain
Senp3	SUMO/sentrin specific peptidase 3
Serpini2	serine (or cysteine) peptidase inhibitor, clade I, member 2
Sf3a3	splicing factor 3a, subunit 3
Sfrs16	splicing factor, arginine/serine-rich 16
Sfxn3	sideroflexin 3
Sgta	small glutamine-rich tetratricopeptide repeat (TPR)-containing, alpha
Sh3bp2	SH3-domain binding protein 2
Shmt2	serine hydroxymethyltransferase 2 (mitochondrial)
Sirt2	sirtuin 2 (silent mating type information regulation 2, homolog) 2 (S. cerevisiae)
Slc11a1	solute carrier family 11 (proton-coupled divalent metal ion transporters), member 1
Slc12a3	solute carrier family 12, member 3
Slc12a7	solute carrier family 12, member 7
Slc14a1	solute carrier family 14 (urea transporter), member 1
Slc1a7	solute carrier family 1 (glutamate transporter), member 7
Slc2a1	solute carrier family 2 (facilitated glucose transporter), member 1
Slc35a3	solute carrier family 35 (UDP-N-acetylglucosamine (UDP-GlcNAc) transporter), member 3
Slc4a4	solute carrier family 4 (anion exchanger), member 4
Slc7a5	solute carrier family 7 (cationic amino acid transporter, y+ system), member 5
Slco3a1	solute carrier organic anion transporter family, member 3a1
Smarcc1	SWI/SNF related, matrix associated, actin dependent regulator of chromatin, subfamily c, member 1
Smarcd1	SWI/SNF related, matrix associated, actin dependent regulator of chromatin, subfamily d, member 1
Smc4	structural maintenance of chromosomes 4
Snta1	syntrophin, acidic 1
Socs3	suppressor of cytokine signaling 3
Sorbs1	sorbin and SH3 domain containing 1
Sox13	SRY-box containing gene 13
Sp3	trans-acting transcription factor 3
Spag9	sperm associated antigen 9
Spen	SPEN homolog, transcriptional regulator (Drosophila)
Sphk1	sphingosine kinase 1
Srrm2	serine/arginine repetitive matrix 2
Ssh1	slingshot homolog 1 (Drosophila)
Ssh3	slingshot homolog 3 (Drosophila)
St18	suppression of tumorigenicity 18
Stard3	START domain containing 3
Stk38l	serine/threonine kinase 38 like
Tada3l	transcriptional adaptor 3 (NGG1 homolog, yeast)-like
Taf5	TAF5 RNA polymerase II, TATA box binding protein (TBP)-associated factor
Tapbp	TAP binding protein
Tat	tyrosine aminotransferase
Tbx6	T-box 6
Tcf7l2	transcription factor 7-like 2, T-cell specific, HMG-box

Tcn2	transcobalamin 2
Tcof1	Treacher Collins Franceschetti syndrome 1, homolog
Tdg	thymine DNA glycosylase
Tes	testis derived transcript
Tex264	testis expressed gene 264
Tjp2	tight junction protein 2
Tle4	transducin-like enhancer of split 4, homolog of Drosophila E(spl)
Tll1	tolloid-like
Tnfrsf11b	tumor necrosis factor receptor superfamily, member 11b (osteoprotegerin)
Tnfrsf9	tumor necrosis factor receptor superfamily, member 9
Tnxb	tenascin XB
Tob2	transducer of ERBB2, 2
Tpmt	thiopurine methyltransferase
Trib3	tribbles homolog 3 (Drosophila)
Trim10	tripartite motif-containing 10
Trim14	tripartite motif-containing 14
Triobp	TRIO and F-actin binding protein
Trpv1	transient receptor potential cation channel, subfamily V, member 1
Tsc22d4	TSC22 domain family, member 4
Tspan32	tetraspanin 32
Tspan5	tetraspanin 5
U2af2	U2 small nuclear ribonucleoprotein auxiliary factor (U2AF) 2
Ube2c	ubiquitin-conjugating enzyme E2C
Ucn	urocortin
Usf2	upstream transcription factor 2
Ush1c	Usher syndrome 1C homolog (human)
Usp19	ubiquitin specific peptidase 19
Usp22	ubiquitin specific peptidase 22
Usp4	ubiquitin specific peptidase 4 (proto-oncogene)
Utp14a	UTP14, U3 small nucleolar ribonucleoprotein, homolog A (yeast)
Vat1	vesicle amine transport protein 1 homolog (T californica)
Whsc2	Wolf-Hirschhorn syndrome candidate 2 (human)
Wipi2	WD repeat domain, phosphoinositide interacting 2
Wwox	WW domain-containing oxidoreductase
Wwp2	WW domain containing E3 ubiquitin protein ligase 2
Wwtr1	WW domain containing transcription regulator 1
Yipf2	Yip1 domain family, member 2
Zfp36l1	zinc finger protein 36, C3H type-like 1

Supplementary Table 9.
Intersection of the LN Metastasis Stromal Gene Set with Other Gene Sets.

Glycolysis (7 genes)

Acss1	acyl-CoA synthetase short-chain family member 1
Aldh1b1	aldehyde dehydrogenase 1 family, member B1
Aldoa	aldolase A, fructose-bisphosphate
Eno3	enolase 3, beta muscle
Pfkl	phosphofructokinase, liver, B-type
Pklr	pyruvate kinase liver and red blood cell
Pkm2	pyruvate kinase, muscle

HIF Target Genes (42 genes)

Acaca	acetyl-Coenzyme A carboxylase alpha
Aldoa	aldolase A, fructose-bisphosphate
Arid1a	AT rich interactive domain 1A (SWI-like)
Bcl11a	B-cell CLL/lymphoma 11A (zinc finger protein)
C1galt1c1	C1GALT1-specific chaperone 1
Cdca3	cell division cycle associated 3
Clk3	CDC-like kinase 3
Cox17	cytochrome c oxidase, subunit XVII assembly protein homolog (yeast)
Cxcl12	chemokine (C-X-C motif) ligand 12
Cyr61	cysteine rich protein 61
Efna1	ephrin A1
Elavl1	ELAV (embryonic lethal, abnormal vision, Drosophila)-like 1 (Hu antigen R)
Elf1	E74-like factor 1
Ergic2	ERGIC and golgi 2
Fgr	Gardner-Rasheed feline sarcoma viral (Fgr) oncogene homolog
Fus	fusion, derived from t(12;16) malignant liposarcoma (human)
Gpx3	glutathione peroxidase 3
Hcfc1r1	host cell factor C1 regulator 1 (XPO1-dependent)
Hdhd1a	haloacid dehalogenase-like hydrolase domain containing 1A
Hic2	hypermethylated in cancer 2
Igf2	insulin-like growth factor 2
Igfbp1	insulin-like growth factor binding protein 1
Igfbp2	insulin-like growth factor binding protein 2
Jarid2	jumonji, AT rich interactive domain 2
Lcorl	ligand dependent nuclear receptor corepressor-like
Lsp1	lymphocyte specific 1
Mov10	Moloney leukemia virus 10
Mrpl33	mitochondrial ribosomal protein L33
Mrpl4	mitochondrial ribosomal protein L4
Nup93	nucleoporin 93
P4ha2	procollagen-proline, 2-oxoglutarate 4-dioxygenase (proline 4-hydroxylase), alpha II polypeptide
Pfkfb3	6-phosphofructo-2-kinase/fructose-2,6-biphosphatase 3
Pfkl	phosphofructokinase, liver, B-type
Pkm2	pyruvate kinase, muscle
Rsl1d1	ribosomal L1 domain containing 1
Sdk1	sidekick homolog 1 (chicken)
Sox4	SRY-box containing gene 4
Tert	telomerase reverse transcriptase
Tff3	trefoil factor 3, intestinal
Tpcn1	two pore channel 1
Vcp	---
Wdr33	WD repeat domain 33

<u>Mitochondrial Associated Genes (68 genes)</u>

Aadat	aminoadipate aminotransferase
Acadvl	acyl-Coenzyme A dehydrogenase, very long chain
Acsl4	acyl-CoA synthetase long-chain family member 4
Alas2	aminolevulinic acid synthase 2, erythroid
Aldh5a1	aldhehyde dehydrogenase family 5, subfamily A1
Aldoa	aldolase A, fructose-bisphosphate
Areg	amphiregulin
Atp5s	ATP synthase, H+ transporting, mitochondrial F0 complex, subunit s
Atrn	attractin
Bax	BCL2-associated X protein
Bbc3	BCL2 binding component 3
Bcat2	branched chain aminotransferase 2, mitochondrial
Bckdk	branched chain ketoacid dehydrogenase kinase
Bcs1l	BCS1-like (yeast)
Bdh2	3-hydroxybutyrate dehydrogenase, type 2
Cebpa	CCAAT/enhancer binding protein (C/EBP), alpha
Clpp	caseinolytic peptidase, ATP-dependent, proteolytic subunit homolog (E. coli)
Clpx	caseinolytic peptidase X (E.coli)
Coq3	coenzyme Q3 homolog, methyltransferase (yeast)
Coq7	demethyl-Q 7
Cox17	cytochrome c oxidase, subunit XVII assembly protein homolog (yeast)
Cox18	---
Cpt1c	carnitine palmitoyltransferase 1c
Dmgdh	dimethylglycine dehydrogenase precursor
Efhd1	EF hand domain containing 1
Etfa	electron transferring flavoprotein, alpha polypeptide
Fahd1	fumarylacetoacetate hydrolase domain containing 1
Fdxr	ferredoxin reductase
Fen1	flap structure specific endonuclease 1
Gpx3	glutathione peroxidase 3
Hsp90aa1	heat shock protein 90, alpha (cytosolic), class A member 1
Idh3b	isocitrate dehydrogenase 3 (NAD+) beta
Immt	inner membrane protein, mitochondrial
Kars	lysyl-tRNA synthetase
Lrpprc	leucine-rich PPR-motif containing
Mgst1	microsomal glutathione S-transferase 1
Mosc2	MOCO sulphurase C-terminal domain containing 2
Mrpl12	mitochondrial ribosomal protein L12
Mrpl55	mitochondrial ribosomal protein L55
Mtch1	mitochondrial carrier homolog 1 (C. elegans)
Mtch2	mitochondrial carrier homolog 2 (C. elegans)
Ndufa13	NADH dehydrogenase (ubiquinone) 1 alpha subcomplex, 13
Ndufs2	NADH dehydrogenase (ubiquinone) Fe-S protein 2
Nudt9	nudix (nucleoside diphosphate linked moiety X)-type motif 9
Ogdh	oxoglutarate dehydrogenase (lipoamide)
Ogg1	8-oxoguanine DNA-glycosylase 1
Pemt	phosphatidylethanolamine N-methyltransferase
Pet112l	PET112-like (yeast)
Phb	prohibitin
Phb2	prohibitin 2
Pink1	PTEN induced putative kinase 1
Plcg1	phospholipase C, gamma 1
Pmpca	peptidase (mitochondrial processing) alpha
Prdx2	peroxiredoxin 2

Prdx6	peroxiredoxin 6
Ptcd2	pentatricopeptide repeat domain 2
Sardh	sarcosine dehydrogenase
Sdha	succinate dehydrogenase complex, subunit A, flavoprotein (Fp)
Sfn	stratifin
Spg7	spastic paraplegia 7 homolog (human)
Stk25	serine/threonine kinase 25 (yeast)
Tfb1m	transcription factor B1, mitochondrial
Timm8b	translocase of inner mitochondrial membrane 8 homolog b (yeast)
Tor1a	torsin family 1, member A (torsin A)
Trnt1	tRNA nucleotidyl transferase, CCA-adding, 1
Tsfm	Ts translation elongation factor, mitochondrial
Tspo	translocator protein
Wasf1	WASP family 1

NFkB Target Genes (32 genes)

Anks3	ankyrin repeat and sterile alpha motif domain containing 3
Anln	anillin, actin binding protein
Apod	apolipoprotein D
Bdh2	3-hydroxybutyrate dehydrogenase, type 2
Ccdc86	coiled-coil domain containing 86
Col6a1	collagen, type VI, alpha 1
Eif5b	eukaryotic translation initiation factor 5B
Ergic2	ERGIC and golgi 2
Gpr153	G protein-coupled receptor 153
Igsf9	immunoglobulin superfamily, member 9
Klhl22	kelch-like 22 (Drosophila)
Limd2	LIM domain containing 2
Lrdd	leucine-rich and death domain containing
Lysmd1	LysM, putative peptidoglycan-binding, domain containing 1
Mvp	major vault protein
Mybbp1a	MYB binding protein (P160) 1a
Mycbp	c-myc binding protein
Nupr1	nuclear protein 1
Otub2	OTU domain, ubiquitin aldehyde binding 2
Pld3	phospholipase D family, member 3
Pom121	nuclear pore membrane protein 121
Pros1	protein S (alpha)
Ptk2b	PTK2 protein tyrosine kinase 2 beta
Rps6ka3	ribosomal protein S6 kinase polypeptide 3
Sema3d	sema domain, immunoglobulin domain (Ig), short basic domain, secreted, (semaphorin) 3D
Sp3	trans-acting transcription factor 3
Tmem160	transmembrane protein 160
Tspo	translocator protein
Ttc30b	tetratricopeptide repeat domain 30B
Tubg2	tubulin, gamma 2
Tyk2	tyrosine kinase 2
Wdr77	WD repeat domain 77

Response to Oxidative Stress (9 genes)

Areg	amphiregulin
Atrn	attractin
Gpx3	glutathione peroxidase 3
Ndufs2	NADH dehydrogenase (ubiquinone) Fe-S protein 2
Plcg1	phospholipase C, gamma 1

Prdx2	peroxiredoxin 2
Prdx6	peroxiredoxin 6
Stk25	serine/threonine kinase 25 (yeast)
Tor1a	torsin family 1, member A (torsin A)

Alzheimers Disease Brain (145 genes)

Abca2	ATP-binding cassette, sub-family A (ABC1), member 2
Abl1	c-abl oncogene 1, receptor tyrosine kinase
Adarb2	adenosine deaminase, RNA-specific, B2
Alms1	Alstrom syndrome 1 homolog (human)
App	amyloid beta (A4) precursor protein
Arhgap1	Rho GTPase activating protein 1
Arid1a	AT rich interactive domain 1A (SWI-like)
Atg4b	autophagy-related 4B (yeast)
Banp	BTG3 associated nuclear protein
Bcan	brevican
Bcat2	branched chain aminotransferase 2, mitochondrial
Bhmt2	betaine-homocysteine methyltransferase 2
Cacna1a	calcium channel, voltage-dependent, P/Q type, alpha 1A subunit
Cacna1g	calcium channel, voltage-dependent, T type, alpha 1G subunit
Caskin2	CASK-interacting protein 2
Ccdc59	coiled-coil domain containing 59
Cdh4	cadherin 4
Cebpa	CCAAT/enhancer binding protein (C/EBP), alpha
Clec2d	C-type lectin domain family 2, member d
Clic1	chloride intracellular channel 1
Cnot2	CCR4-NOT transcription complex, subunit 2
Coq7	demethyl-Q 7
Csnk1e	casein kinase 1, epsilon
Ctbp1	C-terminal binding protein 1
Ddx27	DEAD (Asp-Glu-Ala-Asp) box polypeptide 27
Ddx39	DEAD (Asp-Glu-Ala-Asp) box polypeptide 39
Dlg5	discs, large homolog 5 (Drosophila)
Donson	downstream neighbor of SON
Efna1	ephrin A1
Egf	epidermal growth factor
Eif4b	eukaryotic translation initiation factor 4B
Eif5b	eukaryotic translation initiation factor 5B
Exosc8	exosome component 8
Fdps	farnesyl diphosphate synthetase
Fez2	fasciculation and elongation protein zeta 2 (zygin II)
Fgfr1	fibroblast growth factor receptor 1
Gamt	guanidinoacetate methyltransferase
Gfap	glial fibrillary acidic protein
Gga1	golgi associated, gamma adaptin ear containing, ARF binding protein 1
Ggps1	geranylgeranyl diphosphate synthase 1
Gjb1	gap junction protein, beta 1
Gnaz	guanine nucleotide binding protein, alpha z subunit
Gpr56	G protein-coupled receptor 56
Gstm5	glutathione S-transferase, mu 5
H2afx	H2A histone family, member X
Hbegf	heparin-binding EGF-like growth factor
Hdgf	hepatoma-derived growth factor
Hip1r	huntingtin interacting protein 1 related
Hist1h4h	histone cluster 1, H4h
Hmg20b	high mobility group 20 B

Hmgn3	high mobility group nucleosomal binding domain 3
Homer3	homer homolog 3 (Drosophila)
Hs1bp3	HCLS1 binding protein 3
Igfbp5	insulin-like growth factor binding protein 5
Irak1	interleukin-1 receptor-associated kinase 1
Irf7	interferon regulatory factor 7
Jrk	jerky
Khsrp	KH-type splicing regulatory protein
Klf1	Kruppel-like factor 1 (erythroid)
Larp7	La ribonucleoprotein domain family, member 7
Lgals9	lectin, galactose binding, soluble 9
Lrdd	leucine-rich and death domain containing
Lrp10	low-density lipoprotein receptor-related protein 10
Lrp6	low density lipoprotein receptor-related protein 6
Lsm14a	LSM14 homolog A (SCD6, S. cerevisiae)
Ltbp2	latent transforming growth factor beta binding protein 2
Map2k7	mitogen-activated protein kinase kinase 7
Map3k11	mitogen-activated protein kinase kinase kinase 11
Masp1	mannan-binding lectin serine peptidase 1
Mc1r	melanocortin 1 receptor
Mcm7	minichromosome maintenance deficient 7 (S. cerevisiae)
Mpst	mercaptopyruvate sulfurtransferase
Mta1	metastasis associated 1
Mvk	mevalonate kinase
Mybpc1	myosin binding protein C, slow-type
Mycn	v-myc myelocytomatosis viral related oncogene, neuroblastoma derived (avian)
Mzf1	myeloid zinc finger 1
Nde1	nuclear distribution gene E homolog 1 (A nidulans)
Nosip	nitric oxide synthase interacting protein
Npas3	neuronal PAS domain protein 3
Nr2f1	nuclear receptor subfamily 2, group F, member 1
Nucks1	nuclear casein kinase and cyclin-dependent kinase substrate 1
Ogdh	oxoglutarate dehydrogenase (lipoamide)
Ogg1	8-oxoguanine DNA-glycosylase 1
Pabpc1	poly(A) binding protein, cytoplasmic 1
Pabpc3	poly(A) binding protein, cytoplasmic 3
Pdlim4	PDZ and LIM domain 4
Pfkfb3	6-phosphofructo-2-kinase/fructose-2,6-biphosphatase 3
Phf10	PHD finger protein 10
Phf20	PHD finger protein 20
Pias4	protein inhibitor of activated STAT 4
Plag1	pleiomorphic adenoma gene 1
Plec1	plectin 1
Pml	promyelocytic leukemia
Ppp1r1a	protein phosphatase 1, regulatory (inhibitor) subunit 1A
Prdx6	peroxiredoxin 6
Prkx	protein kinase, X-linked
Prpf40a	PRP40 pre-mRNA processing factor 40 homolog A (yeast)
Pskh1	protein serine kinase H1
Psmf1	proteasome (prosome, macropain) inhibitor subunit 1
Ptdss2	phosphatidylserine synthase 2
Ptprk	protein tyrosine phosphatase, receptor type, K
Rab13	RAB13, member RAS oncogene family
Rbm10	RNA binding motif protein 10
Rbmx2	RNA binding motif protein, X-linked 2
Rfng	RFNG O-fucosylpeptide 3-beta-N-acetylglucosaminyltransferase

Riok3	RIO kinase 3 (yeast)
Rnaseh2a	ribonuclease H2, large subunit
Rnpepl1	arginyl aminopeptidase (aminopeptidase B)-like 1
Rpl13a ---	
Rrbp1	ribosome binding protein 1
Sall2	sal-like 2 (Drosophila)
Scarb1	scavenger receptor class B, member 1
Scrib	scribbled homolog (Drosophila)
Sema3b	sema domain, immunoglobulin domain (Ig), short basic domain, secreted, (semaphorin) 3B
Sema3f	sema domain, immunoglobulin domain (Ig), short basic domain, secreted, (semaphorin) 3F
Serpini2	serine (or cysteine) peptidase inhibitor, clade I, member 2
Sfn	stratifin
Sfrp1	secreted frizzled-related protein 1
Sftpc	surfactant associated protein C
Sirt7	sirtuin 7 (silent mating type information regulation 2, homolog) 7 (S. cerevisiae)
Slc11a1	solute carrier family 11 (proton-coupled divalent metal ion transporters), member 1
Smad2	MAD homolog 2 (Drosophila)
Smarcc2	SWI/SNF related, matrix associated, actin dependent regulator of chromatin, subfamily c, member 2
Smc1a	structural maintenance of chromosomes 1A
Sord	sorbitol dehydrogenase
Sp3	trans-acting transcription factor 3
St6galnac4	ST6 (alpha-N-acetyl-neuraminyl-2,3-beta-galactosyl-1,3)-N-acetylgalactosaminide alpha-2,6-sialyltransferase 4
Tat	tyrosine aminotransferase
Tcf7	transcription factor 7, T-cell specific
Tcof1	Treacher Collins Franceschetti syndrome 1, homolog
Tex264	testis expressed gene 264
Tle3	transducin-like enhancer of split 3, homolog of Drosophila E(spl)
Trim10	tripartite motif-containing 10
Trim14	tripartite motif-containing 14
Trpv1	transient receptor potential cation channel, subfamily V, member 1
Tsc22d4	TSC22 domain family, member 4
Tspo	translocator protein
U2af2	U2 small nuclear ribonucleoprotein auxiliary factor (U2AF) 2
Upf1	UPF1 regulator of nonsense transcripts homolog (yeast)
Usp13	ubiquitin specific peptidase 13 (isopeptidase T-3)
Whsc2	Wolf-Hirschhorn syndrome candidate 2 (human)
Wipi2	WD repeat domain, phosphoinositide interacting 2
Zfp36l2	zinc finger protein 36, C3H type-like 2
Zic1	zinc finger protein of the cerebellum 1

Supplementary Table 10.
Intersection of the Cav-1 (-/-) Stromal Gene List with Genes that are Up-regulated in ER (-) Breast Cancer (FC >1.5)

Gene Symbol and Description		Fold Change (KO/WT)	Accession Number	P-value
**Acot7**	**acyl-CoA thioesterase 7**	**1.76**	**NM_133348**	**0.04**
**Acsl4**	**acyl-CoA synthetase long-chain family member 4**	**1.86**	**NM_207625**	**0.01**
**Actl6a**	**actin-like 6A**	**1.80**	**NM_019673**	**0.002**
Adam8	a disintegrin and metallopeptidase domain 8	1.62	NM_007403	0.03
**Aif1**	**allograft inflammatory factor 1**	**2.93**	**NM_019467**	**0.04**
Aim1	absent in melanoma 1	1.71	NM_172393	0.0005
Anxa3	annexin A3	1.65	NM_013470	0.03
Anxa8	annexin A8	1.59	NM_013473	0.04
Arhgef19	Rho guanine nucleotide exchange factor (GEF) 19	2.26	NM_172520	0.02
Arl4c	ADP-ribosylation factor-like 4C	2.13	BC055769	0.04
Arrdc2	arrestin domain containing 2	2.04	NM_027560	0.05
Atp11b	ATPase, class VI, type 11B	2.81	NM_029570	0.0004
Bace2	beta-site APP-cleaving enzyme 2	1.52	NM_019517	0.05
Bcl11a	B-cell CLL/lymphoma 11A (zinc finger protein)	1.98	NM_016707	0.009
Bnip2	BCL2/adenovirus E1B interacting protein 2	1.51	NM_016787	0.02
Bnip3	BCL2/adenovirus E1B interacting protein 3	1.75	NM_009760	0.02
Bop1	block of proliferation 1	1.82	NM_013481	0.03
**C3**	**complement component 3**	**4.35**	**NM_009778**	**0.004**
**Capg**	**capping protein (actin filament), gelsolin-like**	**1.90**	**NM_007599**	**0.02**
Cbr1	carbonyl reductase 1	2.43	NM_007620	0.01
Ccdc109b	coiled-coil domain containing 109B	1.56	NM_025779	0.01
**Ccl5**	**chemokine (C-C motif) ligand 5**	**4.64**	**NM_013653**	**0.05**
Ccna2	cyclin A2	1.60	NM_009828	0.05
Cct5	chaperonin containing Tcp1, subunit 5 (epsilon)	1.93	NM_007637	0.005
Cd52	CD52 antigen	1.80	NM_013706	0.02
Cenpa	centromere protein A	1.54	NM_007681	0.008
Cep192	centrosomal protein 192	1.82	BC064462	0.04
Chi3l1	chitinase 3-like 1	1.65	NM_007695	0.04
**Col9a3**	**collagen, type IX, alpha 3**	**1.73**	**NM_009936**	**0.04**
Copg	coatomer protein complex, subunit gamma	1.84	NM_017477	0.006
Creb3l2	cAMP responsive element binding protein 3-like 2	1.56	NM_178661	0.02
**Crlf3**	**cytokine receptor-like factor 3**	**2.15**	**NM_018776**	**0.006**
Csrp2	cysteine and glycine-rich protein 2	2.10	NM_007792	0.02
Cxadr	coxsackie virus and adenovirus receptor	2.20	NM_009988	0.008
Cyba	cytochrome b-245, alpha polypeptide	2.68	NM_007806	0.03
Dapk1	death associated protein kinase 1	2.60	NM_029653	0.006
Dbn1	drebrin 1	2.23	NM_019813	0.04
**Ddit3**	**DNA-damage inducible transcript 3**	**2.12**	**NM_007837**	**0.009**
Ddx11	DEAD/H (Asp-Glu-Ala-Asp/His) box polypeptide 11 (CHL1-like helicase homolog, S. cerevisiae)	2.12	NM_001003919	0.03

**Dnmt3b**	**DNA methyltransferase 3B**	**1.94**	**NM_001003961**	**0.007**
Dsc3	desmocollin 3	2.61	NM_007882	0.03
Dtx2	deltex 2 homolog (Drosophila)	1.81	NM_023742	0.04
Ehbp1	EH domain binding protein 1	1.68	NM_153078	0.04
Eif5a	eukaryotic translation initiation factor 5A	1.58	NM_181582	0.02
**Eno1**	**enolase 1, alpha non-neuron**	**2.11**	**NM_023119**	**0.0002**
Fabp7	fatty acid binding protein 7, brain	2.21	NM_021272	0.04
Fam107a	family with sequence similarity 107, member A	1.77	NM_183187	0.05
Fanca	Fanconi anemia, complementation group A	1.91	NM_016925	0.05
Fancl	Fanconi anemia, complementation group L	1.99	NM_025923	0.04
Fcho1	FCH domain only 1	1.88	NM_028715	0.01
Foxc1	forkhead box C1	2.56	NM_008592	0.03
Foxm1	forkhead box M1	1.62	NM_008021	0.004
Fscn1	fascin homolog 1, actin bundling protein (Strongylocentrotus purpuratus)	2.13	NM_007984	0.02
Fus	fusion, derived from t(12;16) malignant liposarcoma (human)	1.79	NM_139149	0.05
Galnt3	UDP-N-acetyl-alpha-D-galactosamine:polypeptide N-acetylgalactosaminyltransferase 3	1.61	NM_015736	0.05
**Gapdh**	**---**	**1.58**	**NM_008084**	**0.04**
Gars	glycyl-tRNA synthetase	1.55	NM_180678	0.02
Gata6	GATA binding protein 6	2.11	NM_010258	0.05
Gatad2a	GATA zinc finger domain containing 2A	1.70	NM_145596	0.009
Gdf5	growth differentiation factor 5	1.65	NM_008109	0.02
Gimap5	GTPase, IMAP family member 5	1.66	NM_175035	0.05
Gmnn	geminin	2.22	NM_020567	0.03
Gnb4	guanine nucleotide binding protein (G protein), beta 4	1.94	NM_013531	0.01
Gpsm2	G-protein signalling modulator 2 (AGS3-like, C. elegans)	1.54	NM_029522	0.03
Grhl1	grainyhead-like 1 (Drosophila)	1.82	NM_145890	0.03
Gypc	glycophorin C	1.58	NM_001048207	0.02
Hck	hemopoietic cell kinase	1.55	NM_010407	0.03
Hdgf	hepatoma-derived growth factor	1.75	NM_008231	0.05
Heatr1	HEAT repeat containing 1	1.63	NM_144835	0.001
Hmga1	high mobility group AT-hook 1	1.95	NM_016660	0.02
Hmgn3	high mobility group nucleosomal binding domain 3	1.62	NM_026122	0.03
Hn1	hematological and neurological expressed sequence 1	1.86	NM_008258	0.007
Hrk	harakiri, BCL2 interacting protein (contains only BH3 domain)	1.96	NM_007545	0.04
**Hyou1**	**hypoxia up-regulated 1**	**1.57**	**NM_021395**	**0.02**
Icam1	intercellular adhesion molecule 1	1.87	NM_010493	0.007
**Ifngr1**	**interferon gamma receptor 1**	**1.72**	**NM_010511**	**0.02**
**Il10ra**	**interleukin 10 receptor, alpha**	**2.38**	**NM_008348**	**0.004**
**Irak1**	**interleukin-1 receptor-associated kinase 1**	**1.77**	**NM_008363**	**0.03**
**Irf5**	**interferon regulatory factor 5**	**1.66**	**NM_012057**	**0.002**
**Isg20**	**interferon-stimulated protein**	**2.08**	**NM_020583**	**0.02**
Itgb2	integrin beta 2	2.33	NM_008404	0.02
Kcnk5	potassium channel, subfamily K, member 5	1.52	NM_021542	0.02
Kctd9	potassium channel tetramerisation domain containing 9	1.94	NM_001111028	0.001
Kif20a	kinesin family member 20A	1.60	NM_009004	0.05

Kif21b	kinesin family member 21B	1.84	NM_001039472	0.04
Kif3c	kinesin family member 3C	1.96	NM_008445	0.02
Laptm5	lysosomal-associated protein transmembrane 5	1.93	NM_010686	0.02
**Ldhb**	**lactate dehydrogenase B**	**2.44**	**NM_008492**	**0.02**
Lgals9	lectin, galactose binding, soluble 9	1.83	NM_010708	0.03
Limk1	LIM-domain containing, protein kinase	1.68	NM_010717	0.01
Limk2	LIM motif-containing protein kinase 2	1.70	NM_173053	0.04
Lpxn	leupaxin	1.83	NM_134152	0.02
Lrp8	low density lipoprotein receptor-related protein 8, apolipoprotein e receptor	2.29	NM_053073	0.003
Ltb	lymphotoxin B	1.84	NM_008518	0.02
Lyar	---	2.09	NM_025281	0.04
Lyn	Yamaguchi sarcoma viral (v-yes-1) oncogene homolog	1.86	NM_001111096	0.04
Marco	macrophage receptor with collagenous structure	2.15	NM_010766	0.01
Mcm4	minichromosome maintenance deficient 4 homolog (S. cerevisiae)	2.09	NM_008565	0.04
Mcm5	minichromosome maintenance deficient 5, cell division cycle 46 (S. cerevisiae)	1.87	NM_008566	0.03
Mcm6	minichromosome maintenance deficient 6 (MIS5 homolog, S. pombe) (S. cerevisiae)	1.83	NM_008567	0.0003
Melk	maternal embryonic leucine zipper kinase	1.93	NM_010790	0.02
Mid1	midline 1	2.54	NM_010797	0.01
**Mmp9**	**matrix metallopeptidase 9**	**1.94**	**NM_013599**	**0.02**
Mpzl1	myelin protein zero-like 1	1.59	NM_001083897	0.04
Msh2	mutS homolog 2 (E. coli)	2.31	NM_008628	0.05
Mthfd1l	methylenetetrahydrofolate dehydrogenase (NADP+ dependent) 1-like	1.66	NM_172308	0.05
**Mtrf1l**	**mitochondrial translational release factor 1-like**	**1.52**	**NM_175374**	**0.05**
Mybl2	myeloblastosis oncogene-like 2	2.13	NM_008652	0.03
**Myo10**	**myosin X**	**2.25**	**NM_019472**	**0.05**
Narf	nuclear prelamin A recognition factor	1.54	NM_026272	0.03
Nasp	nuclear autoantigenic sperm protein (histone-binding)	1.94	NM_001081475	0.04
Ncaph	non-SMC condensin I complex, subunit H	1.67	NM_144818	0.03
Ncf2	neutrophil cytosolic factor 2	1.89	NM_010877	0.02
Nek2	NIMA (never in mitosis gene a)-related expressed kinase 2	1.53	NM_010892	0.05
Nf2	neurofibromatosis 2	1.77	L27105	0.02
**Nfib**	**nuclear factor I/B**	**2.52**	**NM_001113209**	**0.007**
**Nfkbie**	**nuclear factor of kappa light polypeptide gene enhancer in B-cells inhibitor, epsilon**	**1.52**	**NM_008690**	**0.05**
Nmb	neuromedin B	1.60	NM_026523	0.006
**Nos3**	**nitric oxide synthase 3, endothelial cell**	**1.65**	**NM_008713**	**0.05**
Npc1	Niemann Pick type C1	1.88	NM_008720	0.04
Oas2	2'-5' oligoadenylate synthetase 2	2.26	NM_145227	0.04
Optn	optineurin	1.56	NM_181848	0.05
Osbpl7	oxysterol binding protein-like 7	1.50	NM_001081434	0.05
Papss1	3'-phosphoadenosine 5'-phosphosulfate synthase 1	2.85	NM_011863	0.04
Parvb	parvin, beta	1.76	NM_133167	0.04
Pdcd5	programmed cell death 5	1.71	NM_019746	0.04

Pdxk	pyridoxal (pyridoxine, vitamin B6) kinase	1.69	NM_172134	0.03
**Pfkl**	**phosphofructokinase, liver, B-type**	**1.85**	**NM_008826**	**0.04**
**Pgk1**	**phosphoglycerate kinase 1**	**2.05**	**NM_008828**	**0.01**
**Pgm2**	**phosphoglucomutase 2**	**2.37**	**NM_028132**	**0.05**
**Pgm3**	**phosphoglucomutase 3**	**2.06**	**NM_028352**	**0.03**
Pitpna	phosphatidylinositol transfer protein, alpha	1.63	NM_008850	0.04
Pitpnm1	phosphatidylinositol transfer protein, membrane-associated 1	2.13	NM_008851	0.02
Pkmyt1	protein kinase, membrane associated tyrosine/threonine 1	3.41	NM_023058	0.01
Plcg2	phospholipase C, gamma 2	1.60	NM_172285	0.03
Plekhb1	pleckstrin homology domain containing, family B (evectins) member 1	2.10	NM_013746	0.03
Plscr1	phospholipid scramblase 1	1.70	NM_011636	0.01
Pola2	polymerase (DNA directed), alpha 2	1.59	NM_008893	0.05
Ppp2r1b	protein phosphatase 2 (formerly 2A), regulatory subunit A (PR 65), beta isoform	2.26	NM_001034085	0.03
Prpf18	PRP18 pre-mRNA processing factor 18 homolog (yeast)	2.54	NM_026045	0.0002
Psmb4	proteasome (prosome, macropain) subunit, beta type 4	1.73	NM_008945	0.01
Psmd2	proteasome (prosome, macropain) 26S subunit, non-ATPase, 2	2.00	NR_027485	0.02
Ptgds	prostaglandin D2 synthase (brain)	2.76	NM_008963	0.04
Ptk7	PTK7 protein tyrosine kinase 7	1.84	NM_175168	0.006
**Rad54l**	**RAD54 like (S. cerevisiae)**	**1.61**	**NM_009015**	**0.003**
Rasgrp2	RAS, guanyl releasing protein 2	1.69	NM_011242	0.04
Rdx	radixin	1.54	NM_009041	0.001
Rhbdl2	rhomboid, veinlet-like 2 (Drosophila)	1.51	NM_183163	0.05
Rhcg	Rhesus blood group-associated C glycoprotein	1.67	NM_019799	0.04
Rnf138	ring finger protein 138	2.09	NM_207623	0.04
Ror1	receptor tyrosine kinase-like orphan receptor 1	3.00	NM_013845	0.005
Rrbp1	ribosome binding protein 1	1.71	NM_024281	0.03
Ryk	receptor-like tyrosine kinase	2.03	NM_013649	0.02
S100a1	S100 calcium binding protein A1	3.49	NM_011309	0.006
S100a10	S100 calcium binding protein A10 (calpactin)	1.64	NM_009112	0.005
Scara3	scavenger receptor class A, member 3	2.22	NM_172604	0.009
Sema4d	sema domain, immunoglobulin domain (Ig), transmembrane domain (TM) and short cytoplasmic domain, (semaphorin) 4D	2.50	NM_013660	0.02
Sft2d2	SFT2 domain containing 2	1.98	NM_145512	0.03
Shfm1	split hand/foot malformation (ectrodactyly) type 1	5.62	NM_009169	0.002
Slc11a1	solute carrier family 11 (proton-coupled divalent metal ion transporters), member 1	1.65	NM_013612	0.008
**Slc2a5**	**solute carrier family 2 (facilitated glucose transporter), member 5**	**1.87**	**NM_019741**	**0.008**
**Slc2a6**	**solute carrier family 2 (facilitated glucose transporter), member 6**	**2.03**	**NM_172659**	**0.01**
Slc39a8	solute carrier family 39 (metal ion transporter), member 8	1.99	NM_001135150	0.01
Slc7a7	---	2.66	NM_011405	0.03
Smc4	structural maintenance of chromosomes 4	1.59	NM_133786	0.005
Speg	SPEG complex locus	2.05	NM_007463	0.006
**Spock2**	**sparc/osteonectin, cwcv and kazal-like domains proteoglycan 2**	**1.54**	**NM_052994**	**0.05**
Srebf2	sterol regulatory element binding factor 2	1.91	NM_033218	0.02
Ssr1	signal sequence receptor, alpha	1.61	NM_025965	0.02

Ssrp1	structure specific recognition protein 1	1.61	NM_182990	0.03
Stac	src homology three (SH3) and cysteine rich domain	2.75	NM_016853	0.02
Steap3	STEAP family member 3	2.26	NM_001085409	0.05
Stk10	serine/threonine kinase 10	2.07	NM_009288	0.02
Syk	spleen tyrosine kinase	1.83	NM_011518	0.01
Tbccd1	TBCC domain containing 1	1.52	NM_001081368	0.03
Tcf7	transcription factor 7, T-cell specific	2.43	NM_009331	0.0009
Tes	testis derived transcript	1.67	NM_207176	0.04
**Tgfbi**	**transforming growth factor, beta induced**	**2.83**	**NM_009369**	**0.002**
**Tgm1**	**transglutaminase 1, K polypeptide**	**1.82**	**NM_019984**	**0.04**
Thoc4	THO complex 4	2.25	NM_011568	0.03
Tia1	cytotoxic granule-associated RNA binding protein 1	1.62	NM_011585	0.01
**Timp2**	**tissue inhibitor of metalloproteinase 2**	**2.06**	**NM_011594**	**0.04**
Tle1	---	2.22	ENSMUST00000107337	0.01
Tle4	transducin-like enhancer of split 4, homolog of Drosophila E(spl)	3.16	NM_011600	0.02
Tlx3	T-cell leukemia, homeobox 3	1.52	NM_019916	0.05
Tmsb10	---	2.08	NM_025284	0.01
**Tnfaip3**	**tumor necrosis factor, alpha-induced protein 3**	**1.71**	**NM_009397**	**0.04**
**Tnfrsf21**	**tumor necrosis factor receptor superfamily, member 21**	**1.61**	**NM_178589**	**0.02**
**Tnfsf13b**	**tumor necrosis factor (ligand) superfamily, member 13b**	**1.51**	**NM_033622**	**0.04**
Topbp1	topoisomerase (DNA) II binding protein 1	1.72	NM_176979	0.05
**Traf1**	**TNF receptor-associated factor 1**	**1.51**	**NM_009421**	**0.02**
Trit1	tRNA isopentenyltransferase 1	1.66	NM_025873	0.01
Trpm5	transient receptor potential cation channel, subfamily M, member 5	2.19	NM_020277	0.03
Ttll4	tubulin tyrosine ligase-like family, member 4	2.01	NM_001014974	0.05
Tubb4	tubulin, beta 4	1.77	NM_009451	0.002
Tubb6	tubulin, beta 6	1.83	NM_026473	0.0001
Ube2c	ubiquitin-conjugating enzyme E2C	1.84	NM_026785	0.006
Uchl3	---	1.53	NM_016723	0.04
Uchl5	ubiquitin carboxyl-terminal esterase L5	1.82	NM_019562	0.04
Ufd1l	ubiquitin fusion degradation 1 like	1.62	NM_011672	0.04
Vcam1	vascular cell adhesion molecule 1	3.25	ENSMUST00000106493	0.01
Vpreb3	pre-B lymphocyte gene 3	1.67	NM_009514	0.03
Vrk2	vaccinia related kinase 2	1.62	NM_027260	0.009
Wars	tryptophanyl-tRNA synthetase	1.94	NM_011710	0.05
Wdr62	WD repeat domain 62	1.60	BC026444	0.04

Key overlapping genes associated with ***metabolism and glycolysis*** (Acot7, Acsl4, Eno1, Gapdh, Ldhb, Mtrf1l, Pfkl, Pgk1, Pgm2, Pgm3, Slc2a5, Slc2a6), ***hypoxia*** (Hyou1), ***the inflammatory response*** (Aif1, C3, Ccl5, Crlf3, Ifngr1, Il10ra, Irak1, Irf5, Isg20, Nfib, Nfkbie, Nos3, Tnfaip3, Tnfrsf21, Tnfsf13b, Traf1), ***myofibroblast differentiation and the extracellular matrix*** (Actl6a, Capg, Col9a3, Dnmt3b, Mmp9, Myo10, Spock2, Tgfbi, Tgm1, Timp2), as well as ***DNA-damage and repair*** (Ddit3, Rad54l), are shown in **BOLD.**
